# Total synthesis of (−)-2-oxo epimesembranol and (+)-dihydromaritidine *via* a key Johnson–Claisen rearrangement†

**DOI:** 10.1039/d4ra05275g

**Published:** 2024-09-16

**Authors:** Satyajit Majumder, Abhinay Yadav, Souvik Pal, Abhishek Mondal, Alakesh Bisai

**Affiliations:** a Department of Chemistry, Indian Institute of Science Education and Research Bhopal Bhauri Bhopal-462 066 Madhya Pradesh India alakeshb@gmail.com; b Department of Chemical Sciences, Indian Institute of Science Education and Research Kolkata Mohanpur Nadia-741 246 West Bengal India alakesh@iiserkol.ac.in

## Abstract

A general approach to *Sceletium* alkaloids of the family *Aizoaceace* following a key Johnson (orthoester)–Claisen rearrangement of an enantioenriched allylic alcohol has been disclosed. The tricyclic core (1c) of *cis*-3*a*-octahydroindoline skeleton was achieved *via* an ester-aminolysis followed by an intramolecular aza-Michael reaction with amine under elevated temperature. Utilizing aforementioned strategy, a collective total syntheses of *Sceletium* alkaloids, such as (−)-2-oxo-epimesembranol (1d) [the first total synthesis], (−)-6-epimesembranol (1b), and (−)-mesembrine (1a) were shown. Further this strategy was applied for total synthesis of (+)-dihydromaritidine (2c) sharing [5,11*b*]-ethanophenanthridine skeleton.

## Abstract 2

The total synthesis of (−)-2-oxo-epimesembranol (11b), (−)-6-epimesembranol (11d), (–)-mesembrine (11e) and dihydromaritidine (2c) has been achieved *via* a ester aminolysis-aza-Michael sequence of an enantioenriched enone intermediate 7b.

## Graphical abstract

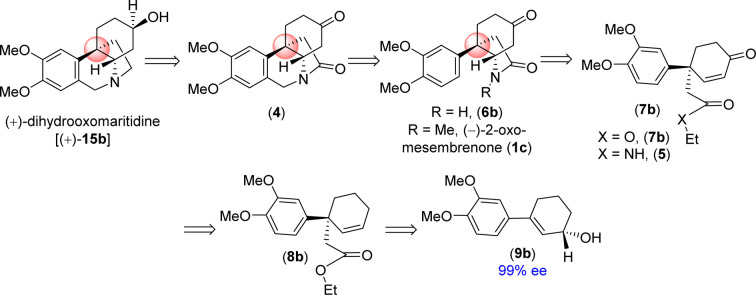

## Introduction

*Amaryllidaceae* alkaloids are a structurally diverse group of plant specialized metabolites with important biological activities.^1^ Plants belonging to the *Amaryllidaceae* and *Sceletium* family are herbaceous perennials that grow from bulbs,^2,3^ More than 500 *Amaryllidaceae* alkaloids have been isolated, with varied biological profiles, from *Amaryllidaceae* plants till date.^3,4^ In particular, the *Sceletium tortuosum*, is an indigenous herb of South Africa, especially in Namaqualand, where the plant is utilized regularly as an herbal supplement in the treatment of central nervous system-related disorders for nearly 200 years,^5,6^ main alkaloid responsible is mesembrine (1a) and mesembranol (1b).^7,8^ They are also cultivated as ornamental plants for their beautiful flowers and to produce volatile oil. These alkaloids contain common core *cis*-3*a*-octahydroindoline skeleton along with a synthetically challenging benzylic all-carbon quaternary streocenter.^9^ Their architecture display vicinal quaternary and tertiary carbon stereocenters^10^ with a fused pyrrolidine ring,^11^ as common structural features, whose stereochemical incorporation is indeed a challenge. As a representative alkaloid of the *Amaryllidaceae* family with significant biological activity, maritidine^12^ is isolated from *Pancratium maritimum*, *Pancratium tortuosum*, and *Zephyranthes* genera,^13^ with a 5,10*b*-ethanophenanthridine nucleus containing dimethoxy substituents at C-8 and C-9 positions. Maritidine is of particular interest due to its cytotoxic properties^14,15^ and limited supplies from natural sources.

It is important to note that, both antipodes of *Amaryllidaceae* alkaloids are naturally occurring. As for example (−)-crinine (2d) and its enantiomer (+)-vittatine (*ent*-2d) are isolated from different *Amaryllidaceae* species.^5,15^ Similarly, naturally occurring (−)-*epi*-crinine (2f) and its enantiomer (+)-*epi*-vittatine (*ent*-2f) are also isolated from different *Amaryllidaceae* species.^5^

The incorporation of sterically congested quaternary center is the critical element in the total synthesis *Amaryllidaceae* alkaloids sharing [5,10*b*]-ethanophenathridine^16^ and *cis*-3*a*-octahydroindoline alkaloids^17,18^ Although great efforts have been devoted to the development of synthetic methods to obtain maritidine type alkaloids, most of the reported approaches provided racemic products,^10^ and only a few asymmetric syntheses of maritidine have been reported.^15,16^

In this regard, the development of a general and efficient asymmetric catalytic method for the concise synthesis of *Amaryllidaceae* and *Sceletium* family of alkaloids having benzylic quaternary stereogenic center has become an important subject in organic chemistry. In this regard, in 2002, Trost and co-workers studied the direct intramolecular Pd(0)-catalyzed asymmetric decarboxylative allylic alkylation of enol carbonates and subsequently, allylation of 2-phenylcyclohexanone (88% ee using L2, 2009).^19*a*,*b*^ However, it has been reported that the utilization of electron-rich aromatics rather very difficult. Kim *et al.*^20*a*^ have reported that only 66% ee was obtained for 3a sharing 3,4-diOMePh as an aryl group using 5.5 mol% of L2 (Fig. 2), clearly indicating that utilization of such process with a substrate sharing electron-donating aromatic rings is indeed a considerable challenge that is worth testing. In 2018, our group also shown *via* an elegant Pd(0)-catalyzed Asymmetric Allylic Alkylations (AAA)^20*b*^ of allyl enol carbonates (3b in 92% ee using L1) (Fig. 2). Further, Wang *et al.*^20*c*^ has reported an elegant Pd(0)-catalyzed asymmetric allylation of α-aryl vinylogous ester 3c (84% ee) using L2 for an asymmetric total synthesis of (−)-oxomaritidine (2b) (Fig. 2).

**Fig. 1:**
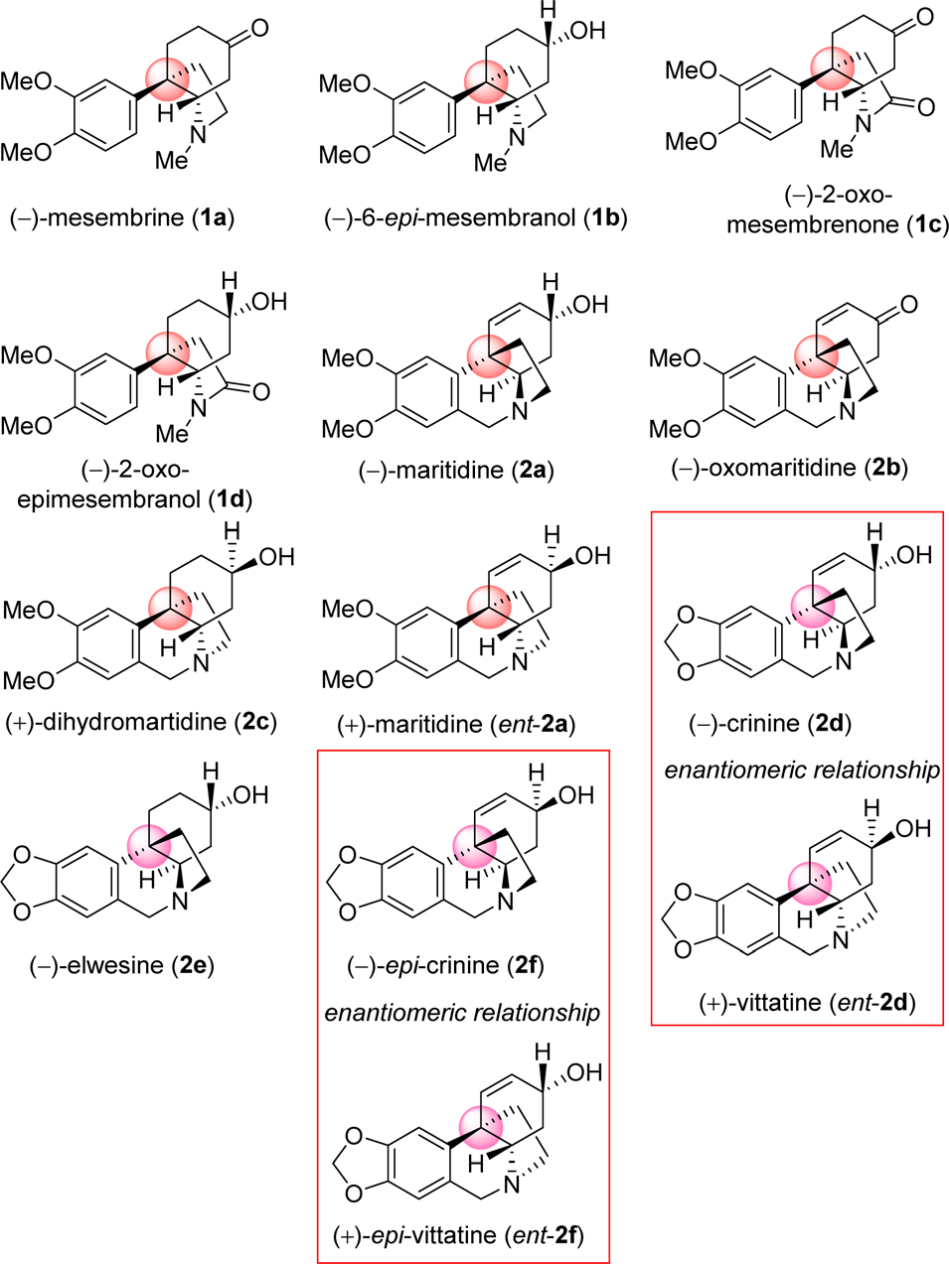
Selected naturally occurring *Sceletium* alkaloids and *Amaryllidaceae* alkaloids.

**Fig. 2:**
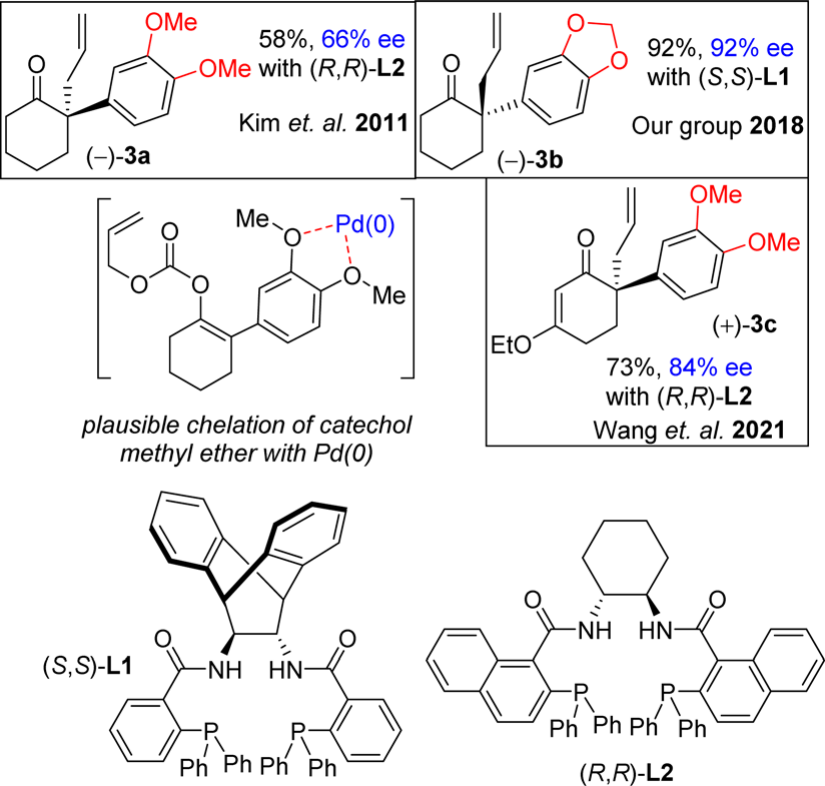
Catalytic enantioselective allylation.

**Scheme 1:**
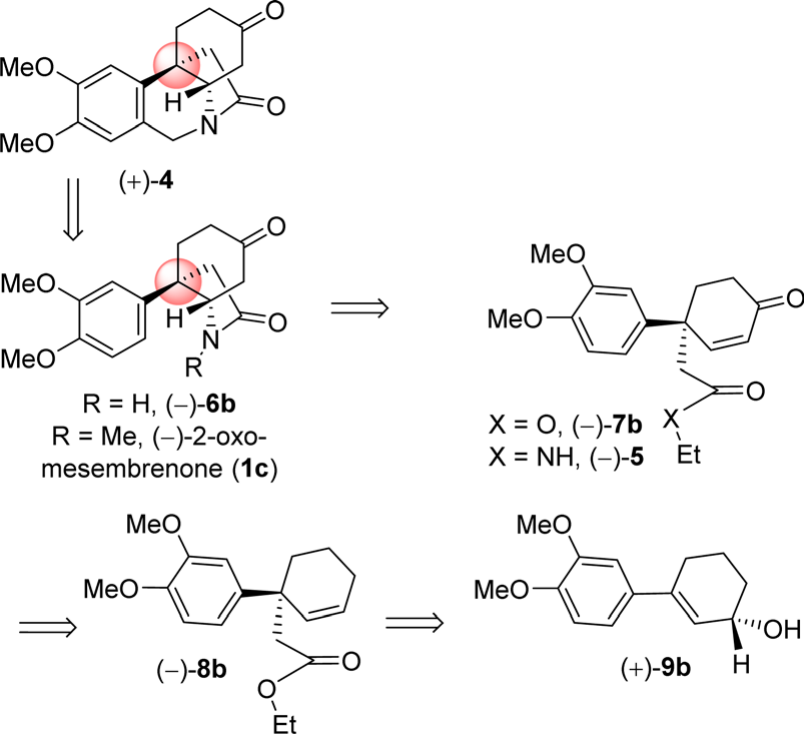
Retrosynthetic analysis.

It is clear from the literature that it is difficult to get high enantioselectivity of electron-rich enol carbonates *via* Pd(0)-catalyzed Asymmetric Allylic Alkylations (AAA). It is believed that a substrate having catechol methyl ether might be co-ordinating with the Pd(0) (see a proposed complex shown in Fig. 2), thereby hampering its catalytic efficiencies in terms of chemical yield (58%) as well as optical purity (66% ee). Thus, a concise catalytic asymmetric approach to the *Amaryllidaceae* alkaloids sharing electron-rich aromatics remains a challenge that is worth pursuing. Retrosynthetically, it was hypothesized that a Johnson–Claisen rearrangement of 3-(3,4-dimethoxyphenyl)cyclohex-2-enol can be an excellent strategic platform to install the all-carbon quaternary stereocenter required for unified strategy for *Sceletium* and *Amaryllidaceae* alkaloids shown in Fig. 1. The retrosynthetic analysis of the asymmetric total synthesis of *cis*-3*a*-octahydroindoline alkaloids is shown in Scheme 1. For a unified approach to the *Scelectium* alkaloids and *Amaryllidaceae* alkaloids, it was envisioned that a Johnson–Claisen rearrangement^21^ of enantioenriched allylic alcohol 9b followed by allylic oxidation (see 7b)^22*a*^ and ester aminolysis (see 5) and aza-Michael reaction (6b and 1c)^23^ to address total synthesis of several congeners of these alkaloids.

**Scheme 2:**
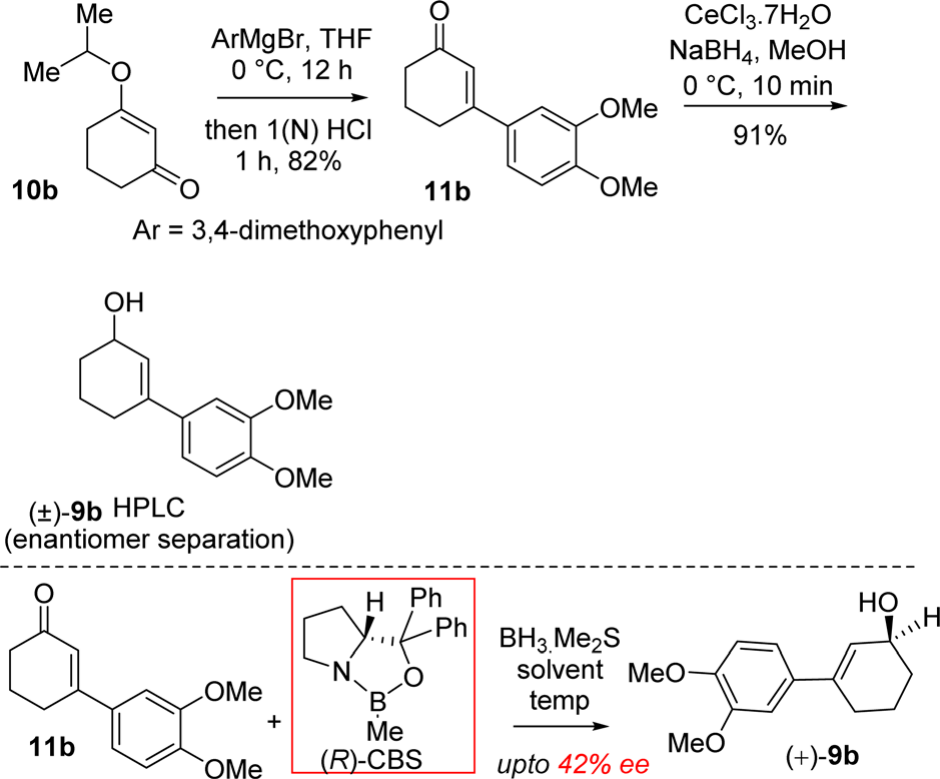
Synthesis of enantioenriched 3-(aryl)cyclohex-2-enols (+)-9b.

It was hypothesized that the advanced intermediate, a benzylic all-carbon quaternary stereocenter containing γ,δ-unsaturated ester 8b, could undergo direct allylic oxidation to generate the substrate for the amidation/transannular aza-conjugate addition reaction, leading to a unified pathway to access both *Sceletium* and *Amaryllidaceae* type alkaloids (Scheme 1). Enone-ester 7b can be synthesized *via* allylic oxidation of cyclohexene 8b, which can be obtained through the Johnson–Claisen rearrangement of allylic alcohol 9b. At this stage, it was proposed that the enantioenriched 3-(aryl)cyclohex-2-enols 9b, which can be accessed through the enantioselective CBS reduction of 3-aryl-2-cyclohexenone 11b (Scheme 2), could provide a pathway to an asymmetric synthesis. Compound 11b can be readily synthesized from vinylogous ester 10b through a well-established Stork–Danheiser sequence.

## Results and discussion

Moving forward with above proposed strategy, it was required to synthesize enantioenriched 3-(aryl)cyclohex-2-enols 9b for the orthoester Johnson–Claisen rearrangement (Scheme 2). Towards this, the Stork–Danheiser sequence on compound 10b using aryl magnesium bromide was carried out to afford 3-aryl 2-cyclohexenone 11b in 82% yields (Scheme 2). Next, it was identified that Corey–Bakshi–Shibata (CBS) reduction was reliable to reduce substituted cyclic enones in high enantioselectivity.

In this regard, we first observed that coordinating polar aprotic solvent THF may reduce the acidity of BH_3_·Me_2_S results it unable to complete catalytic cycle of CBS reduction even in refluxing condition. Later on, we investigated that polar aprotic noncoordinating solvent CH_2_Cl_2_ unable to reduce acidity of BH_3_·Me_2_S results to complete the catalytic cycle of CBS reduction at rt with 20 mol% catalyst. (*R*)-CBS regent was used for the optimization of enantioselective reduction of 3-aryl 2-cyclohexenone 11b.^22*b*^ However, our initial results in this direction were rather discouraging and it was found that even after using 100 mol% (*R*)-CBS regent, a maximum of 42% ee was observed.

**Scheme 3:**
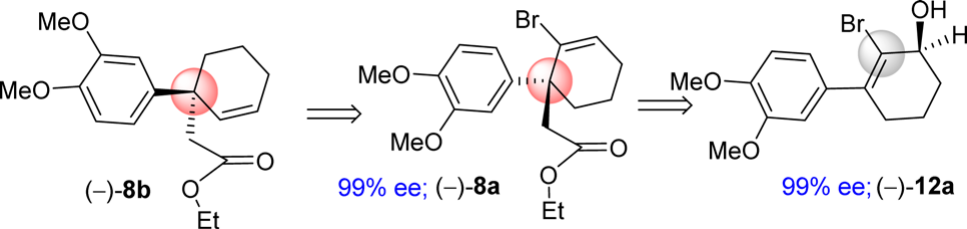
Retrosynthetic analysis.

**Scheme 4:**
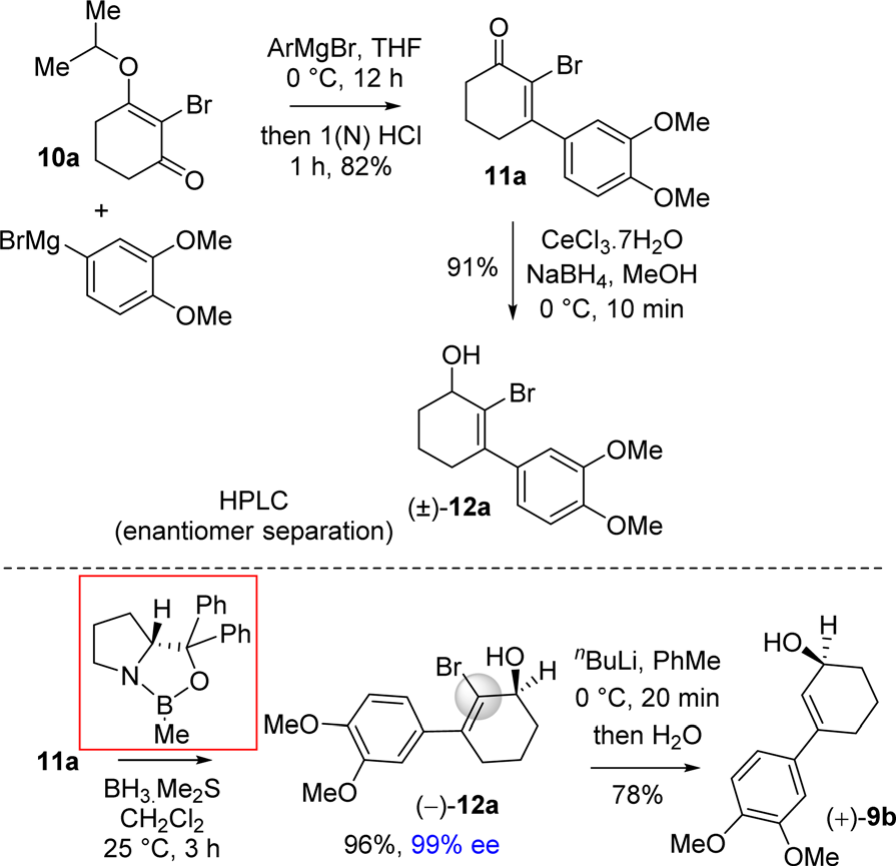
Synthesis of enantioenriched 3-(aryl)cyclohex-2-enols (+)-9b.

It was argued that a sterically crowded easily removable group might help in bringing high enantioselectivity (Scheme 3). Thus, a halogen group such as bromo group that can be easily remove *via* reductive condition was incorporated. Therefore, Stork–Danheiser sequence bromo vinylogous ester 10a was carried out using aryl magnesium bromide to afford 2-bromo-3-aryl 2-cyclohexenone 11a in 82% yields (Scheme 4). Luche reduction of 2-bromo enone provided racemic allyl alcohol 12a for HPLC analysis.

Next, 2-bromo-3-aryl 2-cyclohexenone 11a in hand it was explored under Corey–Bakshi–Shibata (CBS) reduction and the result is summarized in Table 1. The optimization studies were conducted in different solvents such as THF and dichloromethane under different temperature using (*R*)-CBS regent. The initial result using 50 mol% (*R*)-CBS afforded allyl alcohol in 90% ee (entry 3, Table 1). Following exhaustive optimization, it was observed that dichloromethane is a better solvent for this transformation and 100 mol% of (*R*)-CBS in dichloromethane at room temperature afforded allyl alcohol in 97% ee (entry 7, Table 1). Gratifyingly, using 50 mol% of (*R*)-CBS in dichloromethane furnished product in 94% ee (entry 8, Table 1). On further decrease of catalyst loading to 20 mol% of (*R*)-CBS, and under slow reverse addition of bromo-enone 11a, it was found to achieve a 99% ee (entry 9, Table 1).

**Table 1:** Optimization of electron rich bromo-enone CBS reductions

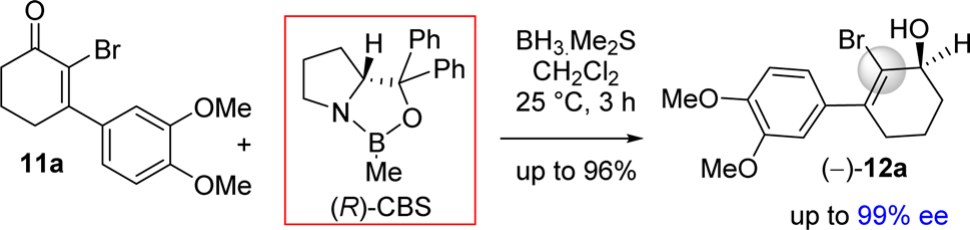						
Entry^a^	(*R*)-CBS reagent	Solvent	Temp.	Time	Yield^b^	% ee^c^
1	100 mol%	THF	0 °C	24 h	0	—
2	100 mol%	THF	25 °C	0.5 h	91%	90%
3	50 mol%	THF	25 °C	0.5 h	50%	90%
4	50 mol%	THF	60 °C	24 h	61%	81%
5	100 mol%	CH_2_Cl_2_	0 °C	24 h	10%	—
6	20 mol%	CH_2_Cl_2_	25 °C	0.5 h	98%	92%
7	100 mol%	CH_2_Cl_2_	25 °C	0.5 h	93%	97%
8	50 mol%	CH_2_Cl_2_	25 °C	2 h	92%	94%
9^d^	20 mol%	CH_2_Cl_2_	25 °C	3 h	94%	99%

a All the reactions were performed under argon atmosphere.

b Yields are reported after column chromatography.

c ee's were measured performing HPLC analysis by Chiralpak OD-H column.

d Slow addition by syringe pump over 3 h.

**Fig. 3:**
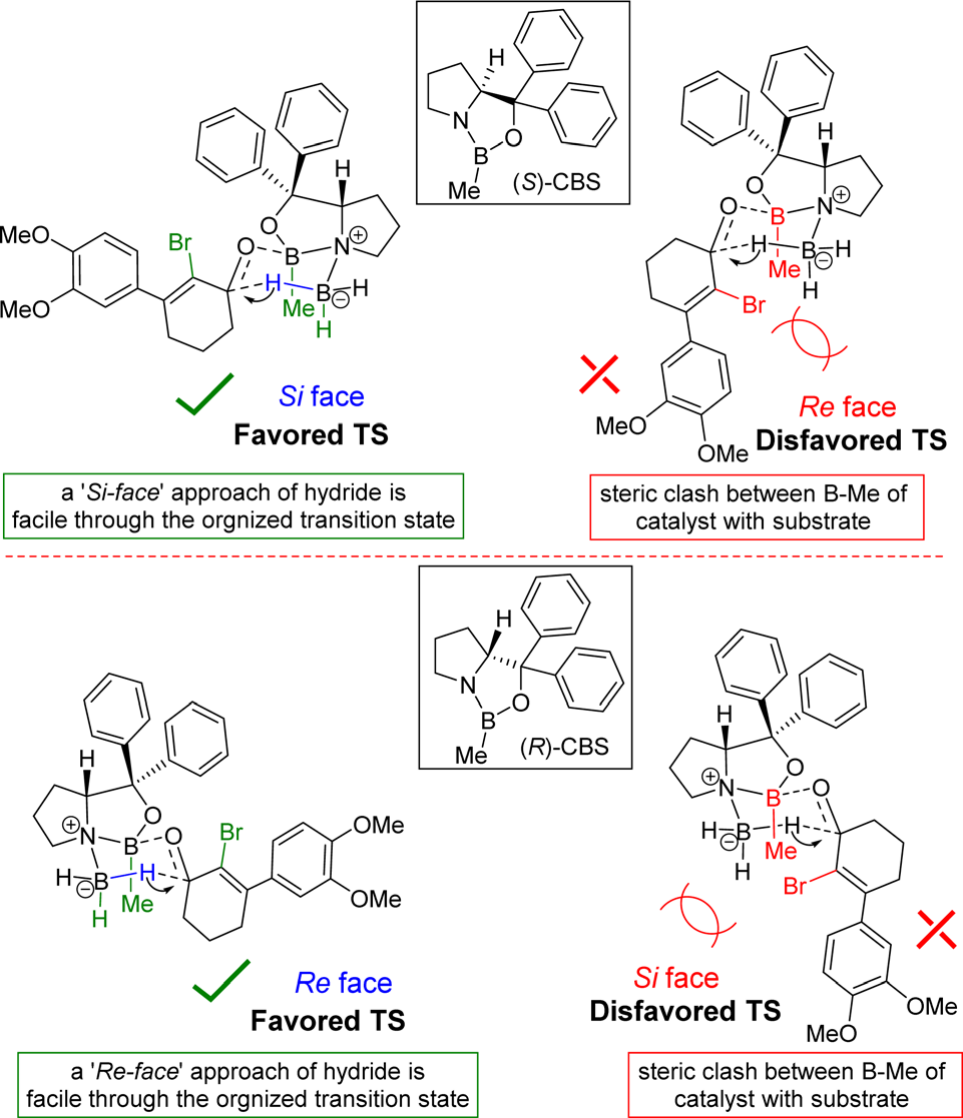
Plausible transition states.

The stereochemical rationale of Corey–Bakshi–Shibata (CBS) reduction of 2-bromo-3-aryl 2-cyclohexenone 11a*via* transition state models are shown in Fig. 3.^22*c*^ It is very clear that the bromide of bromo-enone is imparting enhanced facial bias of the ketone leading to increased enantioselectivities and is subsequently removed *via* a debromination step following the reduction. The asymmetric aspect of this overall transformation is therefore simplified to the preparation of an enantioenriched allylic alcohol. Thus, this account for the perfect enantioselectivity (99% ee) achieved from this reaction. In the favored transition state of CBS reduction [using (*S*)-CBS reagent] of 2-bromo-3-aryl 2-cyclohexenone 11a one could easily account for a ‘*Si*-face’ approach of hydride *via* a six-membered cyclic transition state in achieving optimum enantioselectivity in this process (Fig. 3). It is apparent that a bulky group such as bromo has an important role in controlling enantioselectivity, which in fact helps in retarding the ‘disfavored’ transition state and, thus, avoids ‘*Re*-face’ approach of hydride onto the carbonyl group (Fig. 3, see above). The transition state for the reduction of 2-bromo-3-aryl 2-cyclohexenone 11a using (*R*)-CBS reagent has also been shown in Fig. 3 (see above). In this case a ‘*Re*-face’ approach of hydride *via* a six-membered cyclic transition state is favored, whereas a ‘*Si*-face’ approach of hydride is retarded because of the steric clash (Fig. 3, see above).

The beauty of choosing a bromo group is two-fold in our synthesis: firstly, it helps in achieving highest enantioselectivity and, secondly, this group can easily be removed by the reaction with organometallic reagents such as by the treatment with ^*n*^BuLi followed by quench by water or by a reaction with *n*-Bu_3_SnH in the presence of catalytic AIBN (azo-bis isobutyronitrile) or using Pd(0)-catalyzed cross-coupling with a hydride source. In particular, the bromo group of allyl alcohol was removed by using *n*-BuLi at low temperature to generate vinyl carbanion which was further reacted with water to get allyl alcohol 9b in 98% ee (Scheme 4).

Further, orthoester Johnson–Claisen rearrangement of 3-(aryl)cyclohex-2-enols 9b with triethyl orthoacetate was investigated in different solvent and in presence of catalytic amount of weak acid under heating condition (Table 2). Triethyl orthoacetate was activated in presence of catalytic amount of weak acid under heating condition to produce ketene-acetal intermediate which undergoes [3,3]-sigmatropic rearrangement results γ,δ-unsaturated ester. Initially this reaction was performed in the presence of propanoic acid, pivalic acid, butyric acid and *o*-nitrophenol at different temperatures (Table 2). Following an exhaustive optimization, it was observed that acid catalysed orthoester Johnson–Claisen rearrangement of allyl alcohol 9b afforded product 8b with benzylic all-carbon quaternary stereogenic center in 62% isolated yield when 5 mol% of *o*-nitrophenol was employed as catalyst. The enantioselectivity of product 8b found to be compromised to 68% ee from 99% ee of starting allylic alcohol (entry 6, Table 2).

**Table 2:** Optimization of *ortho*-ester Johnson–Claisen rearrangement of 3-(aryl)cyclohex-2-enols 9b

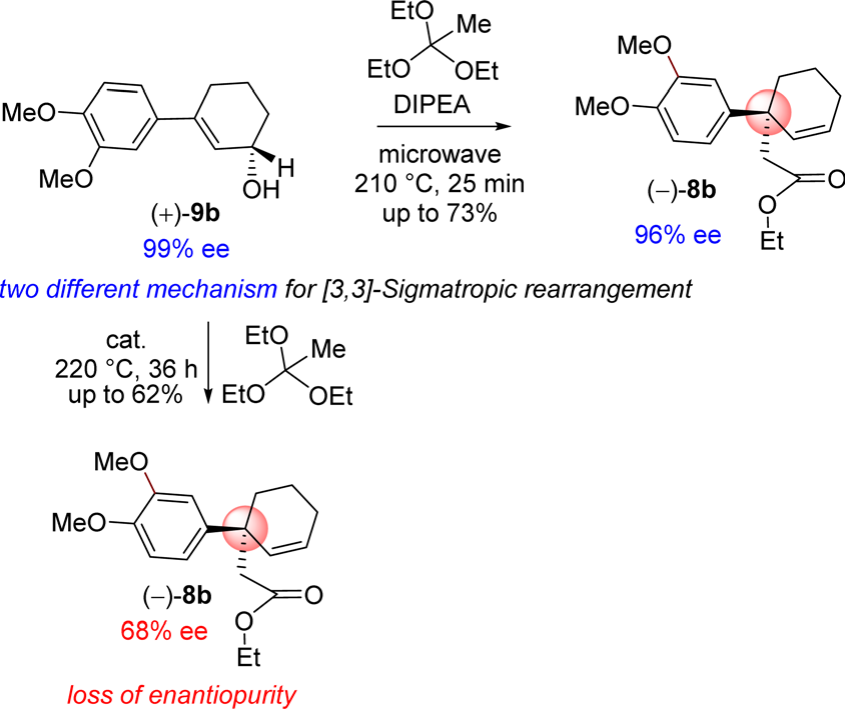					
Entry^a^	Acid	Triethyl orthoacetate	Solvent	Temp. °C	bYield%
1	10 mol% propanoic acid	10 equiv.	Toluene	130 °C	Mixture of products
2	5 mol% propanoic acid	10 equiv.	Xylene	160 °C	Mixture of products
3	5 mol% pivalic acid	10 equiv.	Xylene	140 °C	Mixture of products
4	5 mol% butyric acid	10 equiv.	Xylene	140 °C	Mixture of products
5	5 mol% *o*-nitrophenol	10 equiv.	Xylene	160 °C	Mixture of products
6	5 mol% *o*-nitrophenol	10 equiv.	Xylene	220 °C	62% (68% ee)
7	—	10 equiv.	DIPEA	130 °C	Mixture of products
8	—	10 equiv.	DIPEA	140 °C	20% + 36% (SM)
9	—	10 equiv.	DIPEA	220 °C	73% (97% ee)
10	—	10 equiv.	DIPEA	160 °C	37% + 28% (SM)

a All the reactions were performed under argon atmosphere.

b Yields are reported after column chromatography.

**Scheme 5:**
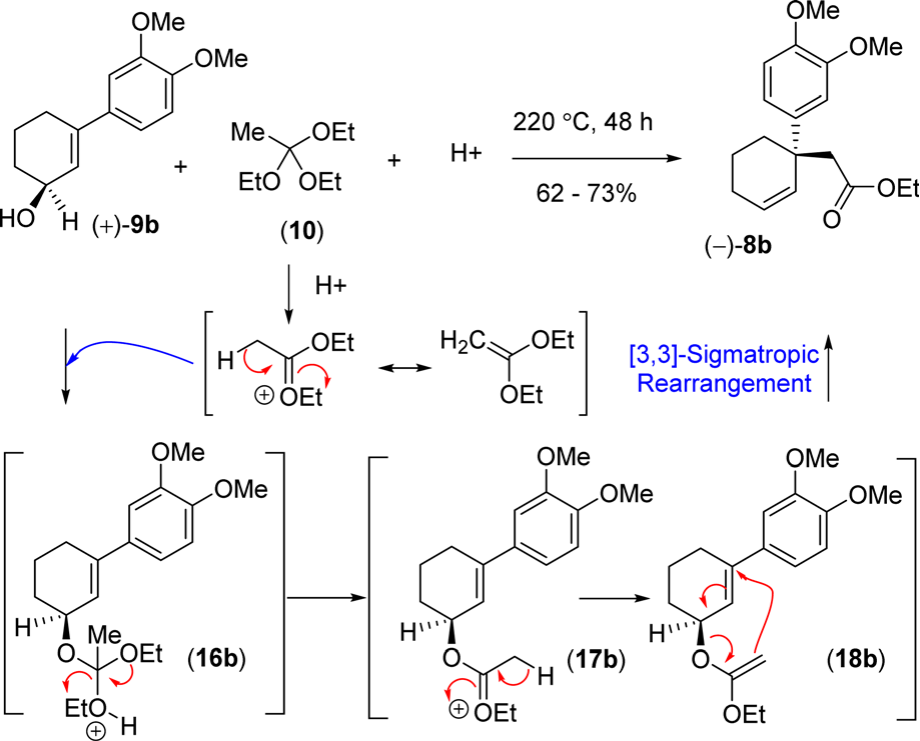
Proposed reaction mechanism of [3,3]-sigmatropic rearrangement.

In order to understand the loss of enantiopurity, it was thought of looking at the mechanism of orthoester Johnson–Claisen rearrangement (Scheme 5). Acid catalyzed activation of triethyl orthoacetate could form an oxocarbenium ion that could react with the allyl alcohol to generate the intermediate 16b responsible for the [3,3]-sigmatropic rearrangement *via* the intermediates 16b–18b (Scheme 5). Since the reaction goes through a concerted pathway, therefore, in principle, an enantioenriched allyl alcohol 9b having >99% ee should provide product 8b in >99% ee *via* an enantioenriched intermediate 18b (>99% ee).

**Scheme 6:**
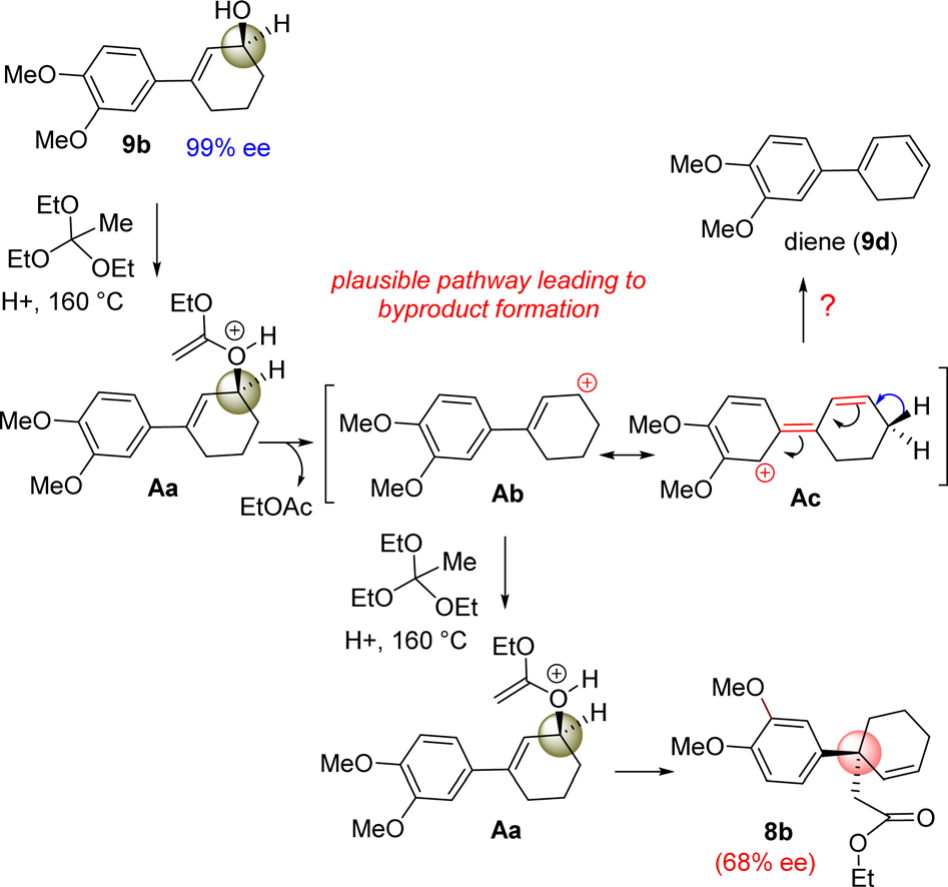
Byproduct formation under orthoester Johnson–Claisen rearrangement of (+)-9b.

However, our result suggests that the electron-rich aromatics probably has a bigger role in the orthoester Johnson–Claisen rearrangement of 3-(aryl)cyclohex-2-enol 9b. It is believed that the enantioselectivity of the enantioenriched intermediate 18b is somehow compromising to 68% ee during the course of the reaction, and therefore, this account for the observed enantioselectivity of 8b as 68% ee (entry 6, Table 2). A rationale of by product formation under this type of pericyclic reaction using electron-rich aromatic ring is shown in Scheme 6.

It is believed that 3-(aryl)cyclohex-2-enols 9b could generate some amount of an allylic carbocation such as Ab (through the intermediate Aa) in the course of reaction (Scheme 6), which is getting stabilized by the electron-donating nature of aromatic ring, in particular, 4-methoxy group of aryl ring present in the 3-position of allyl alcohol (see the formation of Ab from Ac). The allylic carbocation Ab could recombine with ethyl orthoacetate to reform intermediate Ac (68% ee) responsible for the [3,3]-sigmatropic rearrangement product.

**Scheme 7:**
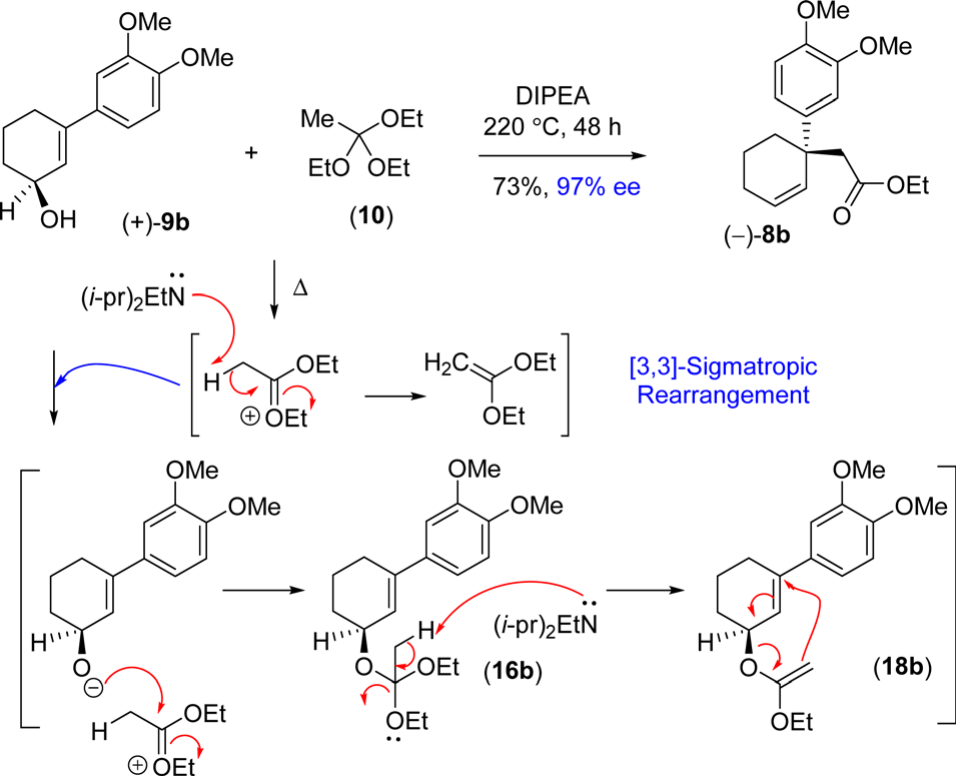
Proposed DIPEA mediated reaction mechanism of [3,3]-sigmatropic rearrangement.

Thus, it was thought of exploring the *ortho*-ester Johnson–Claisen rearrangement of 3-(aryl)cyclohex-2-enols 9b under basic condition (entries 7–10, Table 2). Under the thermal decomposition of triethyl orthoacetate one could able to generate the key intermediate responsible for [3,3]-sigmatropic rearrangement. Thus, Hünig's base *i.e. N*,*N*-diisopropylethylamine (DIPEA) was used a promoter as well as solvent. It was a pleasure to see that, after exhaustive optimization, 10 equiv. of *N*,*N*-diisopropylethylamine (DIPEA) and 10 equiv. of triethyl orthoacetate under heating condition produce γ,δ-unsaturated ester with 73% yield with 97% enantioselectivity.^24^ Interestingly, this reaction afforded enantioenriched [3,3]-sigmatropic rearrangement product (97% ee) with minimum loss of enantiopurity under elevated temperature in the presence of *N*,*N*-diisopropylethylamine (DIPEA) (Scheme 7).^25^

Next, the orthoester Johnson–Claisen rearrangement of 2-bromo 3-(aryl)cyclohex-2-enols 12b was undertaken. Since, 3-(aryl)cyclohex-2-enol having an electron-rich aromatic was difficult, it was thought of exploring the [3,3]-sigmatropic rearrangement of 2-bromo 3-(aryl)cyclohex-2-enol. The reason behind is that the substrate, 2-bromo 3-(aryl)cyclohex-2-enol, has an electron-rich aromatics and at the same time it is having an electron-withdrawing bromo functionality.

**Scheme 8:**
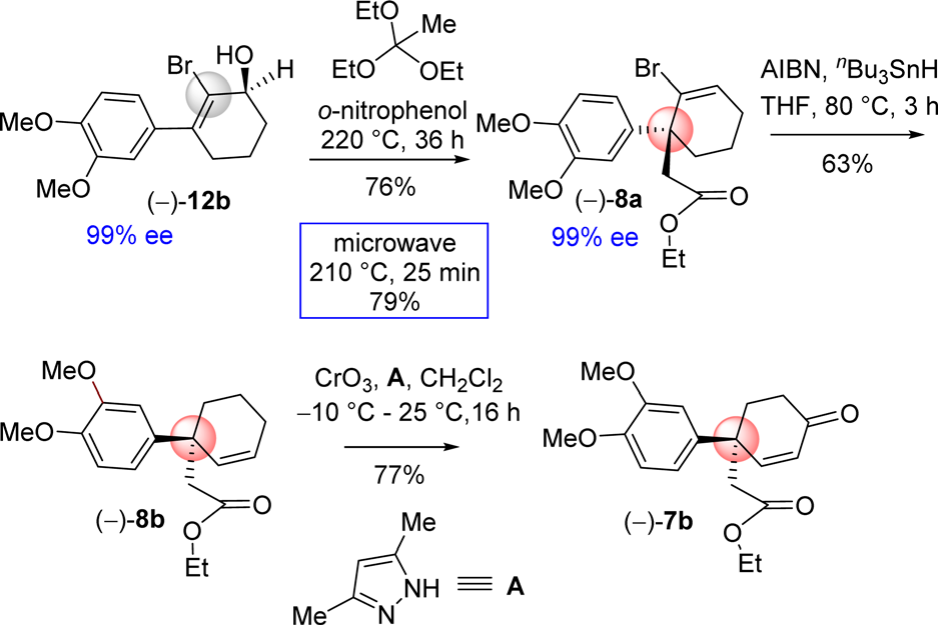
Synthesis of 4-aryl 4-alkyl 2-cyclohexenone (–)-7b.

Based on this intuition, the orthoester Johnson–Claisen rearrangement was carried out under conventional approach *i.e.* under acid catalyzed process. Gratifyingly, it was observed that orthoester Johnson–Claisen rearrangement of 2-bromo 3-(aryl)cyclohex-2-enol was found to be very efficient and afforded product 8a in 76% yield (Scheme 8). Pleasingly, this reaction was found to be more facile under microwave condition to afford product in 79% yield (Scheme 8). Next, debromination of bromo group was conveniently done under classical condition with tri-*n*-butyltin hydride in the presence of AIBN. Subsequent direct conversion of 8b in to the α,β-unsaturated ketone 7b*via* an allylic oxidation (CrO_3_, 3,5-dimethylpyrazole, CH_2_Cl_2_, −10 °C to 25 °C, 16 h, 77%) set the stage for studies on its cyclization.

**Scheme 9:**
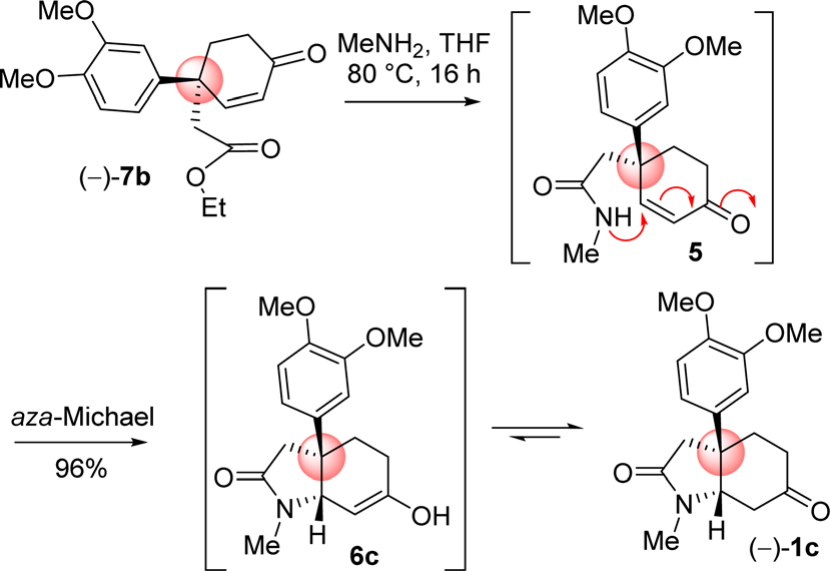
Ester aminolysis and aza-Michael cascade.

4-Aryl 4-alkyl α,β-unsaturated ketone 7b in hand, the efforts were put forward for its conversion into the advanced intermediate that is capable for concise total synthesis of *cis*-3*a*-octahydroindoline alkaloids (Scheme 9). It was hypothesized to synthesize the tricyclic core 1c from 4-aryl 4-alkyl α,β-unsaturated ketone 7b*via* an ester-aminolysis followed by an aza-Michael reaction with methylamine under heating (Scheme 9). Following optimization, it was observed that the treatment of 4-aryl 4-alkyl α,β-unsaturated ketone 7b with MeNH_2_ in THF (80 °C, 16 h) proceeded smoothly furnish bicyclic keto lactam 1c in 96% yield. A rationale of the formation of keto lactam 1c from 4-aryl 4-alkyl α,β-unsaturated ketone 7b is shown in Scheme 9.

Next, chemoselective reduction of keto carbonyl functionality over carboxamide carbonyl was undertaken. In this regard, a number of reducing agents were selected and reduction was performed under different condition (Table 3). Following extensive optimization using various hydride source such as NaBH_4_, LiAlH_4_, Red-Al, and K-selectride, it was found that a highly stereoselective reduction of ketone group is possible to complete the total synthesis of (–)-2-oxoepimesembranol (1d) in excellent yield (99%) and excellent diastereoselectivity (>20 : 1) (Table 3). Thus, a two-step total synthesis of *cis*-3*a*-octahydroindoline alkaloid, (−)-2-oxoepimesembranol (1d) was accomplished from 4-aryl 4-alkyl α,β-unsaturated ketone 7b*via* an ester-aminolysis followed by an aza-Micheal reaction and subsequent chemoselective reduction of keto carbonyl functionality over carboxamide carbonyl.

**Table 3:** Highly stereoselective reduction of ketone functionality

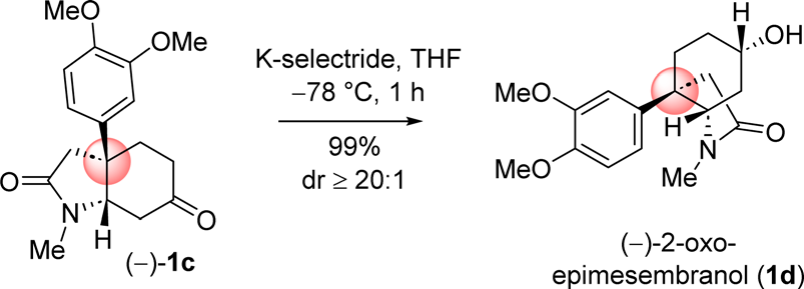						
Entry^a^	Reducing agent	Equivalent	Temp.	Time	Yield^b^	dr
1	NaBH_4_ (MeOH)	1 eq.	0 °C	1 h	84%	∼8 : 1
2	LiAlH_4_ (THF)	1 eq.	0 °C	1 h	70%	∼5 : 1
3	LiAlH_4_ (THF)	1 eq.	−78 °C	1 h	69%	∼10 : 1
4	Red-Al (PhMe)	1 eq.	25 °C	4 h	65%	∼5 : 1
5	K-selectride (THF)	1 eq.	−78 °C	1 h	99%	>20 : 1

a All the reactions were performed under argon atmosphere.

b Yields are reported after column chromatography.

**Scheme 10:**
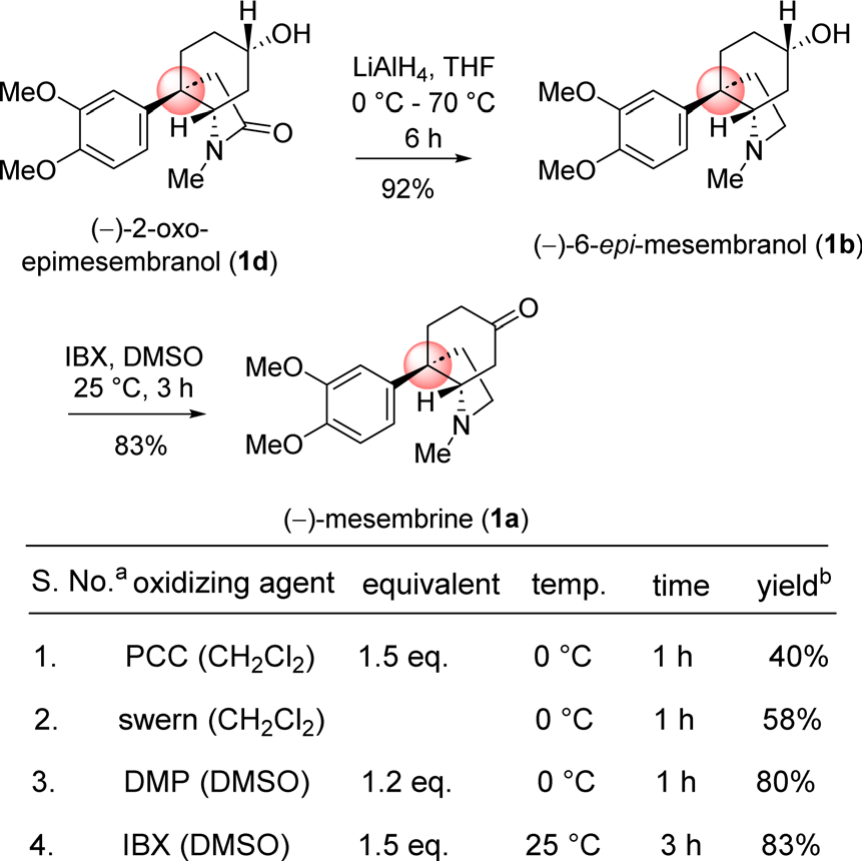
Collective total synthesis of *cis*-3*a*-octahydroindoline alkaloids. ^*a*^All the reactions were performed under argon atmosphere. ^*b*^Yields are reported after column chromatography.

Next, the reductive removal of the lactam carbonyl of 1d upon treatment with LiAlH_4_ (5 equiv, THF, 70 °C reflux, 6 h) completed the total synthesis of (−)-6-epimesembranol (1b) in 92% yield (Scheme 10). Further, the treatment with IBX in DMSO (25 °C, 3 h) affords the (−)-mesembrine (1a) in 83% yield (Scheme 10). It is understood that, further utilization of this intermediate in the collective total synthesis of *cis*-3*a*-octahydroindoline alkaloid could be easily achieved with any kind of electron-rich aromatic ring present in these alkaloids. As both enantiomers [(*R*) as well as (*S*)] of CBS catalyst is commercially available, one could synthesize both antipode of the natural product only by changing the enantiomers of catalyst.

**Scheme 11:**
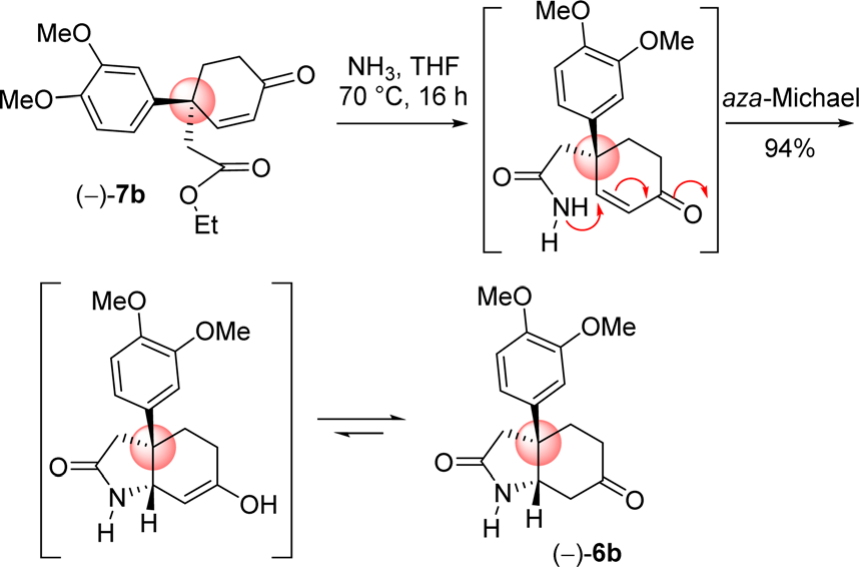
Ester aminolysis with ammonia and aza-Michael cascade.

**Scheme 12:**
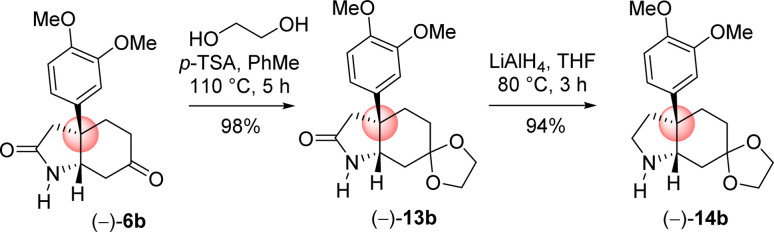
Synthesis of *cis*-3*a*-octahydroindoline skeleton.

Next the total synthesis of naturally occurring dihydromaritidine was undertaken. Towards this end, it was hypothesized to synthesize the tricyclic core 6b from 4-aryl 4-alkyl α,β-unsaturated ketone 7b*via* an ester-aminolysis followed by an aza-Michael reaction with ammonia under heating. Following optimization, it was observed that the treatment of 4-aryl 4-alkyl α,β-unsaturated ketone 7b with NH_3_ in THF (70 °C, 16 h) proceeded smoothly furnish bicyclic keto lactam 6b in 94% yield (Scheme 11). Further, keto functional group of the bicyclic keto lactam 6b was protected with ethylene glycol in the presence of catalytic amount *p*-toluene sulfonic acid to afford 13b in 98% yield (Scheme 12). Later, lithium aluminium hydride reduction in tetrahydrofuran under reflux afforded *cis*-8*a*-octahydroindole derivative 14b in 94% yield. Other reducing agents such as Red-Al, superhydride were proved to be inefficient for this transformation. The work-up process of reduction of 13b (particularly the quench of lithium aluminum hydride) is worth mentioning, which was done under the basic condition using 1(N) NaOH. It was observed that an acidic work up using 0.5(N) HCl always afforded a mixture of spots, probably because of the deprotection of ketal functional group.

**Scheme 13:**
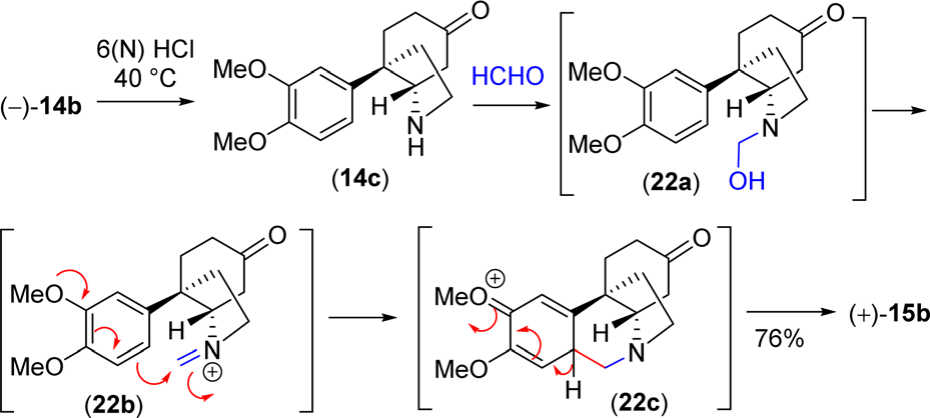
Proposed mechanism for the synthesis of [5,10*b*]-ethanophenanthridine skeleton.

With significant quantity of *cis*-C-8*a*-octahydroindole derivative 14b in hand, the next effort was put forward to affect the Pictet–Spengler cyclization to obtain [5,11*b*]-ethanophenanthridine skeleton (Scheme 13). In this regard, a number of formaldehyde equivalents such as paraformaldehyde, 1,3,5-trioxane, Eschenmoser salt, and formalin were used. Following an exhaustive optimization (Table 4), it was delighted to observe that the synthesis of required [5,11*b*]-ethanophenanthridine skeleton, *i.e.* (+)-dihydrooxomaritidine (15b) could be obtained in 91% from a reaction of 14b and formalin (aqueous solution) in methanol in the presence of 6(N) HCl.

**Table 4:** Pictet–Spengler cyclization of compound 14b

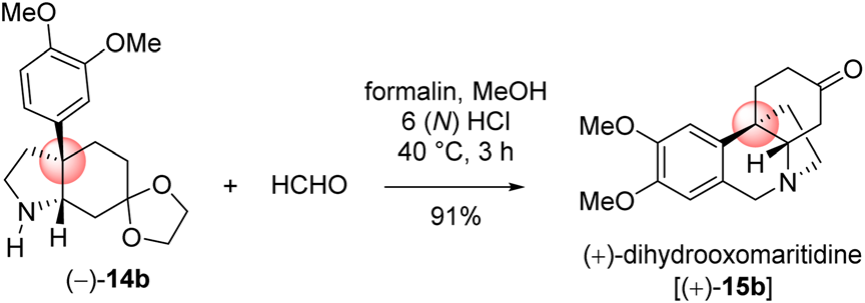					
S. no.^a^	Acid	HCHO equivalent	Temp.	Time	Yield^b^
1	TFA	Paraformaldehyde	0 °C	8 h	62%
2	TFA	1,3,5-Trioxane, BF_3_·OEt_2_	0 °C	8 h	56%
3	None	Eschenmoser salt	40 °C	3 h	65%
4	TFA	Formalin	0 °C	24 h	71%
5	6(N)	Formalin	40 °C	3 h	91%

a All the reactions were performed under argon atmosphere.

b Yields are reported after column chromatography.

A rational of the formation of Pictet–Spengler cyclization product *i.e.* the total synthesis of (+)-dihydrooxomaritidine (15b) from *cis*-3*a*-octahydroindole derivative 14b is shown in Scheme 13. An initial deprotection of ketal functionality in the presence of 6(N) HCl could afford ketone 14c, which on subsequent reaction with formalin could generate a hemi-aminal 22a (Scheme 13). Further activation of the hemi-aminal 22a in the presence of HCl could form iminium intermediate 22b, thereby the stage is ready for the Pictet–Spengler cyclization. Finally, the formation of [5,11*b*]-ethanophenanthridine skeleton, *i.e.* (+)-dihydrooxomaritidine (15b) would complete *via* an aromatic electrophilic substitution type reaction (Scheme 13).

The next effort was to synthesize the naturally occurring *Amaryllidaceae* alkaloids, (+)-dihydromaritidine (2c) sharing [5,11*b*]-ethanophenanthridine skeleton. In this regard a highly diastereoselective reduction of ketone was undertaken (Table 5). Towards this, number of reducing agents such as sodium borohydride, lithium aluminum hydride, Red-Al, and K-selectride, were employed for the total synthesis of (+)-dihydromaritidine (2c). Among various reducing agent, K-selectride was found to be the best and furnished the natural product 2c in 99% yield with >20 : 1 diastereoselectivity (entry 5, Table 5). Gratifyingly, a reduction using sodium borohydride at −10 °C afforded the required (+)-dihydromaritidine (2c) in 98% yield with 10 : 1 dr (entry 1, Table 5).

**Table 5:** Highly stereoselective reduction of ketone functionality

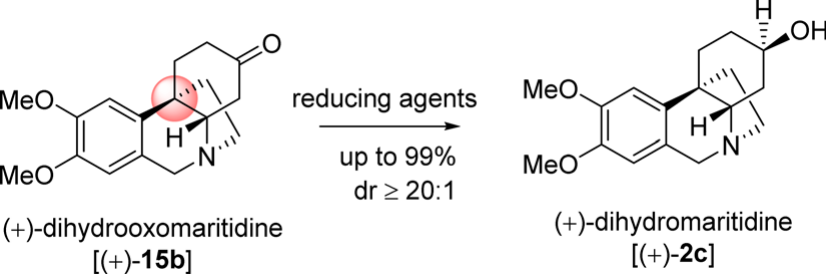						
S. no.^a^	Reducing agent	Equivalent	Temp.	Time	Yield^b^	dr
1	NaBH_4_ (MeOH)	1 eq.	−10 °C	1 h	98%	∼10 : 1
2	LiAlH_4_ (THF)	1 eq.	0 °C	1 h	90%	∼5 : 1
3	LiAlH_4_ (THF)	1 eq.	−10 °C	5 h	69%	∼10 : 1
4	Red-Al (PhMe)	1 eq.	25 °C	4 h	65%	∼10 : 1
5	K-selectride (THF)	1 eq.	−78 °C	1 h	99%	>20 : 1

a All the reactions were performed under argon atmosphere.

b Yields are reported after column chromatography.

In conclusion, we describe a general approach to a number of alkaloids of *Sceletium* alkaloids of the family *Aizoaceace* following Johnson (orthoester)–Claisen rearrangement as the key step. It has been shown that acid catalysed process afforded product with compromised enantioselectivity, whereas a reaction in basic medium (such as diisopropylethylamine, DIPEA) could afford [3,3]-sigmatropic rearrangement product^26^ in 97% ee. Importantly, this reaction installed all carbon quaternary stereocenter at the pseudobenzylic position. The enantioenriched 3-(aryl)cyclohex-2-enol and 2-bromo 3-(aryl)cyclohex-2-enol were prepared by using catalytic enantioselective CBS reduction (up to 99% ee). Utilizing aforementioned strategy, a collective total synthesis of (−)-2-oxo-epimesembranol (1d), (−)-6-epimesembranol (1b), and (−)-mesembrine (1a) were shown. Further utilizing 7b*via* an ester aminolysis with ammonia followed by Pictet–Spengler cyclization leads to completion the total synthesis of (+)-dihydrooxomaritidine (15b) and (+)-dihydromaritidine (2c).

## Conflicts of interest

There are no conflicts to declare.

## Supplementary Material

RA-014-D4RA05275G-s001
