# Supplementary material for: Total synthesis of (−)-2-oxo epimesembranol and (+)-dihydromaritidine via a key Johnson–Claisen rearrangement
Source: RSC Adv. 2024 Sep 16;14(40):29395–403. doi: 10.1039/d4ra05275g (PMC11403583; doi:10.1039/d4ra05275g)
Supplement: RA-014-D4RA05275G-s001 [file RA-014-D4RA05275G-s001.pdf]

Supplementary Information (SI) for RSC Advances.
This journal is © The Royal Society of Chemistry 2024

Supplemental material for:

Total syntheses of (–)-2-oxo epimesembranol and (+)-dihydromaritidine *via* a key Johnson-Claisen rearrangement

Satyajit Majumder,[a] Abhinay Yadav,[a] Souvik Pal,[a] Abhishek Mondal,[b] and Alakesh Bisai*[a,b]

[a]Department of Chemistry, Indian Institute of Science Education and Research Bhopal, Bhauri, Bhopal – 462 066, Madya Pradesh, INDIA

[b]Department of Chemical Sciences, Indian Institute of Science Education and Research Kolkata, Mohanpur Campus, Kalyani, Nadia – 741 246, West Bengal, INDIA

Table of Contents

Materials and Methods	S2
Synthesis of **11a**	S3-S4
Luche reduction of **11a**	S4
CBS reduction of **11a**	S4-S5
Synthesis of (+)-**9b**	S5-S6
Synthesis of (–)-**8b**	S7
Synthesis of (–)-**8a**	S8-S9
Synthesis of (–)-**8b**	S9-S10
Synthesis of (–)-**7b**	S10-S11
Synthesis of (–)-**1c**	S11-S12
Synthesis of of **(–)**-2-oxoepimesembranol (**1d)**	S12-S13
Synthesis of (–)-6-epimesembranol [**1b**]	S14-S15
Synthesis of (–)-mesembrine [**1a**]	S15-S16
Synthesis of (–)-**6b**	S16-S17
Synthesis of (–)-**13b**	S17-S18
Synthesis of (–)-**14b**	S18-S19
Synthesis of (+)-dihydroxomaritidine (**15b**)	S19-S21
Syntheses of (+)-dihydromaritidine [**2c**]	S21-S22

Materials and Methods

All chemicals have been purchased from Spectrochem, Sigma-Aldrich or MARC-Chemical were used without further purification. All reactions have been carried out in oven dried glass were with Teflon-coated magnetic stirring bars were used to stir the reactions. The syringe was used to transfer the solvents and liquid reagents. Tetrahydrofuran (THF), Toluene, were distilled over sodium/benzophenone ketyl. Dichloromethane (CH_2Cl_2) was distilled over calcium hydrate and Diisopropylethylamine (DIPEA) was distilled over KOH. All other solvents like MeOH, EtOAc, Hexane and reagents were used as received. Reaction temperatures above 25 °C were maintained by using oil bath on a magnetic stirrer. All synthetic transformations have been monitored by thin layer chromatography (TLC). Yields refer to purified, dried and spectroscopically pure compounds. TLC was performed on Merck aluminum silica gel 60 F_{254} palates (0.25 mm thickness) pre-coated with a fluorescent indicator, and visualized by UV irradiation, I_2 vapors, DNP stain and other stains. Concentration under reduced pressure was performed by rotary evaporation at 40 °C. Silica gel of particle size 100-200 mesh and basic alumina were used to perform flash chromatography. Digital melting point apparatus is used to record the melting points, with Jyoti Scientific (ANISO 9001:2000) and are uncorrected. All NMR spectra were recorded using a Bruker Avance spectrometer at room temperature. ^{1}H NMR spectra was recorded by using 400 MHz and 500 MHz spectrometers, 13C NMR operating frequency was 100 MHz and 125 MHz. Chemical shifts (δ-values) are reported in ppm, Chemical shifts (δ) are reported in ppm relative to the residual solvent ($CDCl_3$) signal (δ = 7.28 for ^{1}H NMR and δ = 77.0 for ^{13}C NMR), multiplicity is reported as follows: s = singlet, d = doublet, t = triplet, q = quartet, m = multiplet or unresolved and coupling constant *J* in Hz. IR spectra were recorded on a FT-IR Spectrometer system (PerkinElmer Spectrum Two) and are reported in frequency of absorption (cm^{-1}). Only selected IR absorbencies are reported. High-Resolution Mass Spectrometry (HRMS) data was recorded on MicroOTF-Q-II mass spectrometer using methanol as solvent. Enantiomeric excess was determined by chiral HPLC analysis performed on HPLC system with Daicel Chiralpak IC, Daicel Chiralpak OD-H column. Optical rotations $[\alpha]_D$ were measured at the sodium D line using a 2 ml cell with a 1 dm path length or a 0.15 cell with a 0.1 dm path length on a Rudolph Research Analytical Autopol II Automatic Polarimeter and the concentrations c are given in g/100ml.

Stork-Danheiser's sequence of vinylogous ester (10a):

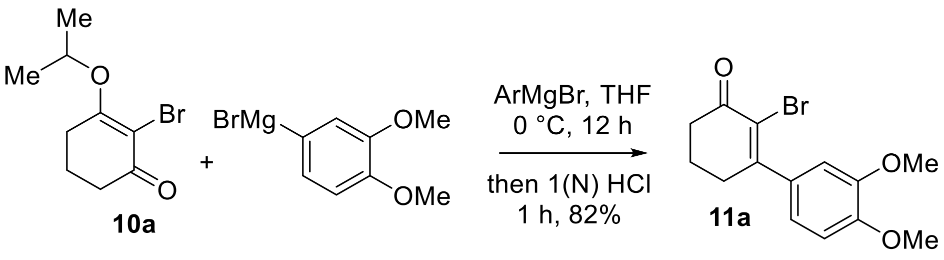

In an oven dried round bottom flask along with one pinch of I_2 and Mg (1.343 gm, 55.928 mmol, 1.2 equiv) was heated slowly with a heat gun in N_2-atmosphere, violet fume of iodine was produced then $C_2H_4Br_2$ (300μl) and 3, 4-dimethoxy bromobenzene (10 gm, 46.069 mmol, 1 equiv) with THF (80 ml, 0.58 M) was added dropwise. Then the heating was continued unless violet color of the solution is decolorized. Then the reaction mixture was left for 4 h in 25 °C with vigorous stirring and maximum amount Mg was consumed. Then compound **10a** (9.08 gm, 41.462 mmol, 0.9 equiv) with dry THF (20 ml, 2.07 M) was added to the reaction mixture at 0 °C. After 8 h the reaction mixture was quenched with 1 (*N*) HCl and allowed to stir 30 minutes, compound was extracted with EtOAc. The combined organic phase was dried over anhydrous Na_2SO_4 and concentrated. The residue was purified by column chromatography (100-200 silica, 20% EtOAc/Hexane) to give pure **11a** (9.75 gm, 68%).

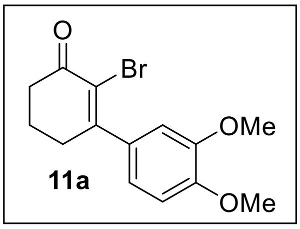

2-Bromo-3',4'-dimethoxy-5,6-dihydro-[1,1'-biphenyl]-3(4*H*)-one (11a): The compound **11a** was obtained as white solid (MP: 68-70 ˚C) (46.0 mmol; 9.75 g; 68%). R_f = 0.40 (30% EtOAc in *n*-hexane).

^{1}H NMR (400 MHz, Chloroform-*d*): δ 6.98 – 6.90 (m, 3H), 3.93 (s, 3H), 3.91 (s, 3H), 2.81 (t, *J* = 6.0 Hz, 2H), 2.72 (t, 2H), 2.21 – 2.10 (m, 2H).

^{13}C{^{1}H} NMR (100 MHz, Chloroform-*d*): δ 191.7, 160.4, 149.6, 148.5, 133.1, 122.1, 120.1, 111.0, 110.7, 56.1, 55.9, 37.8, 35.2, 22.2.

FTIR (thin film, neat, cm^{-1}): 2934, 2835, 1678, 1600, 1510, 1461, 1411, 1323, 1237, 1204, 1181, 1168, 1141, 1082, 1022, 983, 818, 766, 733, 537.

HRMS (ESI-TOF) *m/z*: [M + Na]$^+$ Calcd for [$C_{14}H_{15}BrO_3$ + Na]$^+$ 311.0262; Found 311.0277.

Luche reduction of 2-bromo-3-aryl 2-cyclohexenone (11a):

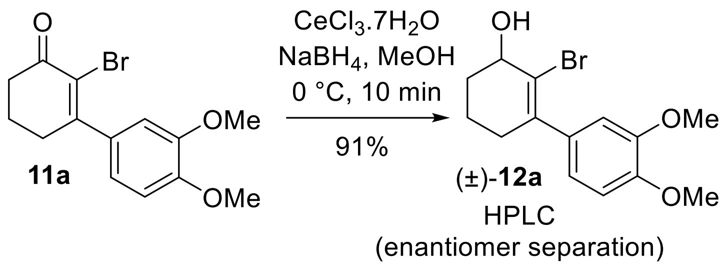

In an oven dried round bottom flask compound **11a** (100 mg, 0.321 mmol, 1.0 equiv.) was taken in MeOH (3 ml) and then allowed to cool to 0 °C. After 10 min of stirring $CeCl_3$, $7H_2O$ (59.80 mg, 0.161 mmol, 0.5 equiv.) was added portion-wise to the reaction mixture followed by the addition of $NaBH_4$ (13.35 mg, 0.353 mmol, 1.1 equiv.). After complete consumption of starting materials (confirmed by TLC) reaction was quenched with saturated NH_4Cl and was extracted with ethyl acetate and washed with brine. The combined organic layer was dried over Na_2SO_4 and concentrated under reduced pressure. The crude product was purified by column chromatography.

Catalytic enantioselective reduction of enone (11a) using (*R*)-CBS-catalyst:

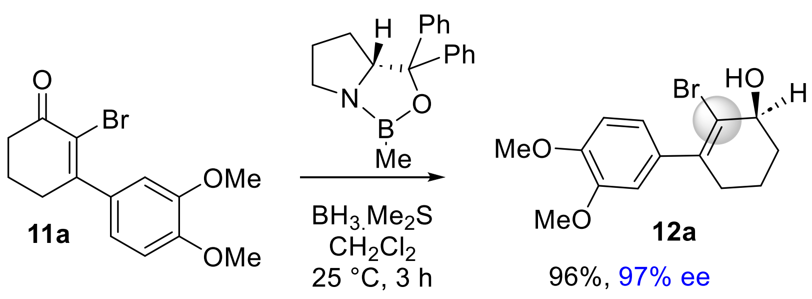

To a stirred solution of (*R*)-CBS (643 µl 1 M in toluene, 0.643 mmol, 0.2 equiv.) in dry CH_2Cl_2 was added $BH_3.Me_2S$ (1.93 ml 2 M in THF, 3.856 mmol, 1.2 equiv.) dropwise at 25 °C. After 10 min of stirring at this temperature, a solution of **11a** (1 gm, 3.21 mmol, 1.0 equiv.) in CH_2Cl_2 was added slowly by syringe pump over 3 h. Following completion of the reaction, the reaction was quenched with 2 (*M*) HCl and extracted with CH_2Cl_2. The combined organic layers were dried over Na_2SO_4 and concentrated. The crude mixture was purified by chromatography (100-200 silica, 10-13% EtOAc/Hexane) to give enantiopure compound **12a** as white solid (966 mg, 96%).

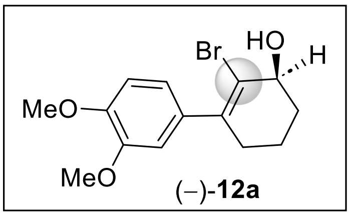

(*S*)-2-Bromo-3',4'-dimethoxy-3,4,5,6-tetrahydro-[1,1'-biphenyl]-3-ol [(–)-12a]: The compound (–)-**12a** was obtained as white solid (MP: 98-100 ˚C) (3.21 mmol; 966 mg, 96%). R_f = 0.40 (30% EtOAc in *n*-hexane).

^{1}H NMR (400 MHz, Chloroform-*d*): δ 6.88 (d, *J* = 8.7 Hz, 1H), 6.81 (dq, *J* = 4.3, 2.0 Hz, 2H), 4.43 (t, *J* = 5.5 Hz, 1H), 3.90 (s, 6H), 2.47 – 2.40 (m, 2H), 2.06 – 1.98 (m, 2H), 1.97 – 1.88 (m, 1H), 1.84 – 1.74 (m, 1H).

^{13}C{^{1}H} NMR (100 MHz, Chloroform-*d*): δ 148.5, 148.3, 141.3, 134.8, 123.5, 120.0, 111.4, 110.8, 71.2, 56.0, 55.8, 34.6, 31.7, 18.6.

FTIR (thin film, neat, cm^{-1}): 3320, 2927, 2836, 1516, 1444, 1404, 1335, 1251, 1209, 1169, 1135, 1055, 1020, 968, 847, 814, 764, 593.

HRMS (ESI-TOF) *m/z*: $[M + Na]^+$ Calcd for $[C_{14}H_{17}BrO_3 + Na]^+$ 335.0227; Found 335.0253.

Enantiomeric excess of pure compound was determined *via* HPLC analysis using a Chiralpak IC column; solvent: hexane/2-propanol = 90/10; flow rate: 1.0 mL/min; detection: at 254 nm): t_R minor = 16.248 min, t_R major = 19.983 min. $[\alpha]_D^{21.0}$ = (–)-54.17 (*c* = 0.84, $CHCl_3$ for 97% ee).

Debromination of 2-bromo-3-aryl 2-cyclohexen-1-ol [(–)-12a]:

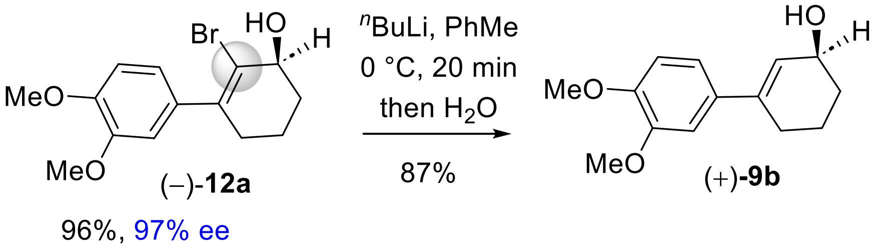

To a solution of **12a** (1 gm, 3.193 mmol, 1.0 equiv.) in dry toluene ^{n}BuLi (5.11 ml 2.5 M in Hexane, 12.77 mmol, 4.0 equiv.) was added drop wise at 25 °C. The resulting reaction mixture was quenched after 20 minutes by cooled H_2O, compound was extracted with EtOAc. The combined organic phase was dried over anhydrous Na_2SO_2 and concentrated. The residue was purified by column chromatography (100-200 silica, 20% EtOAc/Hexane) to give pure **9b** (650.81 mg, 87%).

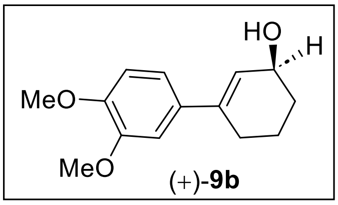

(*S*)-3',4'-Dimethoxy-3,4,5,6-tetrahydro-[1,1'-biphenyl]-3-ol [(+)-9b]: The compound **9b** was obtained as white solid (mp: 90-92 ˚C) (3.193 mmol; 650.81 mg, 87%). R_f = 0.20 (30% EtOAc in *n*-hexane).

^{1}H NMR (400 MHz, Chloroform-*d*): δ 6.97 (d, *J* = 5.6 Hz, 2H), 6.84 (d, *J* = 8.8 Hz, 1H), 6.08 (d, *J* = 2.0 Hz, 1H), 4.39 (t, *J* = 7.2 Hz, 1H), 3.91 (s, 3H), 3.89 (s, 3H), 2.50 – 2.32 (m, 2H), 1.99 – 1.87 (m, 2H), 1.77 – 1.65 (m, 2H).

^{13}C{^{1}H} NMR (175 MHz, Chloroform-*d*): δ 148.7, 148.6, 139.6, 134.3, 125.3, 117.8, 110.9, 108.7, 66.4, 55.9, 55.8, 31.7, 27.6, 19.5.

FTIR (thin film, neat, cm^{-1}): 3368, 2930, 1642, 1581, 1515, 1460, 1416, 1255, 1167, 1144, 1025, 805, 764.

HRMS (ESI-TOF) *m/z*: $[M + Na]^+$ Calcd for $[C_{14}H_{18}O_3 + Na]^+$ 257.1132; Found 257.1148.

Enantiomeric excess of pure compound was determined *via* HPLC analysis using a Chiralpak IC column; solvent: hexane/2-propanol = 70/30; flow rate: 1.0 mL/min; detection: at 254 nm): t_R major = 10.995 min, t_R minor = 14.159 min. $[\alpha]_D^{21.0}$ = (+) 5.08 (c = 0.56, $CHCl_3$ for 97% ee).

Orthoester Johnson−Claisen rearrangement of allylic alcohol (+)-9b:

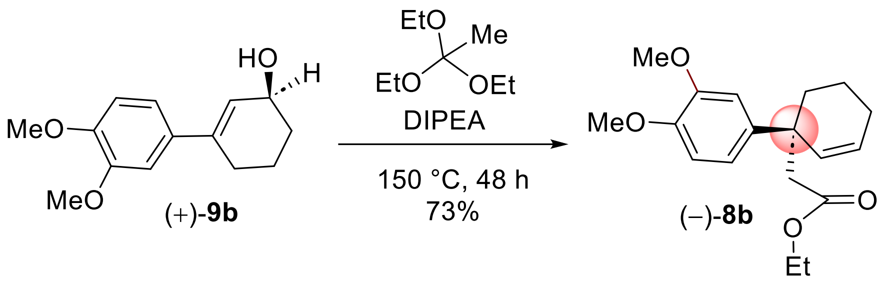

To a solution of **9b** (500 mg, 2.134 mmol, 1.0 equiv.) in *N*,*N*-diisopropylethylamine (3.653 ml, 21.34 mmol, 10 equiv.) and triethyl orthoacetate (3.921 ml, 21.34 mmol, 10 equiv.), anhydrous Na_2SO_4 (200 mg, 1.41 mmol, 0.66 equiv.) was added in sealed tube. The resulting solution was allowed to stir for 48 h in a preheated 152 °C oil bath. Then the reaction mixture was cooled down to RT and 10 ml 2 (N) HCl was added and allowed to stir 30 min, compound was extracted with EtOAc. The combined organic phase was dried over anhydrous Na_2SO_4 and concentrated. The residue was purified by column chromatography (100-200 silica, 4% EtOAc/*n*-hexane) to give pure **8b** (443.45 mg, 73%).

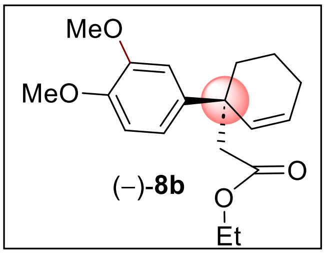

Ethyl (*R*)-2-(3',4'-dimethoxy-3,4-dihydro-[1,1'-biphenyl]-1(2*H*)-yl)acetate [(–)-8b]: The compound **8b** was obtained as colorless oil (2.134 mmol; 443.45 mg, 68%). R_f = 0.30 (10% EtOAc in *n*-hexane).

^{1}H NMR (400 MHz, Chloroform-*d*): δ 6.91 – 6.83 (m, 2H), 6.82 – 6.75 (m, 1H), 6.14 – 6.05 (m, 1H), 5.92 (dt, *J* = 10.2, 3.6 Hz, 1H), 3.97 (qd, *J* = 7.2, 1.5 Hz, 2H), 3.86 (s, 3H), 3.85 (s, 3H), 2.75 (d, *J* = 14.2 Hz, 1H), 2.66 (d, *J* = 14.0 Hz, 1H), 2.04 – 1.98 (m, 2H), 1.97 – 1.91 (m, 1H), 1.89 – 1.81 (m, 1H), 1.59 – 1.50 (m, 1H), 1.42 – 1.32 (m, 1H), 1.08 (t, 3H).

^{13}C{^{1}H} NMR (100 MHz, Chloroform-*d*): δ 171.3, 148.4, 147.1, 139.5, 132.3, 128.4, 119.2, 110.6, 110.5, 59.9, 55.9, 55.8, 47.1, 41.6, 37.1, 25.1, 18.7, 14.1.

FTIR (thin film, neat, cm^{-1}): 2932, 2834, 1726, 1603, 1588, 1515, 1445, 1443, 1253, 1235, 1174, 1146, 1027, 1174, 1095, 806, 766, 732.

HRMS (ESI, m/z): calculated for $C_{18}H_{24}O_4Na$ [M+Na]$^+$: 305.1751, found 305.1747.

Enantiomeric excess of pure compound was determined *via* HPLC analysis: HPLC (OD-H, 2-proprnol/n-hexane = 2/98, flow rate = 1.0 mL/min, I = 254 nm) t_R = 12.3 min (minor), 17.7 min (major). $[\alpha]_D^{23.5}$ = (–) 49.3 (*c* = 0.65, $CHCl_3$ for 91% ee).

Orthoester Johnson−Claisen rearrangement of allylic alcohol (–)-12b:

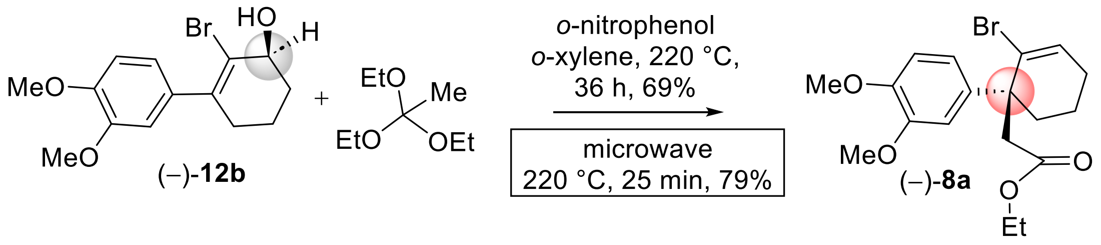

Compound **12b** (200 mg, 0.6386 mmol, 1.0 equiv.) was taken in triethyl orthoacetate (2.34 mL, 12.772 mmol, 20 equiv.) and 2.34 mL of o-xylene in a sealed tube. Then o-nitrophenol (3 mg, 0.0192 mmol, 0.03 equiv.) was added to it. The resulting solution was allowed to stir in a preheated 220 °C oil bath. Upon completion (monitored by TLC analysis; 36 h), the reaction

mixture was cooled down to room temperature and was extracted with EtOAc (7 mL × 2). The combined organic phases were dried over anhydrous Na_2SO_4 and concentrated in vacuo. The residue was purified by column chromatography (100−200 mesh silica, 4% EtOAc/*n*-hexane) to afford **8a** (169 mg, 69% yield).

Orthoester Johnson−Claisen Rearrangement of (–)-12b under Microwave Irradiation: Compound **12b** (200 mg, 0.6386 mmol, 1.0 equiv.) was taken in triethyl orthoacetate (2.34 mL, 12.772 mmol, 20 equiv.) and 2.34 mL of o-xylene in a microwave reaction vial. Then o-nitrophenol (3 mg, 0.0192 mmol, 0.03 equiv.) was added to it. The resulting solution was charged under microwave irradiation maintaining the temperature at 220 °C for 25 min. After 25 min, the reaction mixture was cooled down to room temperature and was extracted with EtOAc (7 mL × 2). The combined organic phases were dried over anhydrous Na_2SO_4 and concentrated in vacuo. The residue was purified by column chromatography (100−200 mesh silica, 4% EtOAc/*n*-hexane) to afford **(–)-8a** (192 mg, 79% yield).

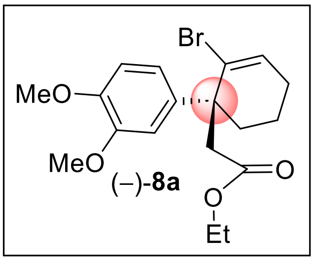

Ethyl (*R*)-2-(6-bromo-3',4'-dimethoxy-3,4-dihydro-[1,1'-biphenyl]-1(2*H*)-yl)acetate [(–)-8a]: The compound **8a** was obtained as light yellow gel. R_f = 0.45 (10% EtOAc in *n*-hexane).

^{1}H NMR (500 MHz, $CDCl_3$): δ 6.96 – 6.93 (m, 2H), 6.86 (d, *J* = 9.0 Hz, 1H), 6.47 (dd, *J* = 5.3, 2.9 Hz, 1H), 4.20 (dd, *J* = 14.0, 7.1 Hz, 2H), 3.91 (s, 3H), 3.89 (s, 3H), 3.07 (d, *J* = 15.9 Hz, 1H), 3.00 (d, *J* = 16.0 Hz, 1H), 2.54 (td, *J* = 13.0, 3.0 Hz, 1H), 2.27 – 2.20 (m, 1H), 2.16 (q, *J* = 4.6, 3.8 Hz, 1H), 1.99 – 1.94 (m, 1H), 1.59 – 1.43 (m, 2H), 1.32 (d, *J* = 7.0 Hz, 3H).

^{13}C{^{1}H} NMR (126 MHz, $CDCl_3$): δ 171.0, 148.5, 147.7, 137.3, 133.8, 128.2, 119.5, 110.8, 110.7, 60.3, 56.0, 55.8, 48.4, 44.3, 37.6, 27.9, 18.0, 14.2.

FTIR (thin film, neat, cm^{-1}): 2939, 2820, 1732, 1548, 1512, 1474, 1219, 1137, 1071, 964, 923, 841, 734, 721, 532.

HRMS (ESI-TOF) *m/z*: $[M + Na]^+$ Calcd for $[C_{18}H_{23}BrO_4 + Na]^+$ 405.0672; found 405.0673.

Enantiomeric excess of pure compound was determined *via* HPLC analysis: HPLC (IC-3, 2-proprnol/n-hexane = 15/85, flow rate = 1.0 mL/min, I = 254 nm) t_R = 10.1 min (major), 17.5 min (minor). $[\alpha]_D^{25.0}$ = (–) 23.48 (*c* = 0.01, $CHCl_3$ for 97% *ee*).

Synthesis of (–)-8b from vinyl bromo derivative (–)-8a:

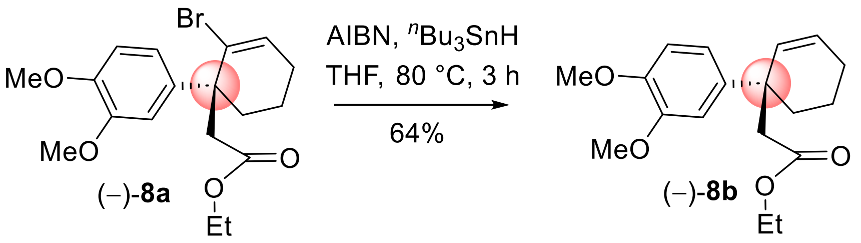

To a solution of compound **8a** (100 mg, 0.2609 mmol, 1.0 equiv.) in THF (3 mL) was charged with tributyltin hydride (85 µL, 0.3131 mmol, 1.2 equiv.) followed by AIBN (5 mg, 0.0261 mmol, 0.1 equiv.) at 25 °C and then the reaction mixture was allowed to reflux at 80 °C for 3 h. Upon complete consumption of the starting material (monitored by TLC), the volatiles were removed under reduced pressure and the resulting residue was purified by column chromatography (100–200 silica, 3% EtOAc in *n*-hexane) to afford pure **8b** (51 mg, 64% yield).

Allylic oxidation of cyclohex-2-ene [(–)-8b]:

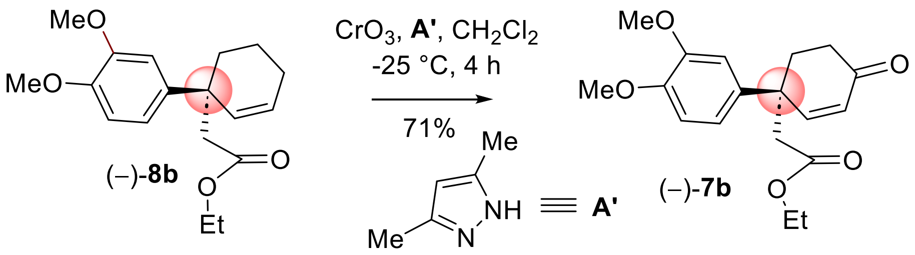

In a dry long neck round bottom flux chromium trioxide (1.64 g, 16.36 mmol, 10 equiv) and 3,5-dimethylpyrazol (1.57 g, 16.36 mmol, 10 equiv) was taken then at –10 °C dry CH_2Cl_2 (6

ml) was added after 30-minute compound **8b** (500 mg, 1.636 mmol, 1 equiv) in CH_2Cl_2 (4 ml) was added dropwise to the reaction mixture then slowly increase temperature to RT. The mixture was left stirring for 16 h at RT then the reaction mixture was diluted with Et_2O and stirred for additional 30 minute. The resulting mixture was filtered through a pad of celite and concentrated. The residue was purified by column chromatography (basic alumina, 20% EtOAc in *n*-hexane) to give pure **7b** (368.8 mg, 71%).

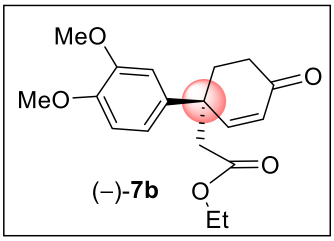

Ethyl (*R*)-2-(3',4'-dimethoxy-4-oxo-3,4-dihydro-[1,1'-biphenyl]-1(2*H*)-yl)acetate [(–)-7b]: The compound **7b** was obtained as colorless gel (1.636 mmol; 368.8 mg, 71%). R_f = 0.20 (20% EtOAc in *n*-hexane).

^{1}H NMR (400 MHz, Chloroform-*d*): δ 7.46 (d, *J* = 10.4 Hz, 1H), 6.88 – 6.81 (m, 3H), 6.22 (d, *J* = 10.3 Hz, 1H), 4.03 (q, *J* = 7.1 Hz, 2H), 3.88 (s, 3H), 3.88 (s, 3H), 2.92 (d, *J* = 14.8 Hz, 1H), 2.82 (d, *J* = 14.8 Hz, 1H), 2.40 – 2.23 (m, 4H), 1.13 (t, *J* = 7.1 Hz, 3H).

^{13}C{^{1}H} NMR (100 MHz, Chloroform-*d*): δ 198.9, 170.3, 154.6, 149.0, 148.1, 134.4, 129.4, 119.1, 111.0, 109.8, 60.6, 56.0, 55.8, 46.0, 42.6, 36.4, 34.4, 14.1.

FTIR (thin film, neat, cm^{-1}): 2935, 2836, 1726, 1677, 1603, 1516, 1462, 1369, 1329, 1147, 1095, 1025, 903, 855, 808, 768, 646.

HRMS (ESI, m/z): calculated for $C_{18}H_{22}O_5Na$ $[M+Na]^+$: 319.1543, found 319.1540.
$[\alpha]_D^{24.0}$ = –36.5 (c = 1, $CHCl_3$)

Ester aminolysis of (–)-7b followed by *aza*-Michael cyclization:

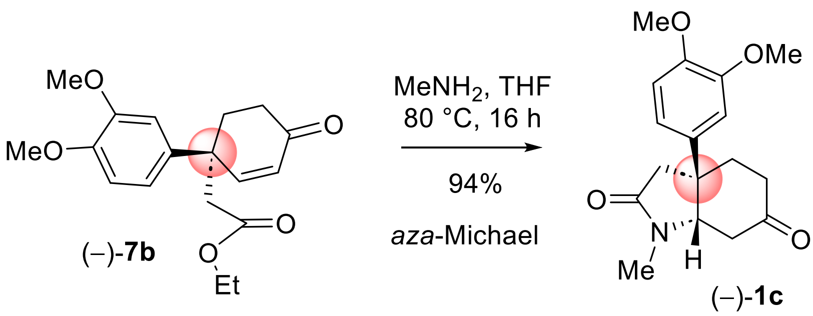

Compound **7b** (200 mg, 0.628 mmol, 1 equiv) was dissolved with CH_3NH_2 (4.08 ml, 2.0 M in THF, 13 equiv), then the resulting mixture was heated to 80 °C in a sealed tube and left stirring for 16 h, at that temperature. The reaction was cooled down to RT and concentrated. The residue was purified by column chromatography (basic alumina, EtOAc in *n*-hexane = 3:2) to give pure **1c** (179.07 mg, 94%).

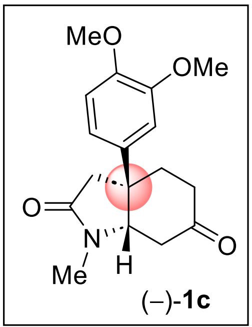

(3a*S*,7a*S*)-3a-(3,4-Dimethoxyphenyl)-1-methylhexahydro-1*H*-indole-2,6-dione [(−)-1c]: The compound **1c** was obtained as colorless sticky gel (0.628 mmol; 179.07 mg, 94%). R_f = 0.50 (EtOAc only).

^{1}H NMR (400 MHz, Chloroform-*d*): δ 6.87 (s, 2H), 6.79 (s, 1H), 4.34 (t, *J* = 4.3 Hz, 1H), 3.90 (s, 3H), 3.90 (s, 3H), 2.92 – 2.85 (m, 2H), 2.86 (s, 3H), 2.81 – 2.71 (m, 2H), 2.42 – 2.29 (m, 3H), 2.27 – 2.19 (m, 1H).

^{13}C{^{1}H} NMR (100 MHz, Chloroform-*d*): δ 208.91, 172.88, 149.46, 148.23, 138.21, 117.50, 111.34, 109.25, 64.57, 56.13, 55.98, 46.21, 41.43, 40.95, 35.92, 34.91, 27.50.

FTIR (thin film, neat, cm^{-1}): 2935, 2836, 1716, 1681, 1588, 1518, 1461, 1398, 1344, 1252, 1146, 1023, 807, 768, 731.

HRMS (ESI, m/z): calculated for $C_{17}H_{21}NO_4Na$ $[M+Na]^+$: 304.1557, found 304.1543.

$[\alpha]_D^{21.5}$ = –69.35 (c = 0.31, $CHCl_3$)

Total synthesis of (–)-2-oxo-*epi* mesembranol (1d)

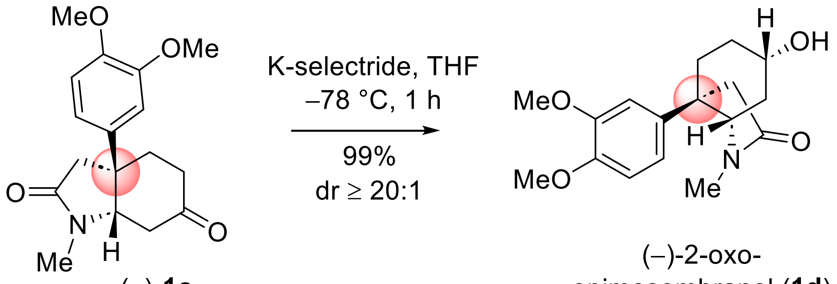

To a solution of **1c** (100 mg, 0.330 mmol, 1 equiv) in dry THF was added dropwise K-selectride (362.61 µl 1 M in THF, 0.363 mmol, 1.1 equiv) at –78 °C and stirring was continued for 1 h at the same temperature and the reaction was quenched by adding MeOH at –78 °C. slowly allowed to worm RT and then the solvent was removed in vacuo and the residue was dissolved with EtOAc and washed with water. Extracted with EtOAc and the combined organic phase was dried over anhydrous Na_2SO_4 and concentrated. The residue was purified by flash column chromatography (basic alumina, 4% MeOH/EtOAc) to give pure (–)-2-oxoepimesembranol (**1d**) (98.65 mg, 99%).

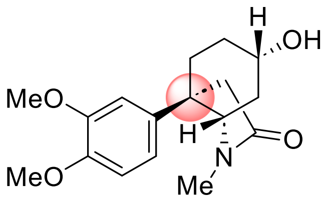

(–)-2-oxo-epimesembranol (**1d**)

(3a*S*,6*S*,7a*S*)-3a-(3,4-Dimethoxyphenyl)-6-hydroxy-1-methyloctahydro-2H-indol-2-one [(–)-1d]: The compound **1d** was obtained as colorless sticky gel (0.330 mmol; 98.65 mg, 98%). R_f = 0.10 (EtOAc only).

^{1}H NMR (400 MHz, Chloroform-*d*): δ 6.87 (dd, *J* = 8.5, 2.2 Hz, 1H), 6.84 (s, 1H), 6.82 (d, *J* = 2.5 Hz, 1H), 3.98 – 3.91 (m, 2H), 3.89 (s, 3H), 3.88 (s, 3H), 2.91 (s, 3H), 2.65 (d, *J* = 16.3

Hz, 1H), 2.54 (d, J = 16.2 Hz, 1H), 2.21 – 2.10 (m, 2H), 1.97 – 1.91 (m, 1H), 1.89 – 1.81 (m, 1H), 1.79 – 1.70 (m, 1H), 1.67 – 1.57 (m, 1H).

^{13}C{^{1}H} NMR (100 MHz, Chloroform-*d*): δ 173.6, 149.1, 147.9, 137.4, 118.1, 111.1, 109.9, 65.8, 62.2, 56.1, 55.9, 45.3, 42.8, 32.8, 30.0, 29.9, 27.9.

FTIR (thin film, neat, cm^{-1}): 3384, 2934, 2862, 1664, 1589, 1519, 1442, 1399, 1333, 1249, 1147, 1100, 1067, 1024, 995, 806, 767, 731.

HRMS (ESI, m/z): calculated for $C_{17}H_{23}NO_4Na$ [M+Na]$^+$: 306.1715, found 306.1700.
$[\alpha]_D^{23.5}$ = –13.66 (c = 0.15, $CHCl_3$)

Total synthesis of (–)-6-epimesembranol [**1b**]

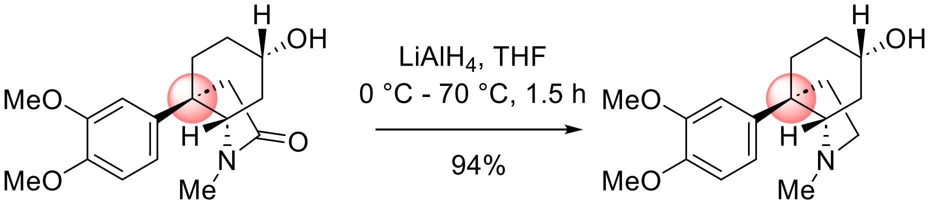

(–)-2-oxo-epimesembranol (**1d**) (–)-6-*epi*-mesembranol (**1b**)

To a solution of **1d** (50 mg, 0.164 mmol, 1 equiv) in dry THF at 0 °C, LAH (31.06 mg, 0.818 mmol, 5 equiv) was added portion wise then the reaction mixture was transferred to refluxing condition at 70 °C and stirred for 1.5 h. The reaction was monitored by TLC. Upon completion, the reaction was quenched by slow addition of aqueous NaOH (1 N), and extracted with EtOAc. The combined organic phase was dried over anhydrous Na_2SO_4 and concentrated. The residue was purified by flash column chromatography (basic alumina, 50% EA/Hexane) to give pure (–)-6-*epi*-mesembranol **1b** (44.89 mg, 94%).

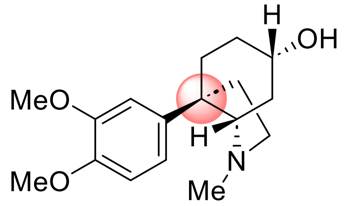

(–)-6-*epi*-mesembranol (**1b**)

(3a*S*,6*S*,7a*S*)-3a-(3,4-Dimethoxyphenyl)-1-methyloctahydro-1*H*-indol-6-ol [(–)-1b]: The compound **1b** was obtained as colorless sticky gel (0.164 mmol; 44.89 mg, 94%). R_f = 0.01 (EtOAc only).

^{1}H NMR (400 MHz, Chloroform-*d*): δ 6.91 (dd, *J* = 10.7, 2.4 Hz, 2H), 6.83 (d, *J* = 8.2 Hz, 1H), 3.95 (s, 1H), 3.90 (s, 3H), 3.89 (s, 3H), 3.40 (td, *J* = 9.4, 6.6 Hz, 1H), 2.92 (d, *J* = 3.0 Hz, 1H), 2.50 (s, 3H), 2.40 (dt, *J* = 10.6, 5.2 Hz, 1H), 2.33 (td, *J* = 14.3, 3.5 Hz, 1H), 2.18 (d, *J* = 15.0, 2.8 Hz, 1H), 1.99 – 1.91 (m, 2H), 1.86 (td, *J* = 12.0, 6.7 Hz, 1H), 1.77 – 1.70 (m, 1H), 1.66 (dt, *J* = 14.9, 3.1 Hz, 1H), 1.48 – 1.38 (m, 1H).

^{13}C{^{1}H} NMR (101 MHz, Chloroform-*d*): δ 148.8, 147.3, 138.2, 118.5, 110.8, 110.4, 68.9, 67.0, 56.0, 55.9, 53.3, 47.5, 41.3, 40.9, 29.8, 28.2, 28.1.

FTIR (thin film, neat, cm^{-1}): 3335, 2934, 2836, 1588, 1519, 1459, 1409, 1253, 1180, 1147, 1095, 1056, 1026, 920, 806, 765.

HRMS (ESI, m/z): calculated for $C_{17}H_{25}NO_3Na$ $[M+Na]^+$: 292.1903, found 292.1907.

$[\alpha]_D^{23}$ = –3.06 (c = 0.31, $CHCl_3$)

Total synthesis of (–)-mesembrine [1a]

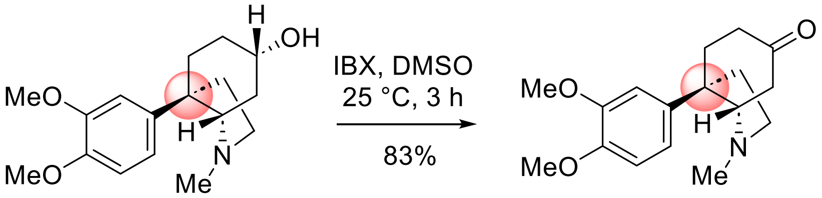

(–)-6-*epi*-mesembranol (**1b**) (–)-mesembrine (**1a**)

To a solution of **1b** (30 mg, 0.103 mmol, 1 equiv) in DMSO at 25 °C, IBX (43.24 mg, 0.154 mmol, 1.5 equiv) was added portion wise then the reaction mixture was stirred for 1.5 h. The reaction was monitored by TLC. Upon completion, the reaction was quenched by water, and extracted with EtOAc. The combined organic phase was dried over anhydrous Na_2SO_4 and concentrated. The residue was purified by flash column chromatography (basic alumina, EA) to give pure **1a** (24.71 mg, 83%).

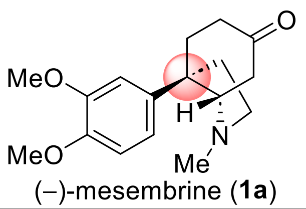

(–)-mesembrine (**1a**)

(3a*S*,7a*S*)-3a-(3,4-Dimethoxyphenyl)-1-methyloctahydro-6*H*-indol-6-one [(–)-1a]: The compound **1a** was obtained as colorless sticky gel (0.103 mmol; 24.71 mg, 83%). R_f = 0.50 (10% MeOH/EtOAc).

^{1}H NMR (400 MHz, Chloroform-*d*): δ 6.96 – 6.91 (m, 2H), 6.86 (d, *J* = 8.3 Hz, 1H), 3.92 (s, 3H), 3.90 (s, 3H), 3.16 (ddd, *J* = 9.3, 7.7, 3.0 Hz, 1H), 2.98 (q, *J* = 3.7 Hz, 1H), 2.62 (dd, *J* = 3.8, 1.5 Hz, 2H), 2.51 – 2.41 (m, 1H), 2.34 (s, 4H), 2.27 – 2.18 (m, 3H), 2.17 – 2.06 (m, 2H).

^{13}C{^{1}H} NMR (100 MHz, Chloroform-*d*): δ 211.4, 149.1, 147.6, 140.2, 118.0, 111.0, 110.0, 70.4, 56.0, 55.9, 54.9, 47.6, 40.6, 40.1, 38.9, 36.2, 35.3.

FTIR (thin film, neat, cm^{-1}): 2941, 2883, 2779, 1715, 1588, 1518, 1452, 1409, 1318, 1251, 1224, 1175, 1146, 1025, 805, 766.

HRMS (ESI, m/z): calculated for $C_{17}H_{23}NO_3Na$ $[M+Na]^+$: 290.1752, found 290.1751.
$[\alpha]_D^{23.5}$ = –87.58 (c = 0.31, $CHCl_3$)

Ester aminolysis of (–)-7b followed by *aza*-Michael reaction/cyclization:

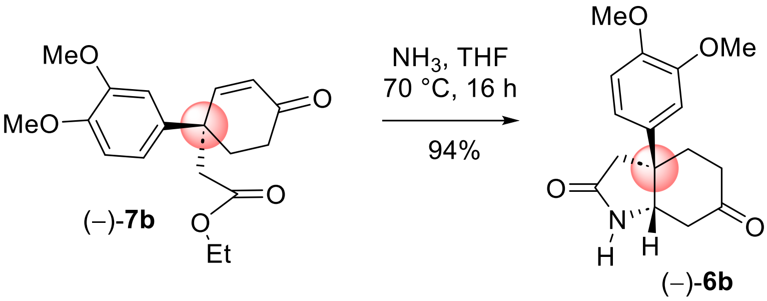

Compound **7b** (1 gm, 3.141 mmol, 1 equiv) was dissolved with 10 ml THF then NH_3 (10 ml, 28-30% in aqueous, 55 equiv), then the resulting mixture was heated to 70 °C in a sealed tube and left stirring for 16 h, at that temperature. The reaction was monitored by TLC. Upon completion the reaction then it was extracted with EtOAc. The combined organic phase was dried over anhydrous Na_2SO_4 and concentrated. The residue was purified by flash column chromatography (silica 100-200, 5% MeOH/EA) to give pure **6b** (853.99 mg, 94%).

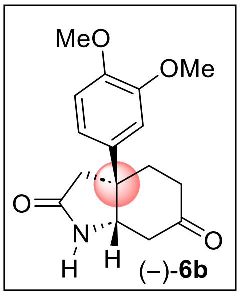

(3a*S*,7a*S*)-3a-(3,4-Dimethoxyphenyl)hexahydro-1*H*-indole-2,6-dione [(–)6b]: The compound **6b** was obtained as white foam (3.141 mmol; 853.99 mg, 94%). R_f = 0.1 (EtOAc only).

^{1}H NMR of 6b (400 MHz, $CDCl_3$): δ: 6.88 (s, 2H), 6.81 (s, 1H), 6.78 (s, 1H), 4.59 (t, 1H), 3.91 (s, 3H), 3.90 (s, 3H), 2.89 – 2.80 (m, 2H), 2.69 – 2.62 (m, 2H), 2.34 (dq, *J* = 10.2, 6.5, 5.4 Hz, 4H).

^{13}C{^{1}H} NMR of 6b (100 MHz, $CDCl_3$): δ: 208.9, 175.6, 149.5, 148.2, 137.4, 117.7, 111.3, 109.4, 58.9, 56.1, 56.0, 46.6, 44.1, 42.8, 36.2, 34.6.

FTIR (thin film, neat, cm^{-1}): 3235, 2936, 2837, 1687, 1589, 1518, 1462, 1411, 1340, 1250, 1146, 1022, 807, 766, 731.

HRMS (ESI-TOF) *m/z*: $[M + H]^+$ Calcd for $[C_{16}H_{19}NO_4 + H]^+$ 290.1377; Found 290.1387.
$[\alpha]_D{}^{21}$ = –35.58 (c = 0.69, $CHCl_3$) R-CBS

Ethylene glycol protection of compound (–)-6b:

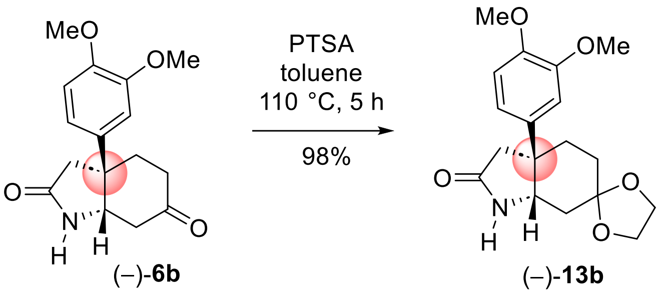

To a solution of **6b** (1 gm, 3.456 mmol, 1.0 equiv.) in toluene, ethylene glycol (961.89 µl, 17.28 mmol, 5.0 equiv) and p-toluenesulfonic acid monohydrate (65.744 mg, 0.346 mmol, 0.10 equiv) was added at RT then the reaction mixture was transferred to refluxing condition at 110 °C and stirred for 5 h. The reaction was monitored by TLC. Upon completion, the reaction was quenched by slow addition of aqueous $NaHCO_3$, and extracted with EtOAc. The combined organic phase was dried over anhydrous Na_2SO_4 and concentrated. The residue was purified by flash column chromatography (100-200, 5% MeOH/EA) to give pure **13b** (1.126 gm, 98%).

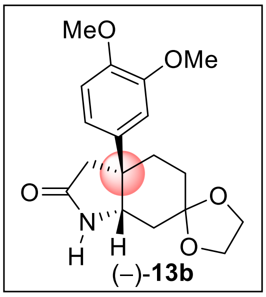

(3a*S*,7a*S*)-3a-(3,4-Dimethoxyphenyl)hexahydrospiro[indole-6,2'-[1,3]dioxolan]-2(1*H*)-one [(–)13b]: The compound **13b** was obtained as white solide (mp: 158-160 ˚C) (3.456 mmol scale of reaction; 1.126 gm, 98%). R_f = 0.1 (EtOAc only).

^{1}H NMR (400 MHz, $CDCl_3$): δ 6.88 (d, *J* = 10.4 Hz, 1H), 6.83 (d, *J* = 7.9 Hz, 2H), 6.51 (s, 1H), 4.31 (t, *J* = 4.3 Hz, 1H), 4.01 – 3.91 (m, 4H), 3.89 (s, 3H), 3.87 (s, 3H), 2.60 (d, *J* = 16.2 Hz, 1H), 2.51 (d, *J* = 16.1 Hz, 1H), 2.20 (t, *J* = 14.8 Hz, 1H), 2.11 – 1.92 (m, 3H), 1.65 – 1.49 (m, 2H).

^{13}**C{^{1}H} NMR** (100 MHz, $CDCl_3$): δ 175.7, 149.0, 147.9, 136.4, 118.3, 111.0, 110.0, 107.3, 64.6, 64.0, 57.8, 56.0, 55.9, 46.7, 45.0, 35.6, 32.4, 30.4.

FTIR (thin film, neat, cm^{-1}): 3224, 2934, 1692, 1515, 1459, 1411, 1248, 1144, 1126, 1092, 1024, 886, 730, 700.

HRMS (ESI-TOF) *m/z*: $[M + H]^+$ Calcd for $[C_{18}H_{23}NO_5 + H]^+$ 334.1631; Found 334.1649.
$[\alpha]_D^{22.5}$ = –41.69 (c = 0.53, $CHCl_3$) *R*-CBS

Amide reduction of [(–)-13b]:

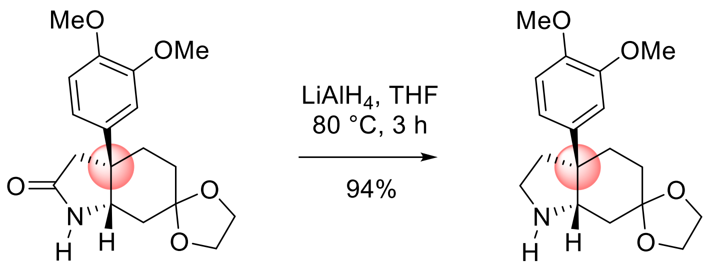

(–)-**13b** (–)-**14b**

To a solution of **13b** (200 mg, 0.599 mmol, 1 equiv) in dry THF at 0 °C, LAH (227.66 mg, 5.999 mmol, 10 equiv) was added portion wise then the reaction mixture was transferred to refluxing condition at 80 °C and stirred for 3 h. The reaction was monitored by TLC. Upon completion, the reaction was quenched by slow addition of aqueous NaOH (1 N), and extracted with EtOAc. The combined organic phase was dried over anhydrous Na_2SO_4 and concentrated. The residue was purified by flash column chromatography (100-200 silica, 5% Et_2NH/EA) to give pure compound **14b** (179.84 mg, 94%).

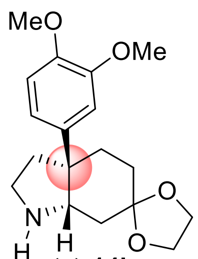

(–)-**14b**

(3a*S*,7a*S*)-3a-(3,4-Dimethoxyphenyl)octahydrospiro[indole-6,2'-[1,3]dioxolane] [(–)-14b]: The compound **14b** was obtained as colour less gel (0.599 mmol; 179.84 mg, 94%). R_f = 0.1 (10% MeOH/EtOAc).

^{1}H NMR of 14b (400 MHz, $CDCl_3$): δ 7.02 – 6.74 (m, 3H), 3.93 (ddd, *J* = 14.5, 11.1, 7.4 Hz, 4H), 3.87 (s, 3H), 3.85 (s, 3H), 3.56 (s, 1H), 3.20 – 3.09 (m, 1H), 2.99 – 2.85 (m, 2H), 2.47 – 2.27 (m, 1H), 2.12 (td, 1H), 2.05 – 1.78 (m, 4H), 1.57 – 1.47 (m, 1H), 1.46 – 1.19 (m, 1H).

^{13}C{^{1}H} NMR of 14b (100 MHz, $CDCl_3$): δ 153.3, 148.8, 148.4, 147.2, 139.0, 138.0, 122.9, 119.3, 118.5, 111.0, 110.9, 110.4, 108.8, 108.7, 64.5, 64.4, 63.8, 63.9, 62.5, 61.8, 60.4, 56.0, 55.9, 55.6, 47.4, 46.8, 43.3, 43.2, 42.6, 40.8, 34.0, 33.6, 31.7, 31.3, 30.6, 29.3.

FTIR (thin film, neat, cm^{-1}): 3334, 2938, 2878, 2834, 1579, 1517, 1461, 1424, 1249, 1125, 1093, 1026, 947, 806, 732.

HRMS (ESI-TOF) *m/z*: $[M + H]^+$ Calcd for $[C_{18}H_{25}NO_4 + H]^+$ 320.1841; Found 320.1856.
$[\alpha]_D^{17.8}$ = –18.05 (c = 1, $CHCl_3$) *R*-CBS

Pictet-Spengler cyclization of [(–)-14b]:

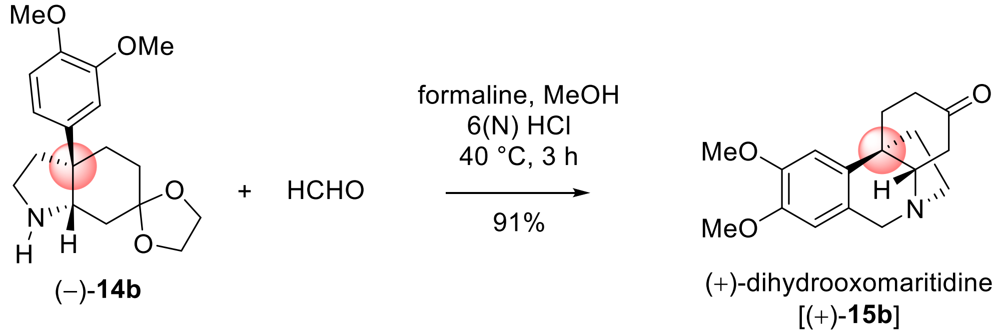

To a solution of **14b** (50 mg, 0.156 mmol, 1 equiv) in MeOH at 0 °C, 37% formaldehyde (470.11 µl, 15.65 mmol, 100 equiv) was added then the reaction mixture was stirred for 10 min, after that 6 (*N*) HCl (13.04 ml, 78.25 mmol, 500 equiv) was added then the reaction mixture was warmed to 40 °C and stirred for 3 h. The reaction was quenched with slow addition of solid K_2CO_3 to adjust pH to 10 and extracted with $CHCl_3$ (3 × 20 ml). The combined organic phase was dried over anhydrous Na_2SO_4 and concentrated. The residue was purified

by flash column chromatography (100-200 silica, 2% Et_2NH/EA) to give pure compound **15b** (40.92 mg, 91%).

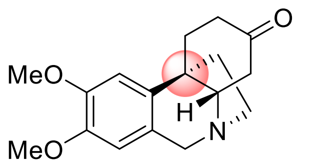
[(+)-**15b**]

(5*R*,10b*S*)-8,9-Dimethoxy-1,2,4,4a-tetrahydro-3*H*,6*H*-5,10*b*-ethanophenanthridin-3-one [(+)-15b]: The compound **15b** was obtained as colour less gel (0.156 mmol; 40.92 mg, 91%). R_f = 0.2 (10% MeOH/EtOAc).

^{1}H NMR of 15b (500 MHz, $CDCl_3$): δ 6.72 (s, 1H), 6.52 (s, 1H), 4.40 (d, *J* = 16.8 Hz, 1H), 3.86 (s, 3H), 3.83 (s, 4H), 3.55 – 3.47 (m, 1H), 3.33 – 3.27 (m, 1H), 3.01 – 2.94 (m, 1H), 2.81 – 2.75 (m, 1H), 2.69 – 2.60 (m, 1H), 2.59 – 2.52 (m, 1H), 2.49 – 2.40 (m, 2H), 2.36 (t, *J* = 14.1, 12.1 Hz, 1H), 2.12 (td, *J* = 13.7, 5.1 Hz, 1H), 2.05 – 1.98 (m, 1H).

^{13}C{^{1}H} NMR of 15b (125 MHz, $CDCl_3$): δ 209.8, 147.9, 147.7, 138.5, 124.6, 109.2, 106.3, 69.2, 61.9, 56.1, 55.9, 52.0, 43.3, 42.2, 37.7, 37.0, 27.5.

FTIR (thin film, neat, cm^{-1}): 2930, 1712, 1607, 1511, 1461, 1307, 1261, 1216, 1147, 1115, 1068, 1035, 762, 732.

HRMS (ESI-TOF) *m/z*: $[M + H]^+$ Calcd for $[C_{17}H_{21}NO_3 + H]^+$ 288.1582; Found 288.1594. $[\alpha]_D^{18.3}$ = +19.50 (c = 0.5, $CHCl_3$)

Syntheses of (+)-dihydromaritidine [2c]

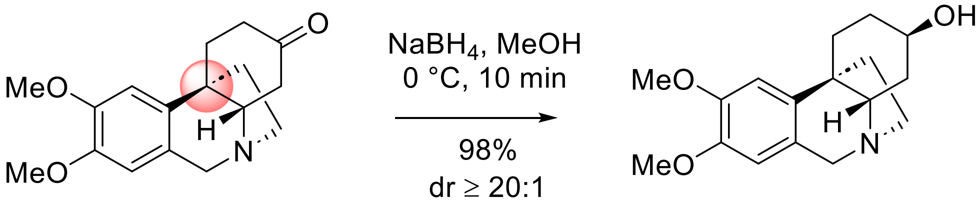

(+)-dihydrooxomaritidine [(+)-**15b**] (+)-dihydromaritidine [(+)-**2c**]

To a solution of **15b** (30 mg, 0.104 mmol, 1 equiv) in MeOH at 0 °C, $NaBH_4$ (4.34 mg, 0.114 mmol, 1.1 equiv) was added portion wise and stirred for 10 min. The reaction was monitored by TLC. Upon completion, the reaction was quenched by water, and evaporated the organic solvent and extracted with EtOAc. The combined organic phase was dried over anhydrous Na_2SO_4 and concentrated. The residue was purified by flash column chromatography (100-200 silica, 5% Et_2NH/EtOAc) to give pure **2c** (29.49 mg, 98%).

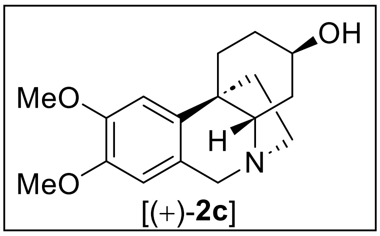

(3*S*,5*R*,10b*S*)-8,9-Dimethoxy-2,3,4,4a-tetrahydro-1*H*,6*H*-5,10*b*-ethanophenanthridin -3-ol [(+)2c]: The compound **2c** was obtained as colour less gel (0.104 mmol; 29.49 mg, 91%). R_f = 0.1 (10% MeOH/EtOAc).

^{1}H NMR (500 MHz, $CDCl_3$): δ 6.72 (s, 1H), 6.51 (s, 1H), 4.40 (d, *J* = 16.5 Hz, 1H), 3.88 (s, 3H), 3.84 (s, 3H), 3.81 (d, *J* = 21.2 Hz, 1H), 3.70 – 3.61 (m, 1H), 3.45 – 3.36 (m, 1H), 2.99 (dd, *J* = 12.3, 5.1 Hz, 1H), 2.90 – 2.80 (m, 1H), 2.47 (d, *J* = 14.5 Hz, 1H), 2.27 – 2.20 (m, 1H), 2.10 – 2.00 (m, 2H), 1.85 – 1.74 (m, 2H), 1.65 – 1.56 (m, 1H), 1.34 (q, 1H).

^{13}C{^{1}H} NMR (125 MHz, $CDCl_3$): δ 147.6, 147.5, 140.0, 124.6, 109.2, 106.3, 69, 66.7, 61.9, 56.1, 55.9, 52.0, 41.8, 37.8, 36.8, 30.8, 26.5.

FTIR (thin film, neat, cm^{-1}): 3346, 2931, 2855, 1635, 1607, 1509, 1462, 1311, 1251, 1217, 1074, 1031, 765, 732.

HRMS (ESI-TOF) *m*/*z*: $[M + H]^+$ Calcd for $[C_{17}H_{23}NO_3 + H]^+$ 290.1750; Found 290.1751.
$[\alpha]_D^{19.6}$ = +5.46 (c = 0.5, $CHCl_3$) using (*R*)-CBS

Spectral Traces

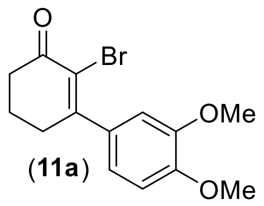

(**11a**)

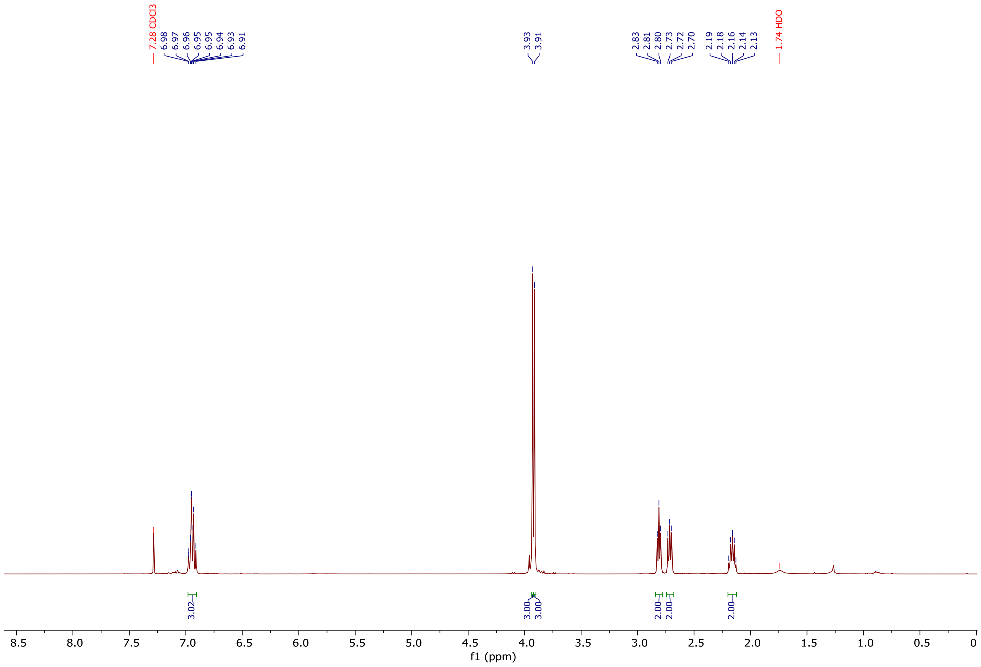

^{1}H NMR (400 MHz, $CDCl_3$) of compound **11a**

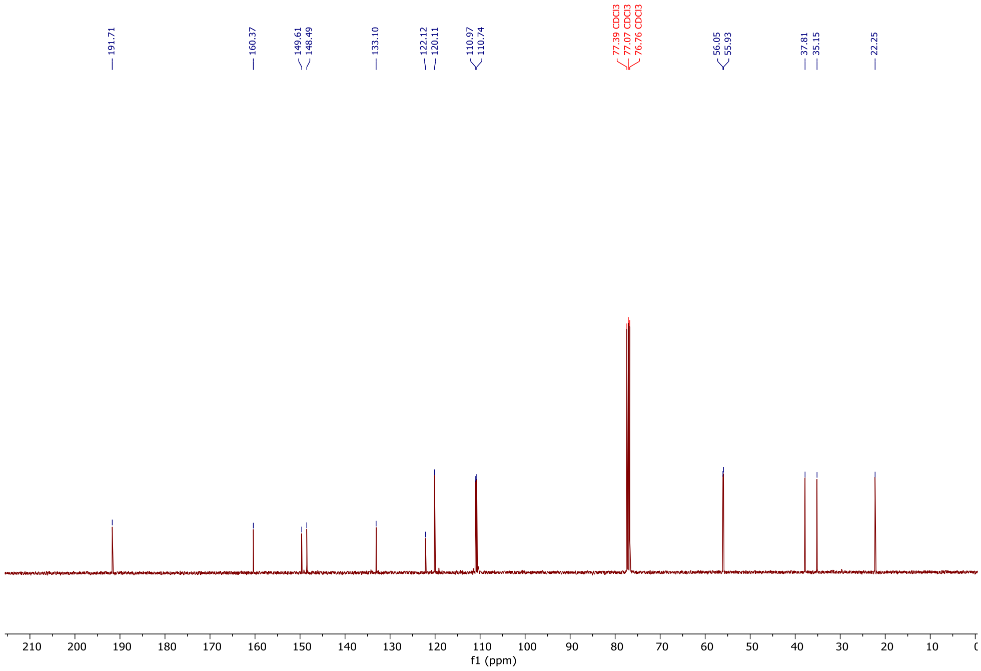

^{13}C{^{1}H} NMR (100 MHz, $CDCl_3$) of compound **11a**

Display Report

Analysis Info

		Acquisition Date	9/17/2021 4:04:39 PM
Analysis Name	D:\Data\new user data 2021\Sept-2021\17-sept\Prof.A.Bisai-ABSM02125_1-B,5_01_9388.d		
Method	hrlcms-20 sept--union-esi-tune low 01-sept-2021.m	Operator	RUCHI
Sample Name	Prof.A.Bisai-ABSM02125	Instrument	micrOTOF-Q II 10330
Comment			

Acquisition Parameter

Source Type	ESI	Ion Polarity	Positive	Set Nebulizer	0.4 Bar
Focus	Not active	Set Capillary	4600 V	Set Dry Heater	180 °C
Scan Begin	50 m/z	Set End Plate Offset	-500 V	Set Dry Gas	4.0 l/min
Scan End	3000 m/z	Set Collision Cell RF	100.0 Vpp	Set Divert Valve	Waste

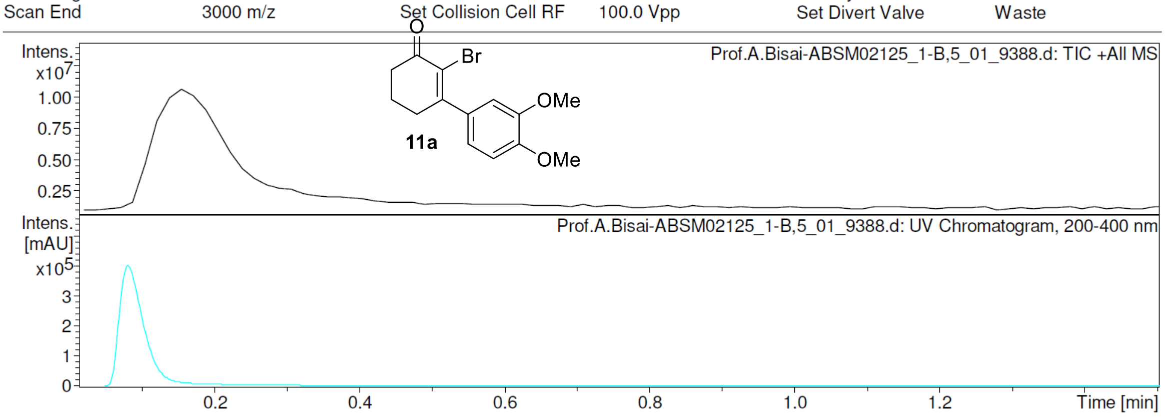

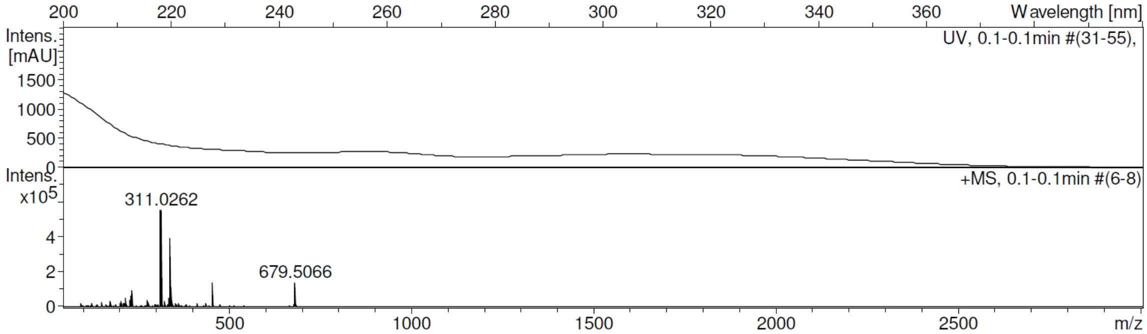

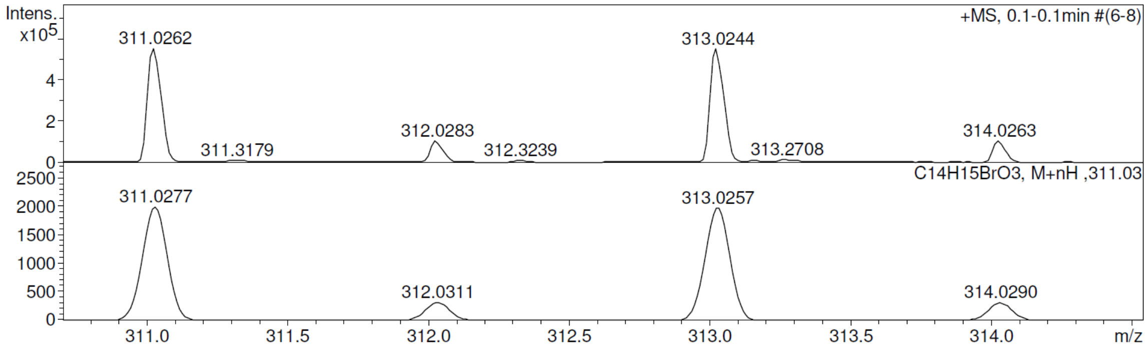

Bruker Compass DataAnalysis 4.0 printed: 9/17/2021 4:09:19 PM Page 1 of 1

HRMS (ESI, m/z): of compound **11a**

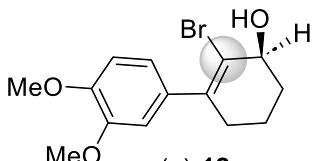

(–)-**12a**

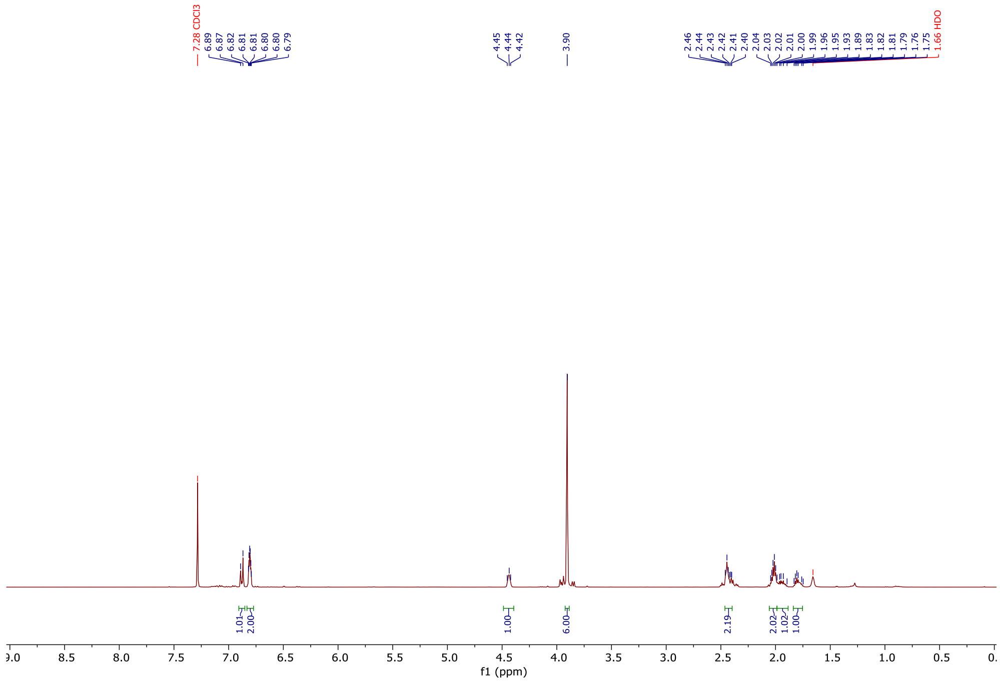

^{1}H NMR (400 MHz, $CDCl_3$) of compound (–)-**12a**

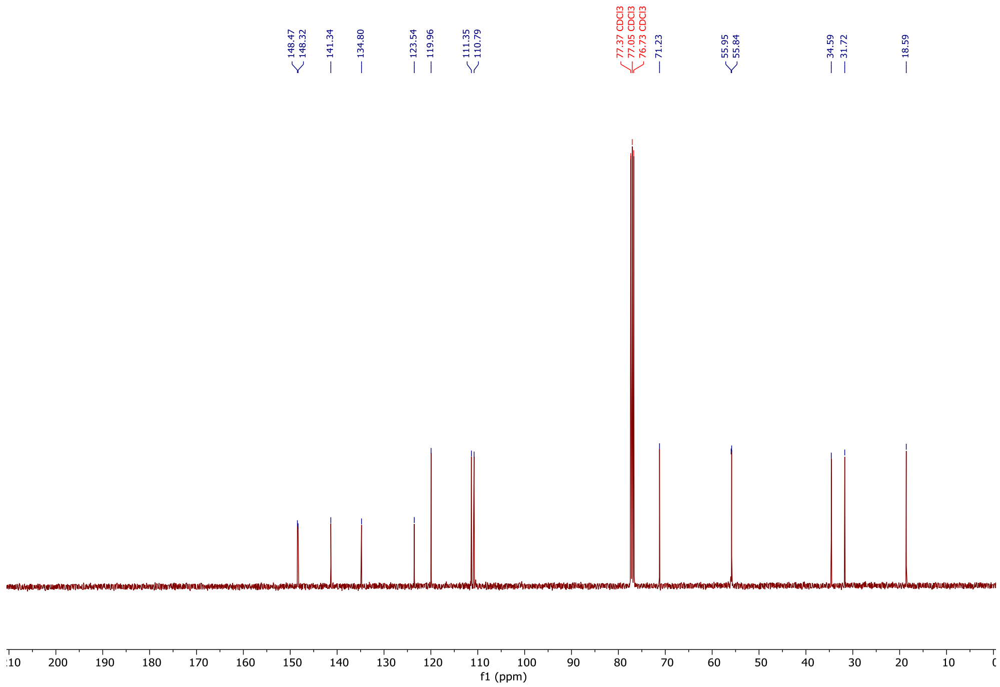

^{13}C{^{1}H} NMR (100 MHz, $CDCl_3$) of compound (–)-**12a**

Display Report

Analysis Info

Analysis Name	D:\Data\new user data 2021\Dec-2021\3-dec\Dr.A.Bisai-AB-SM-02128_1-C,5_01_10385.d	Acquisition Date	12/3/2021 1:48:40 PM
Method	hrlcms-20 sept.m	Operator	RUCHI
Sample Name	Dr.A.Bisai-AB-SM-02128	Instrument	micrOTOF-Q II 10330
Comment			

Acquisition Parameter

Source Type	ESI	Ion Polarity	Positive	Set Nebulizer	1.2 Bar
Focus	Active	Set Capillary	4500 V	Set Dry Heater	200 °C
Scan Begin	50 m/z	Set End Plate Offset	-500 V	Set Dry Gas	6.0 l/min
Scan End	3000 m/z	Set Collision Cell RF	130.0 Vpp	Set Divert Valve	Waste

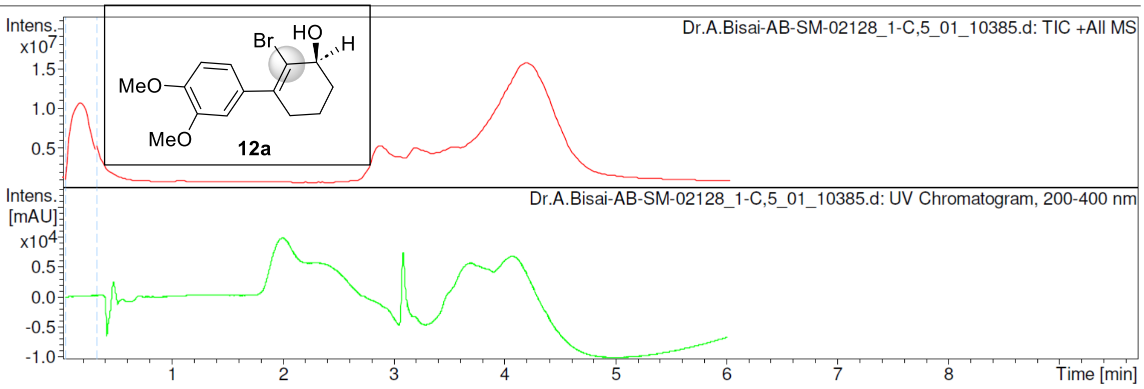

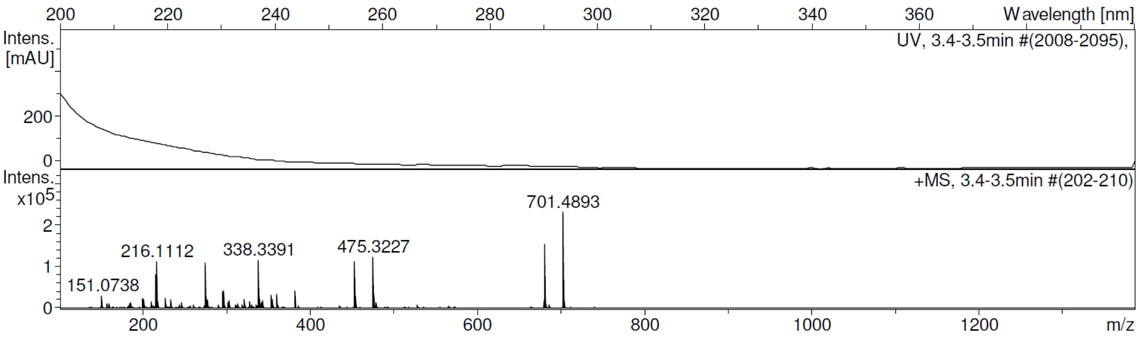

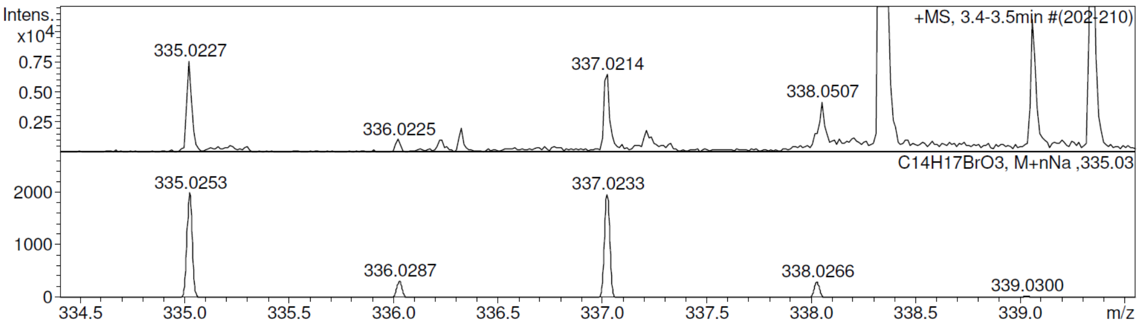

HRMS (ESI, m/z): of compound **12a**

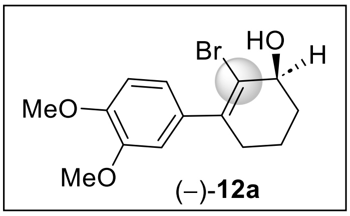

Data File C:\Chem32\2\Data\CK\AB-SM-02-179-RAC-IC-10IPA-1ML.D
Sample Name: AB-SM-02-179-RAC-IC-10IPA-1ML

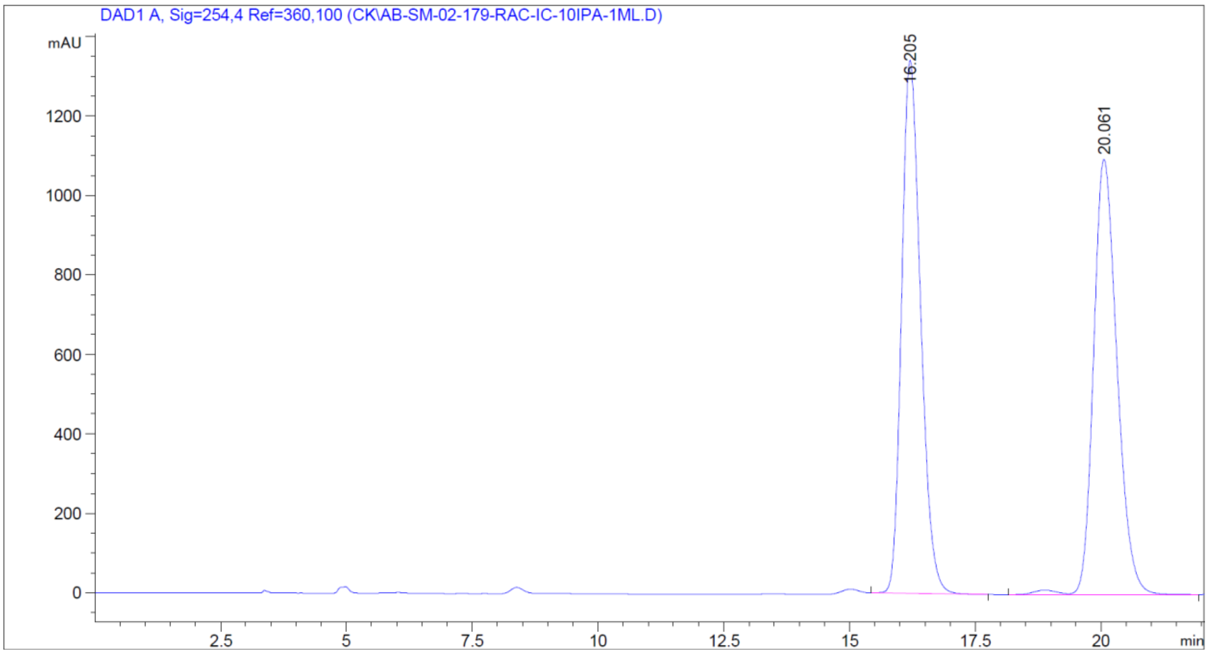

HPLC data of (±)-**12a**

Signal 1: DAD1 A, Sig=254,4 Ref=360,100

Peak #	RetTime [min]	Type	Width [min]	Area [mAU*s]	Height [mAU]	Area %
1	16.205	BB	0.4062	3.52137e4	1341.68372	49.5950
2	20.061	VB R	0.4990	3.57888e4	1095.82056	50.4050

Totals : 7.10026e4 2437.50427

*** End of Report ***

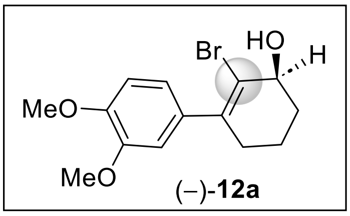

Data File C:\Chem32\2\Data\CK\AB-SM-02-179B-CHI-IC-10IPA-1ML.D
Sample Name: AB-SM-02-179B-CHI-IC-10IPA-1ML

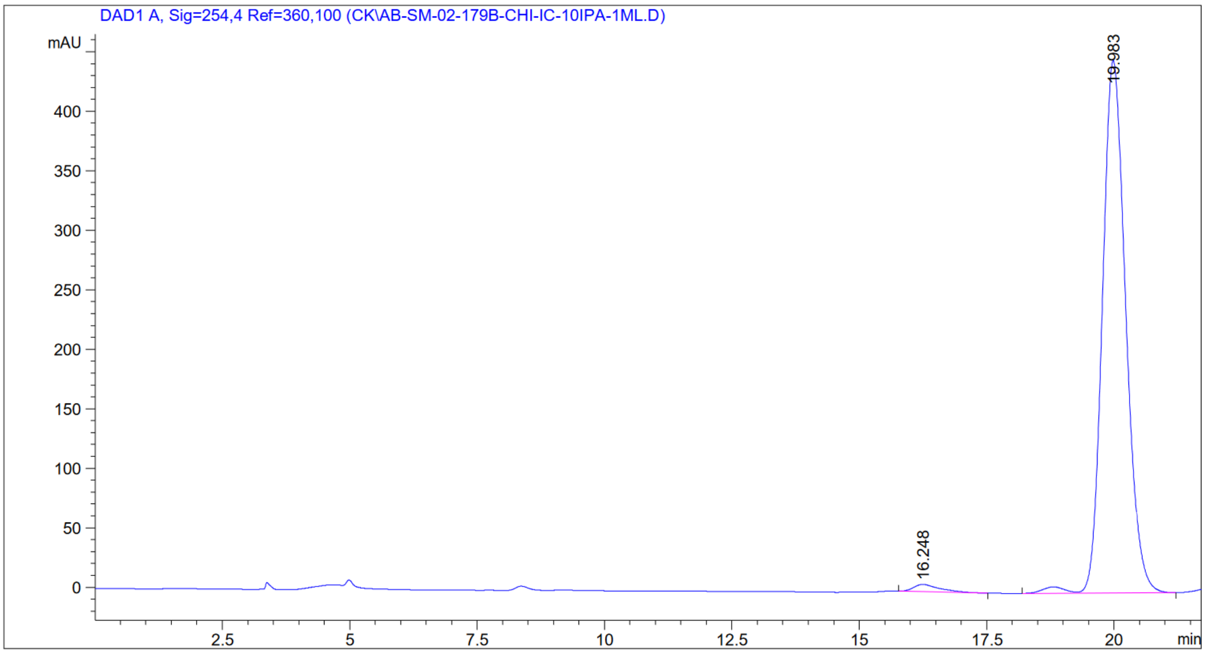

HPLC data of (–)-**12a**

Signal 1: DAD1 A, Sig=254,4 Ref=360,100

Peak #	RetTime [min]	Type	Width [min]	Area [mAU*s]	Height [mAU]	Area %
1	16.248	BB	0.5114	223.44075	6.18554	1.5410
2	19.983	VB R	0.4881	1.42762e4	447.38947	98.4590

Totals : 1.44996e4 453.57500

==

*** End of Report ***

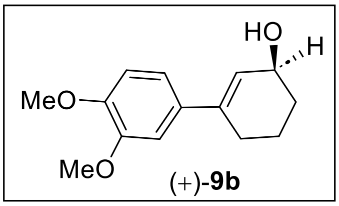

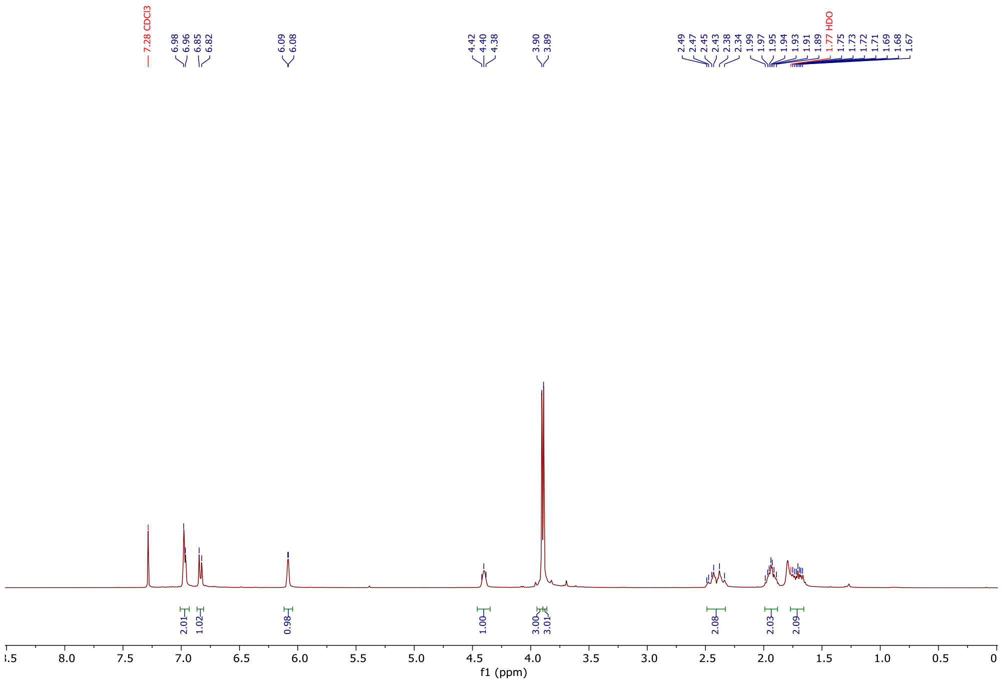

^{1}H NMR (400 MHz, $CDCl_3$) of compound (+)-**9b**

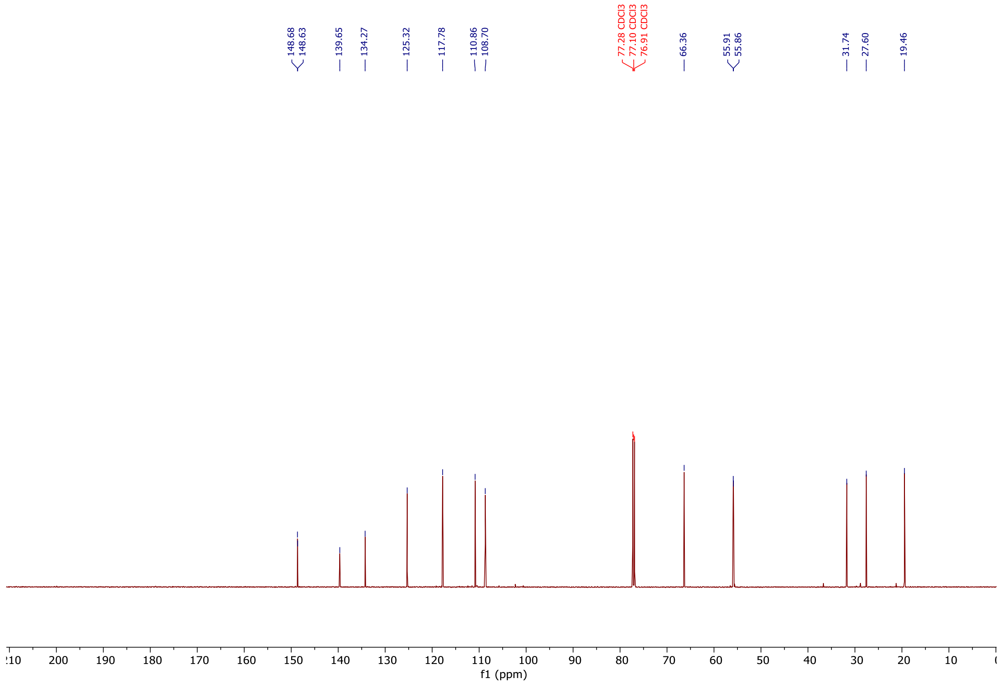

^{13}C{^{1}H} NMR (175 MHz, $CDCl_3$) of compound (+)-**9b**

Display Report

Analysis Info

		Acquisition Date	12/17/2021 3:20:50 PM
Analysis Name	D:\Data\new user data 2021\Dec-2021\17-dec\Prof.A.Bisai-AB-SM-02180_1-C,1_01_10554.d		
Method	hrlcms-20 sept.m	Operator	RUCHI
Sample Name	Prof.A.Bisai-AB-SM-02180	Instrument	micrOTOF-Q II 10330
Comment			

Acquisition Parameter

Source Type	ESI	Ion Polarity	Positive	Set Nebulizer	1.2 Bar
Focus	Active	Set Capillary	4500 V	Set Dry Heater	200 °C
Scan Begin	50 m/z	Set End Plate Offset	-500 V	Set Dry Gas	6.0 l/min
Scan End	3000 m/z	Set Collision Cell RF	130.0 Vpp	Set Divert Valve	Waste

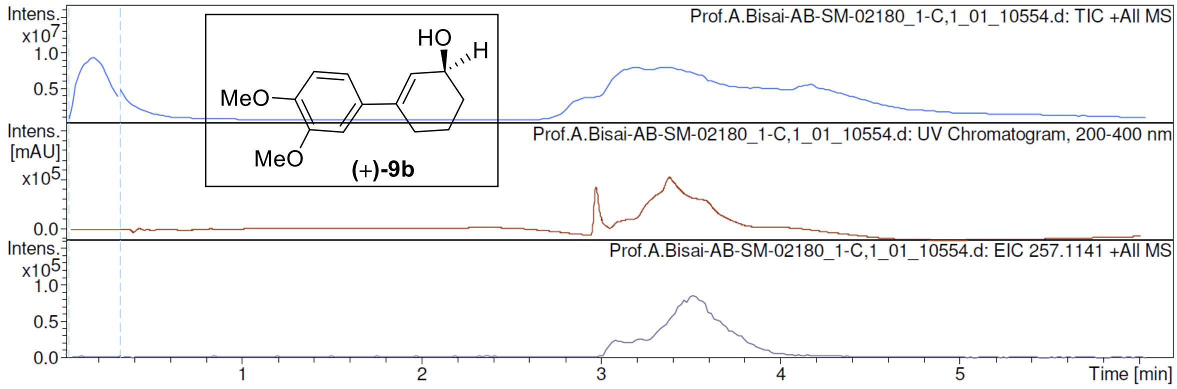

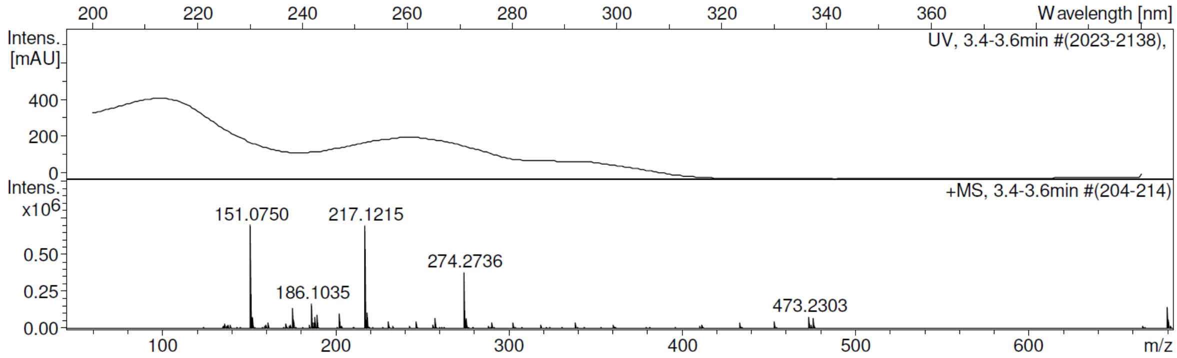

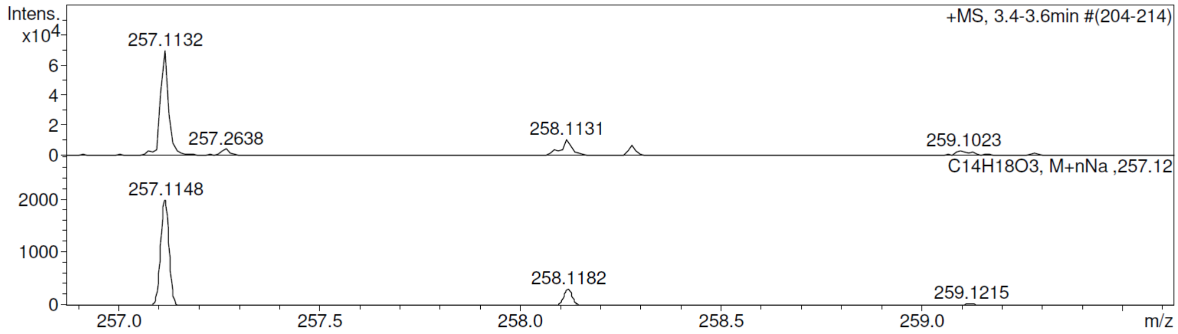

Bruker Compass DataAnalysis 4.0 printed: 12/17/2021 3:36:39 PM Page 1 of 1

HRMS (ESI, m/z): of compound **9b**

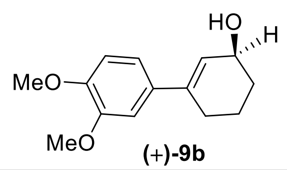

(+)-**9b**

Data File C:\Chem32\2\Data\CK\AB-SM-02-181-RAC-IC-30IPA-1ML.D
Sample Name: AB-SM-02-181-RAC-IC-30IPA-1ML

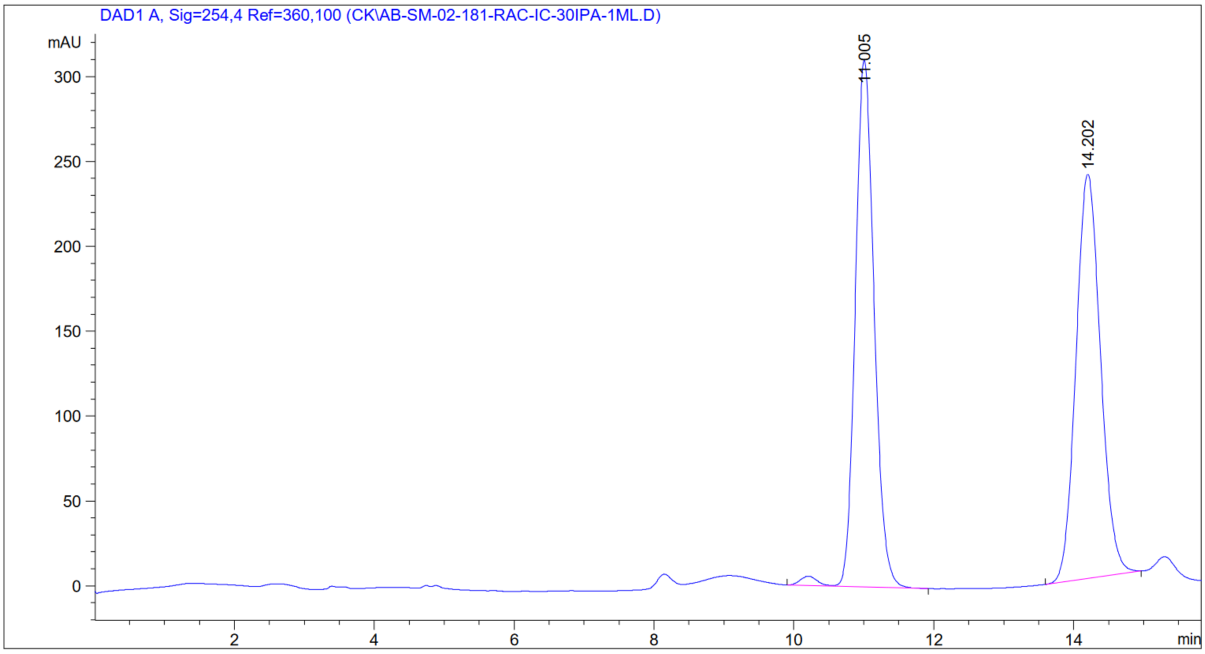

HPLC data of (±)-**9b**

Signal 1: DAD1 A, Sig=254,4 Ref=360,100

Peak #	RetTime [min]	Type	Width [min]	Area [mAU*s]	Height [mAU]	Area %
1	11.005	VB R	0.2875	5878.46436	310.33005	50.2803
2	14.202	BB	0.3787	5812.92773	238.24553	49.7197

Totals : 1.16914e4 548.57558

===

*** End of Report ***

Data File C:\Chem32\2\Data\CK\AB-SM-02-181A-CHI-IC-30IPA-1ML.D
Sample Name: AB-SM-02-181A-CHI-IC-30IPA-1ML

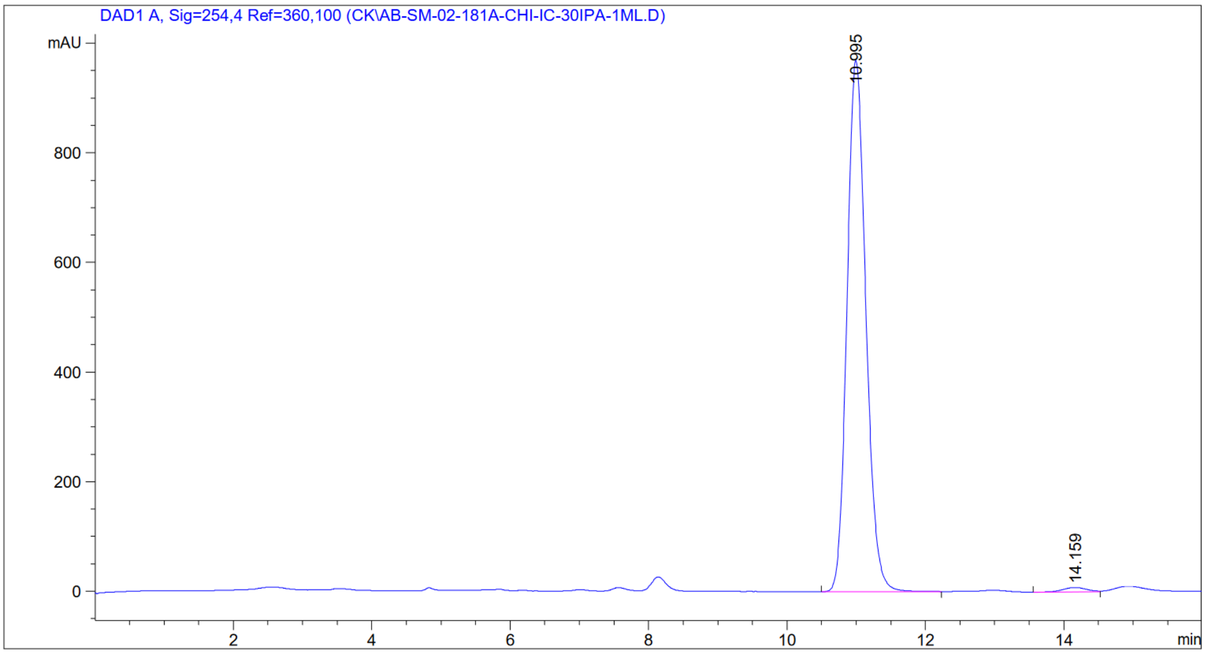

HPLC data of (+)-**9b**

Signal 1: DAD1 A, Sig=254,4 Ref=360,100

Peak #	RetTime [min]	Type	Width [min]	Area [mAU*s]	Height [mAU]	Area %
1	10.995	BB	0.2923	1.83027e4	969.16522	98.8779
2	14.159	BV	0.3814	207.71071	8.31480	1.1221

Totals :	1.85104e4	977.48002

*** End of Report ***

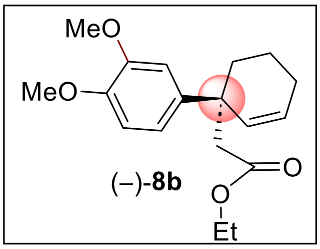

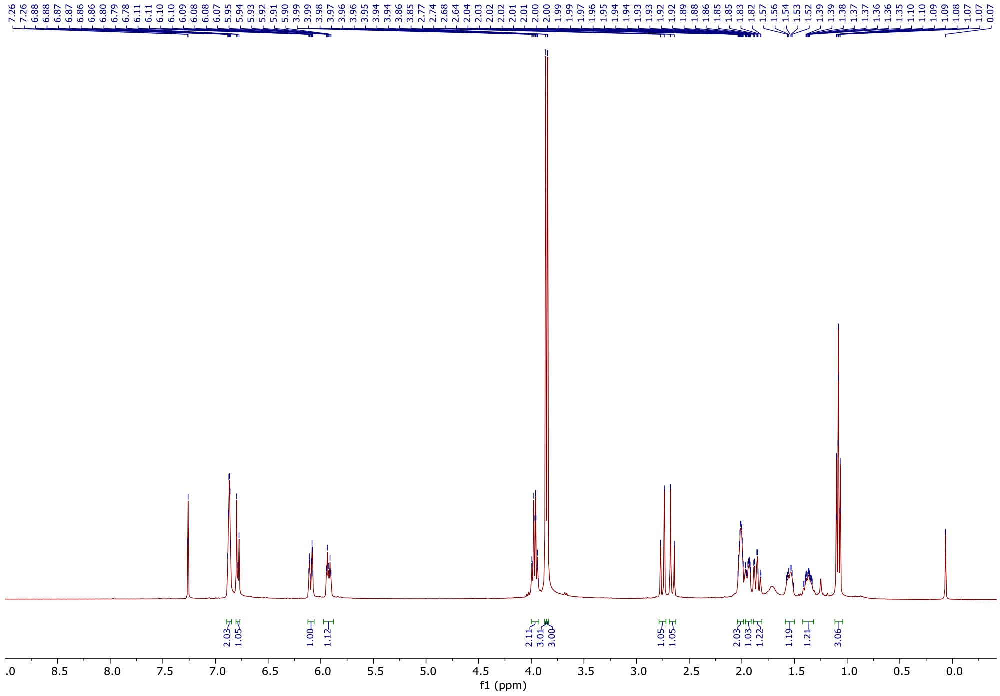

^{1}H NMR (400 MHz, CDCl3) of compound (−)-**8b**

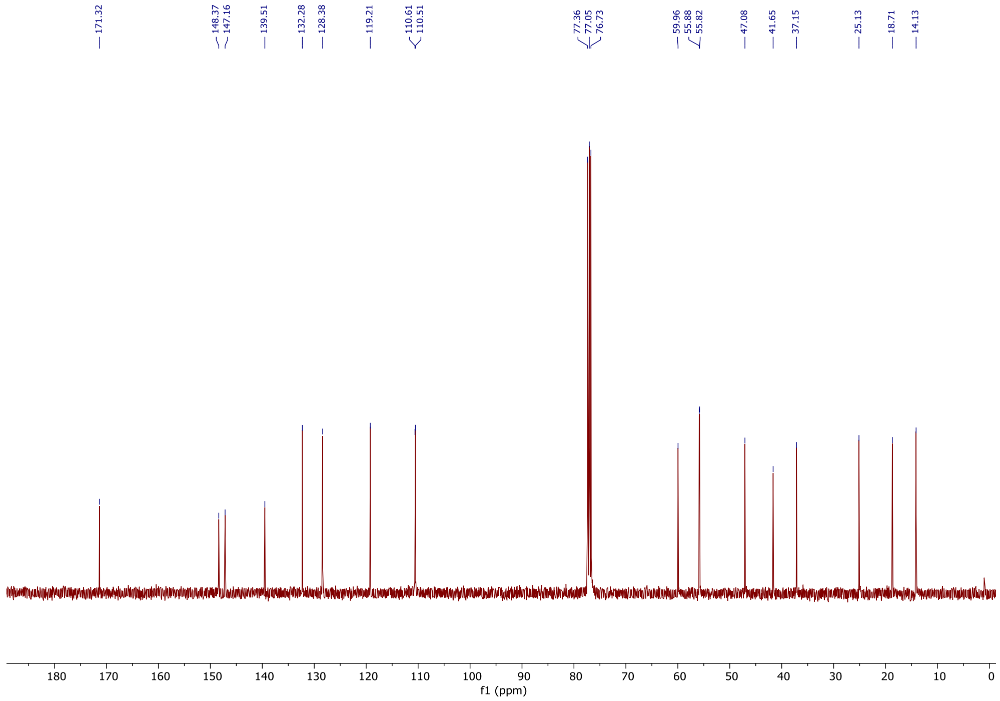

^{13}C{^{1}H} NMR (100 MHz, $CDCl_3$) of compound (−)-**8b**

Display Report

Analysis Info

Analysis Name	D:\Data\new user data 2021\Sept-2021\01Sept\Dr.A.Bisai-AB-SM-02-114_1-A,4_01_8777.d	Acquisition Date	9/1/2021 12:49:32 PM
Method	hrlcms-20 sept--union-small molecules 01-sept-2021.m	Operator	RUCHI
Sample Name	Dr.A.Bisai-AB-SM-02-114	Instrument	micrOTOF-Q II 10330
Comment			

Acquisition Parameter

Source Type	ESI	Ion Polarity	Positive	Set Nebulizer	0.4 Bar
Focus	Not active	Set Capillary	4600 V	Set Dry Heater	180 °C
Scan Begin	50 m/z	Set End Plate Offset	-500 V	Set Dry Gas	4.0 l/min
Scan End	3000 m/z	Set Collision Cell RF	100.0 Vpp	Set Divert Valve	Waste

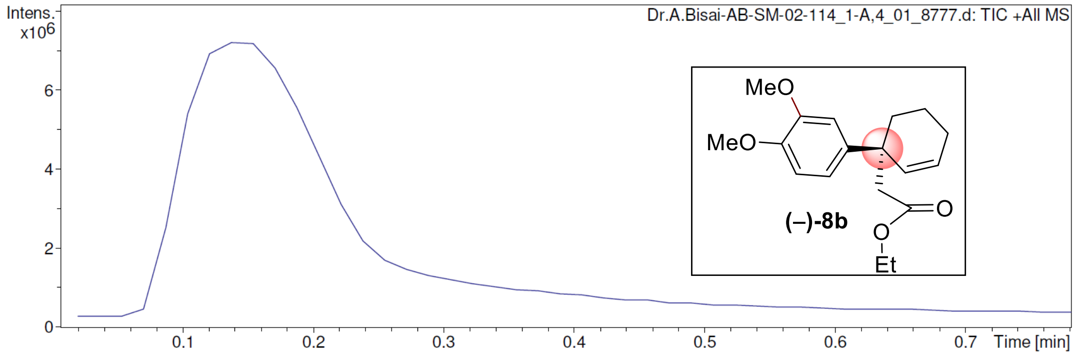

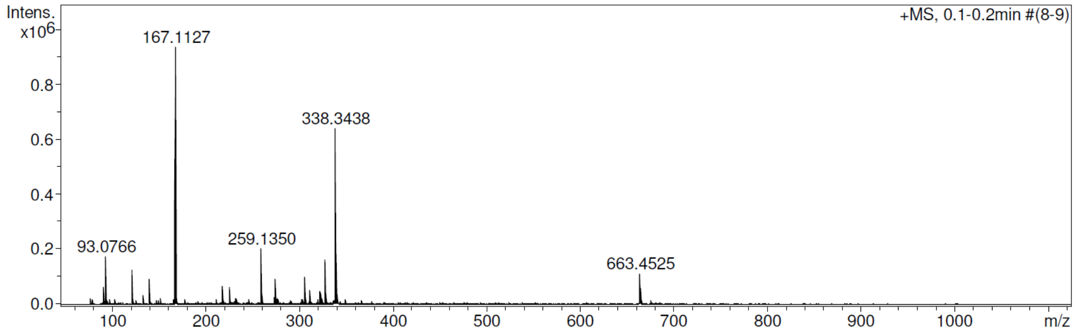

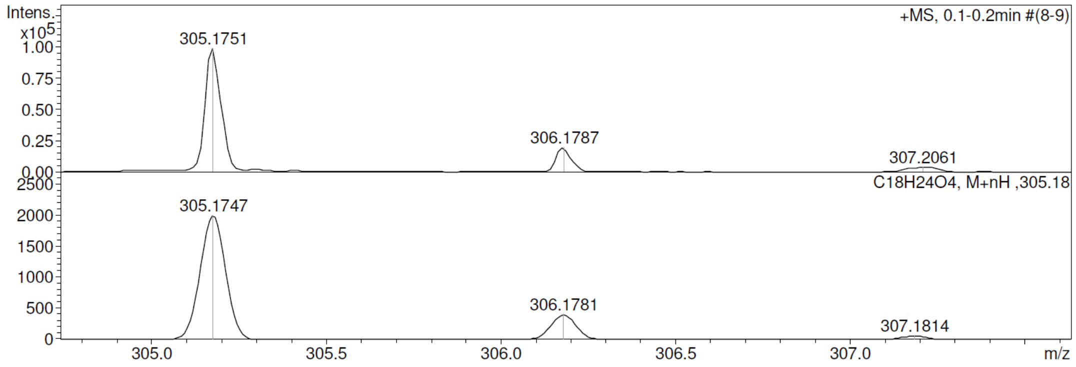

Bruker Compass DataAnalysis 4.0 printed: 9/1/2021 12:50:58 PM Page 1 of 1

HRMS (ESI, m/z): of compound **8b**

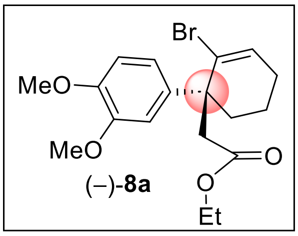

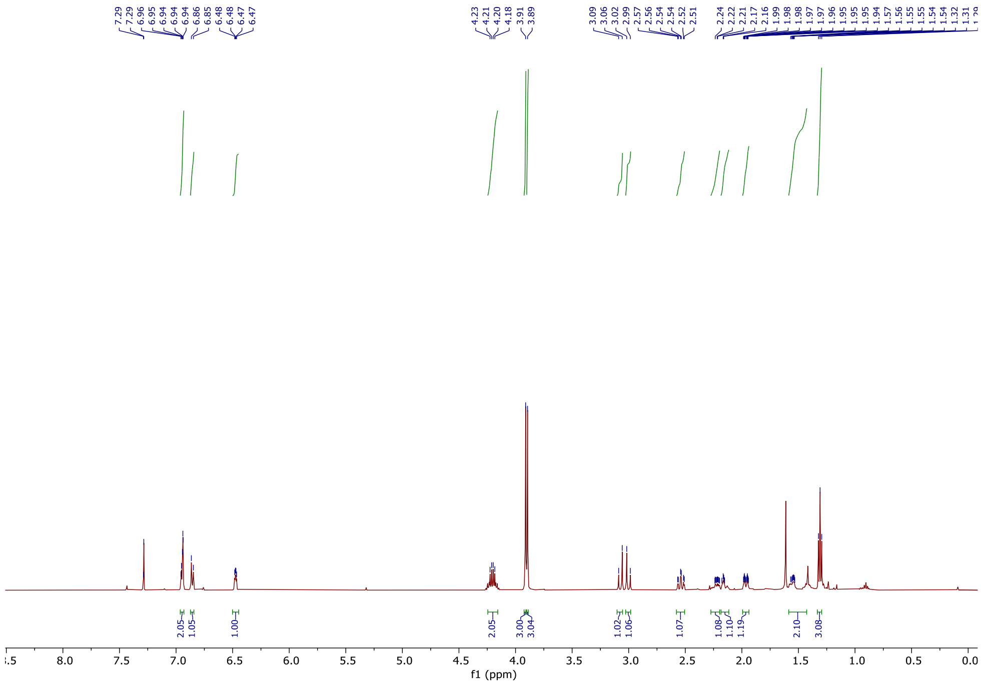

^{1}H NMR (500 MHz, $CDCl_3$) of Compound (–)-**8a**

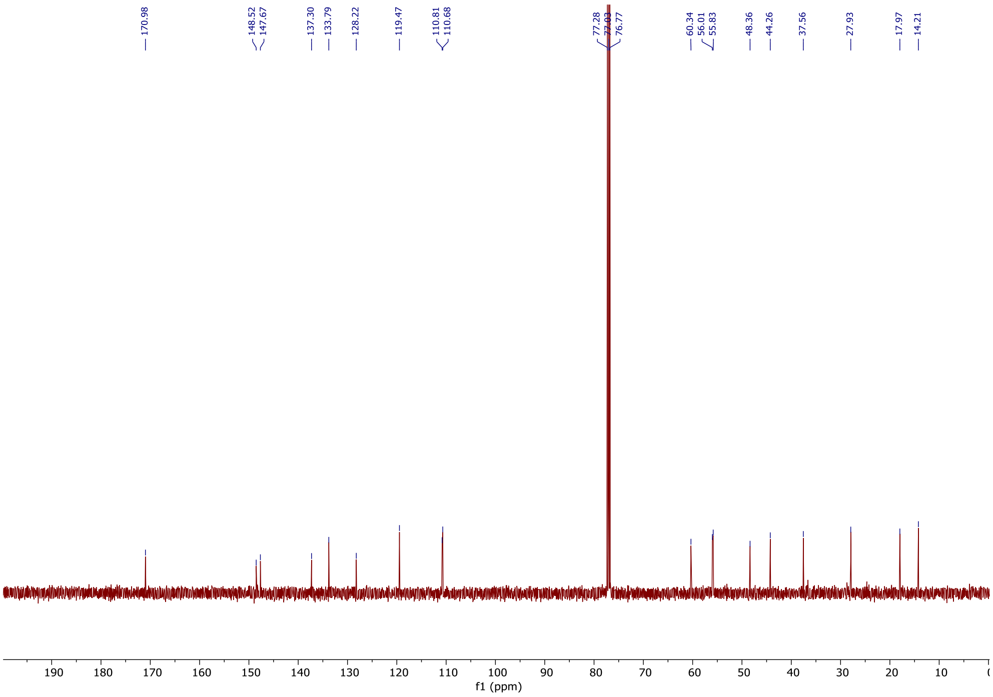

$^{13}C\{^{1}H\}$ NMR (126 MHz, $CDCl_3$) of Compound (–)-**8a**

Display Report

Analysis Info

Analysis Name	D:\Data\User data\2022\JUNE\ab-aya-02022m.d	Acquisition Date	6/7/2022 6:01:20 PM
Method	Tune_pos_Standard.m	Operator	IISER Kolkata
Sample Name	ab-aya-02022m	Instrument	maXis impact 8282001.00127
Comment			

Acquisition Parameter

Source Type	ESI	Ion Polarity	Positive	Set Nebulizer	0.5 Bar
Focus	Active	Set Capillary	3400 V	Set Dry Heater	200 °C
Scan Begin	50 m/z	Set End Plate Offset	-500 V	Set Dry Gas	4.0 l/min
Scan End	1000 m/z	Set Charging Voltage	2000 V	Set Divert Valve	Source
		Set Corona	0 nA	Set APCI Heater	0 °C

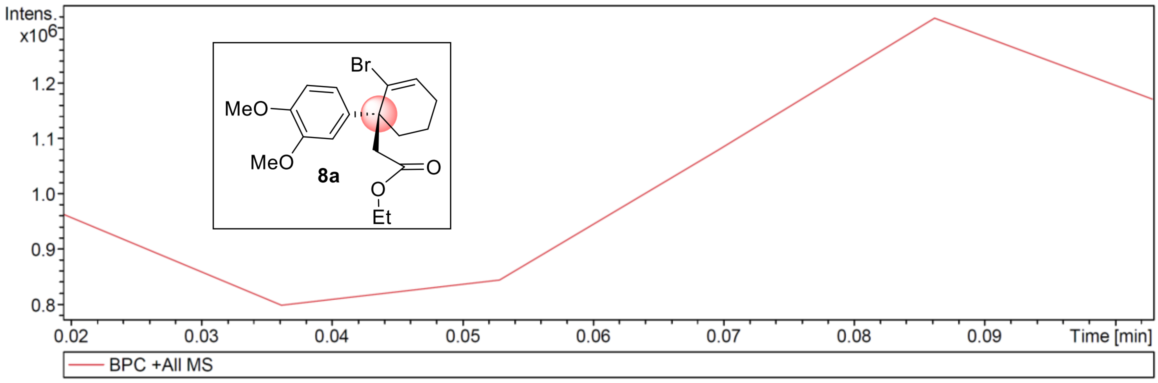

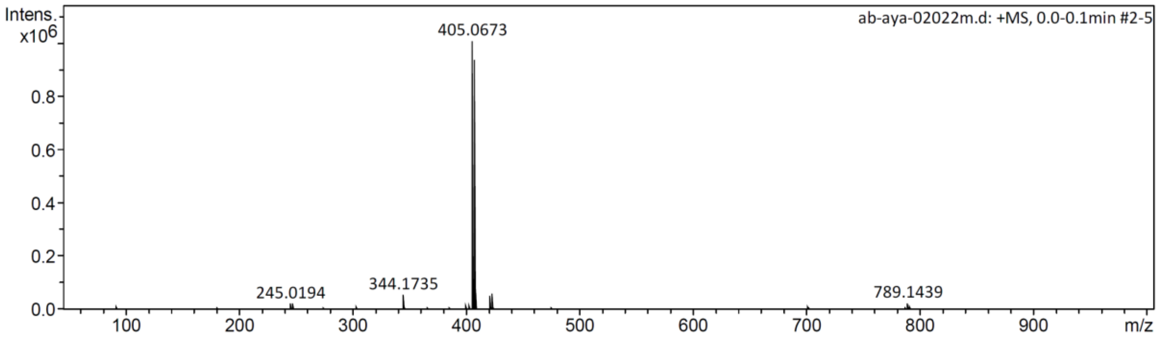

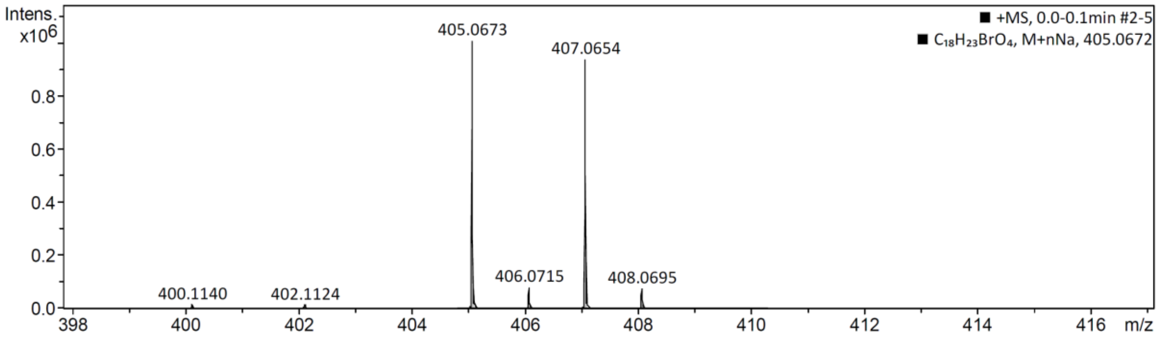

ab-aya-02022m.d

Bruker Compass DataAnalysis 4.1 printed: 6/7/2022 6:03:34 PM by: IISER Kolkata Page 1 of 1

HRMS (ESI, m/z): of compound **8a**

Peak Summary Report

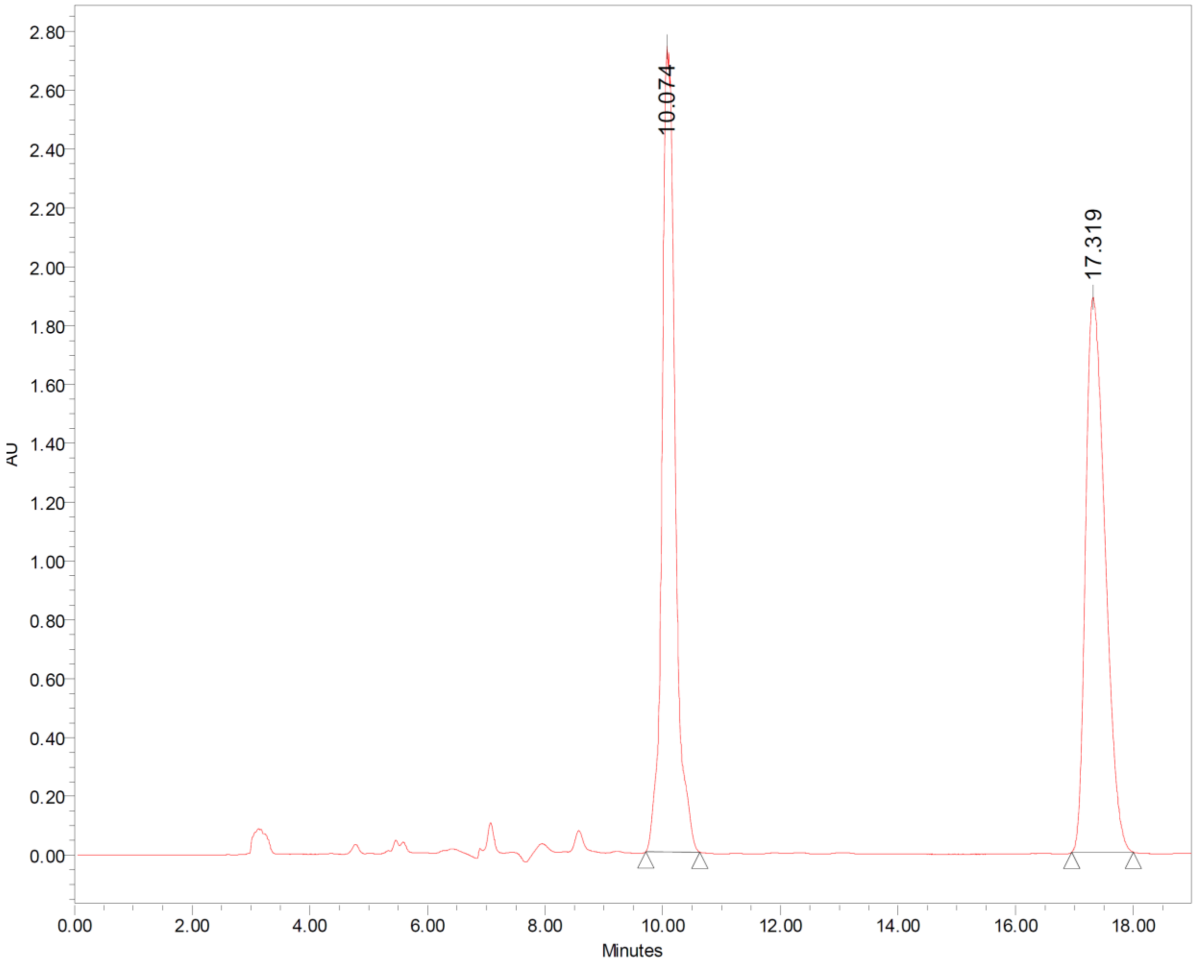

Sample Name: AB_AYA_02022_RAC_IC-3_15_1; Date Acquired: 09-06-2022 14:49:23 IST; Vial: 1 Injection: 1

Peak Summary with Statistics

Name:

	Sample Name	Vial	Inj	Retention Time (min)	Area	% Area	Height
1	AB_AYA_02022_RAC_IC-3_15_1	1	1	17.319	43343914	50.40	1886995
2	AB_AYA_02022_RAC_IC-3_15_1	1	1	10.074	42660336	49.60	2735832
Mean				13.696			
Std. Dev.				5.123			

Reported by User: System
Report Method: Peak Summary Report
Report Method ID 1009
Page: 1 of 2

Project Name: AB Research Group
Date Printed:
09-06-2022
15:13:01 Asia/Calcutta

HPLC data of (±)-**8a**

Peak Summary Report

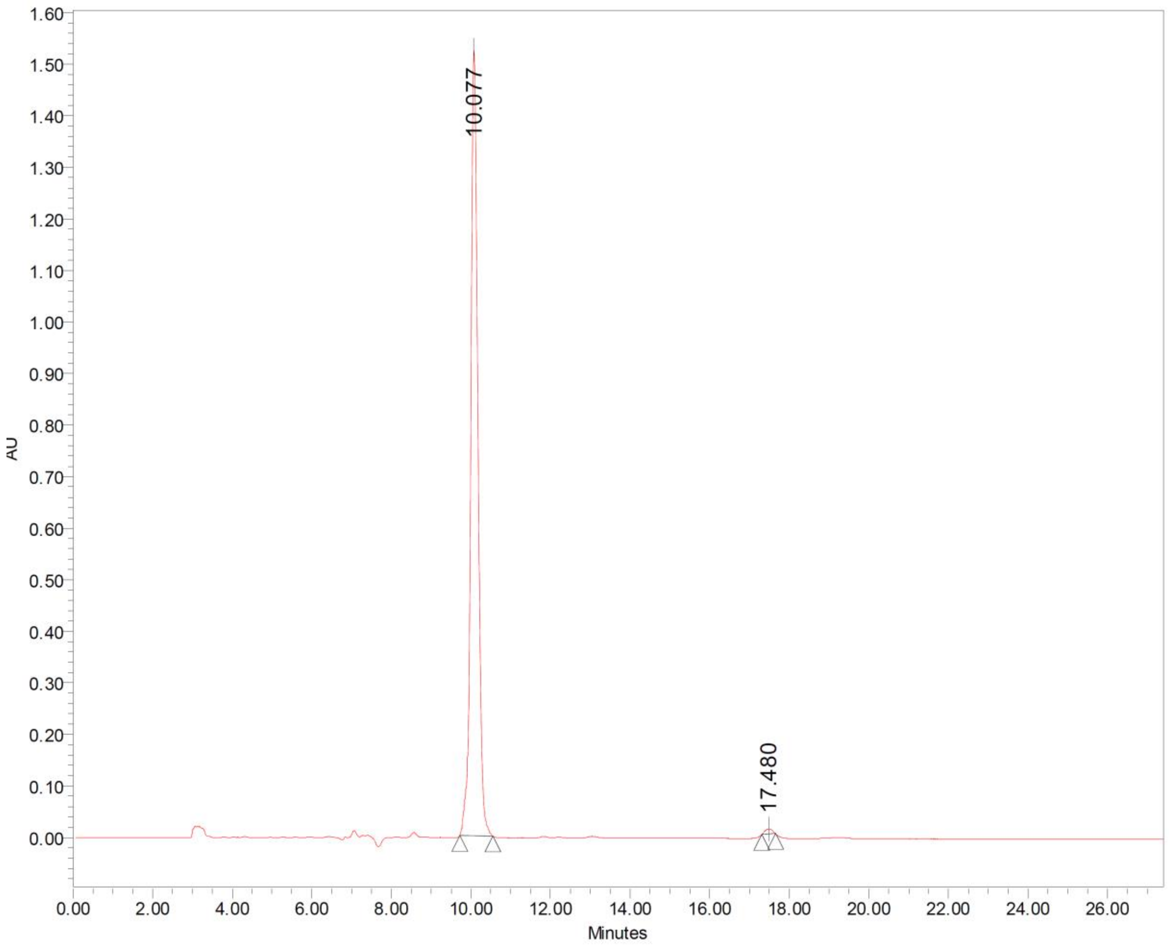

Sample Name: AB_AYA_02022_CHI_IC-3_15_1; Date Acquired: 09-06-2022 15:15:35 IST; Vial: 1 Injection: 2

Peak Summary with Statistics
Name:

	Sample Name	Vial	Inj	Retention Time (min)	Area	% Area	Height
1	AB_AYA_02022_CHI_IC-3_15_1	1	2	17.480	120583	0.62	9545
2	AB_AYA_02022_CHI_IC-3_15_1	1	2	10.077	19300274	99.38	1524075
Mean				13.779			
Std. Dev.				5.235			

Reported by User: System
Report Method: Peak Summary Report
Report Method ID 1009
Page: 1 of 2

Project Name: AB Research Group
Date Printed:
12-06-2022
13:36:54 Asia/Calcutta

HPLC data of (−)-**8a**

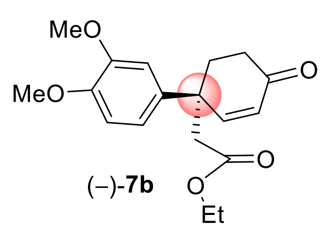

(–)-**7b**

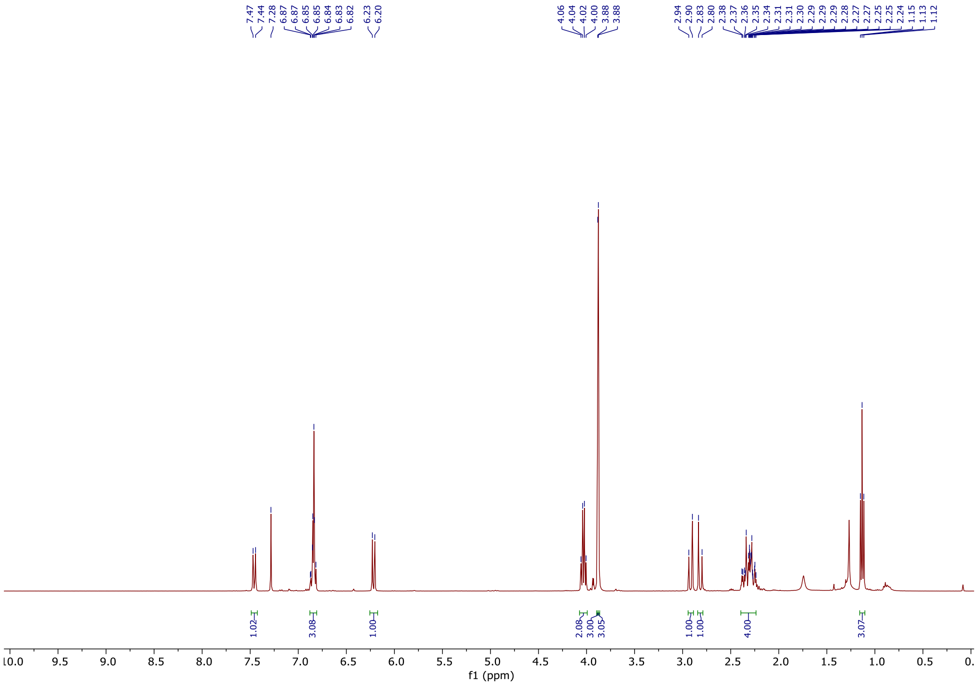

^{1}H NMR (400 MHz, $CDCl_3$) of compound (–)-**7b**

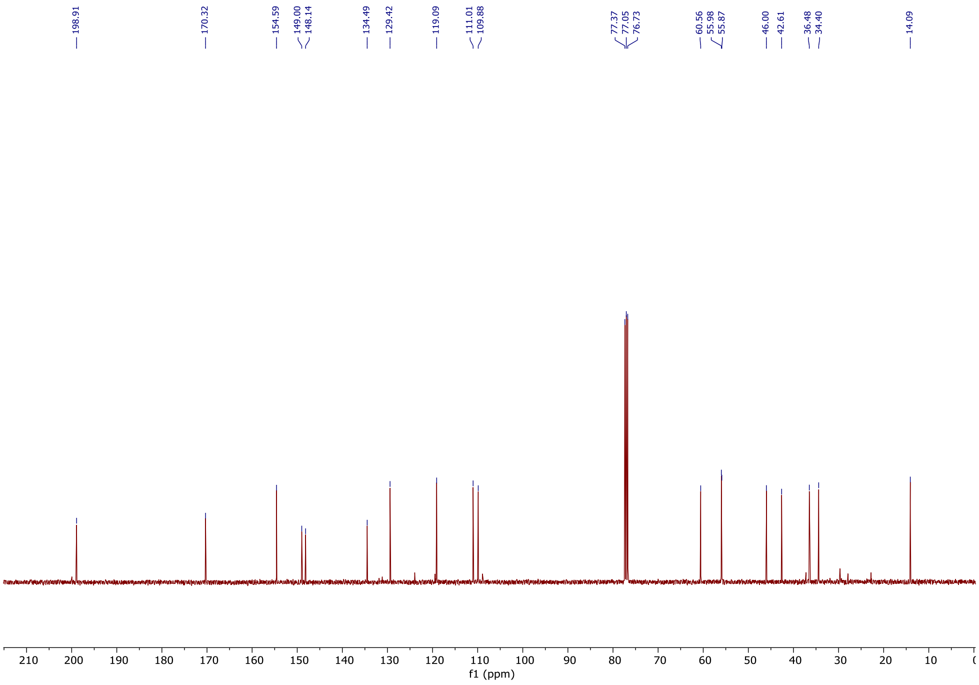

^{13}C{^{1}H} NMR (100 MHz, $CDCl_3$) of compound (–)-**7b**

Display Report

Analysis Info

		Acquisition Date	9/1/2021 3:02:13 PM
Analysis Name	D:\Data\new user data 2021\Sept-2021\01Sept\Dr.A.Bisai-AB-SM-02117_1-B,3_01_8787.d		
Method	hrlcms-20 sept--union-small molecules 01-sept-2021.m	Operator	RUCHI
Sample Name	Dr.A.Bisai-AB-SM-02117	Instrument	micrOTOF-Q II 10330
Comment			

Acquisition Parameter

Source Type	ESI	Ion Polarity	Positive	Set Nebulizer	0.4 Bar
Focus	Not active	Set Capillary	4600 V	Set Dry Heater	180 °C
Scan Begin	50 m/z	Set End Plate Offset	-500 V	Set Dry Gas	4.0 l/min
Scan End	3000 m/z	Set Collision Cell RF	100.0 Vpp	Set Divert Valve	Waste

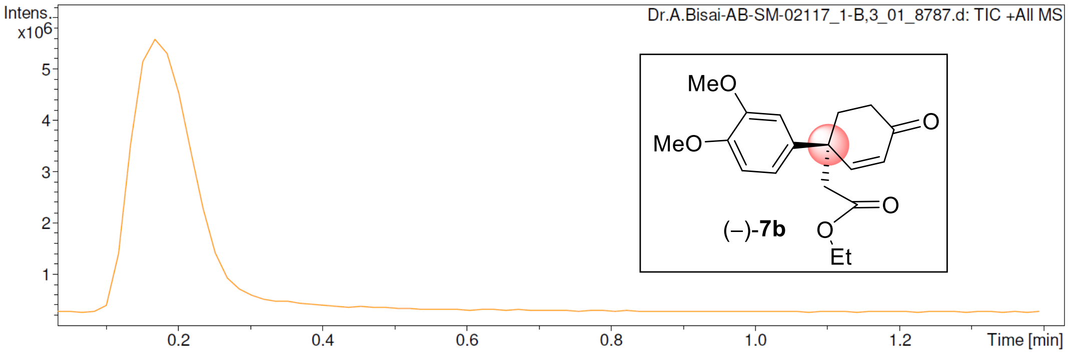

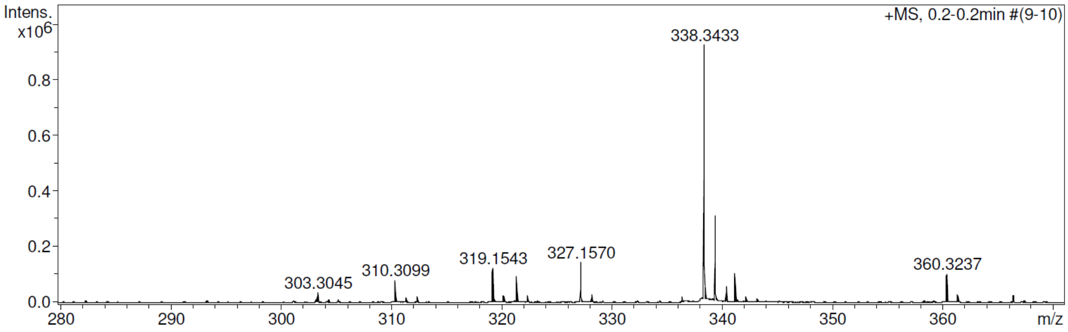

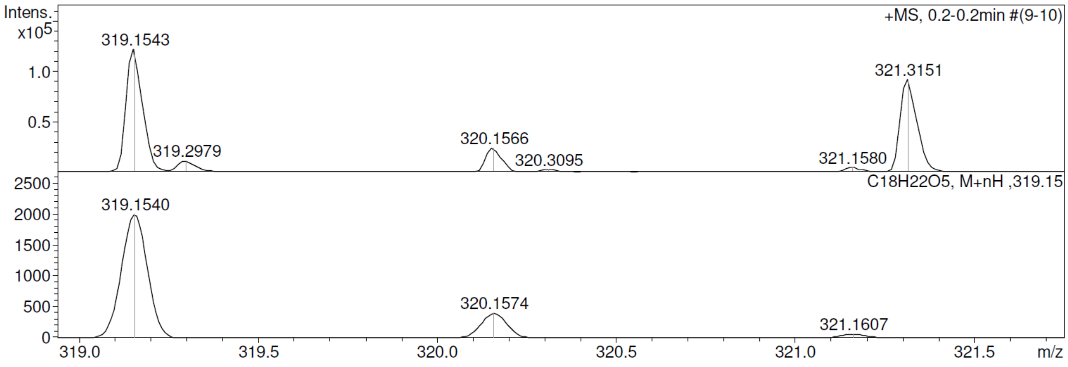

Bruker Compass DataAnalysis 4.0	printed: 9/1/2021 3:04:26 PM	Page 1 of 1

HRMS (ESI, m/z): of compound **7b**

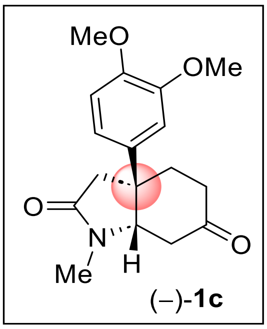

(–)-**1c**

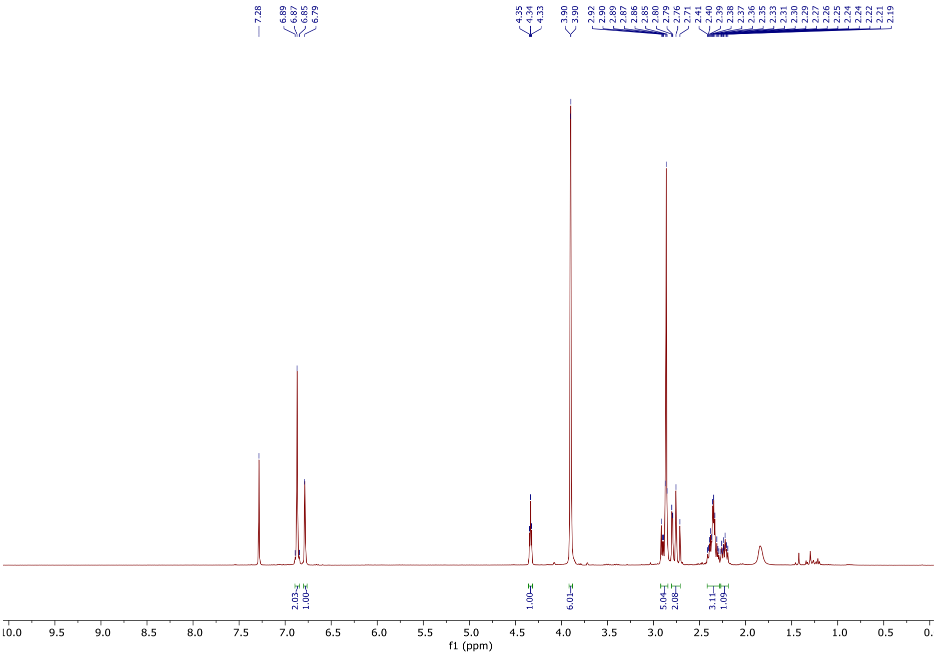

^{1}H NMR (400 MHz, CDCl$_3$) of compound (–)-**1c**

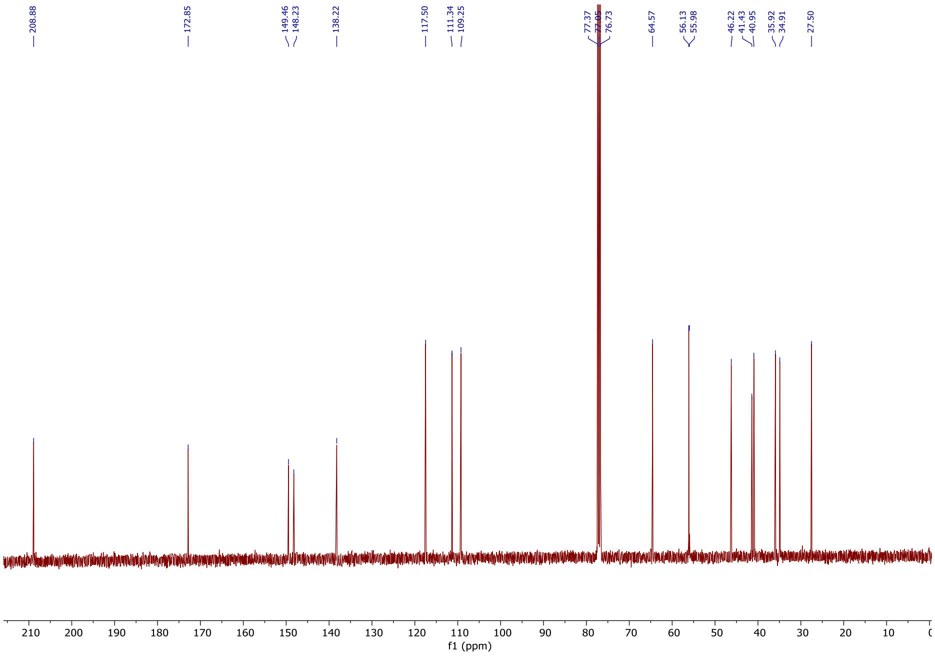

^{13}C{^{1}H} NMR (125 MHz, CDCl$_3$) of compound (–)-**1c**

Display Report

Analysis Info

Analysis Name	D:\Data\new user data 2021\Sept-2021\01Sept\Dr.A.Bisai-AB-SM-02118_1-B,2_01_8786.d	Acquisition Date	9/1/2021 2:59:01 PM
Method	hrlcms-20 sept--union-small molecules 01-sept-2021.m	Operator	RUCHI
Sample Name	Dr.A.Bisai-AB-SM-02118	Instrument	micrOTOF-Q II 10330
Comment			

Acquisition Parameter

Source Type	ESI	Ion Polarity	Positive	Set Nebulizer	0.4 Bar
Focus	Not active	Set Capillary	4600 V	Set Dry Heater	180 °C
Scan Begin	50 m/z	Set End Plate Offset	-500 V	Set Dry Gas	4.0 l/min
Scan End	3000 m/z	Set Collision Cell RF	100.0 Vpp	Set Divert Valve	Waste

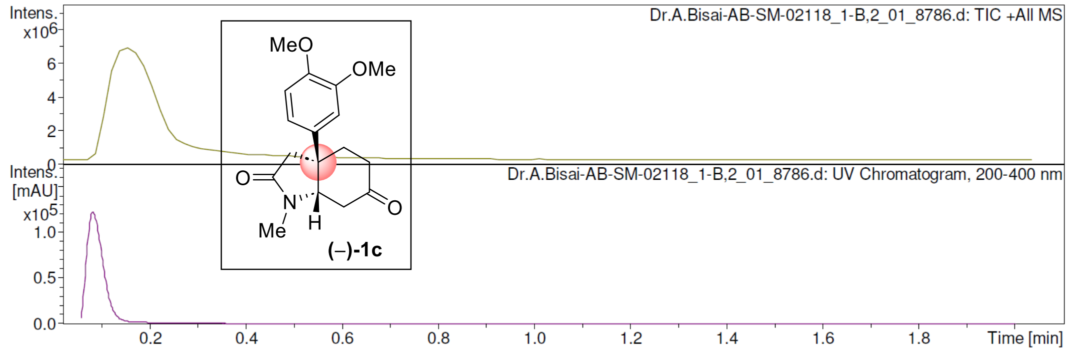

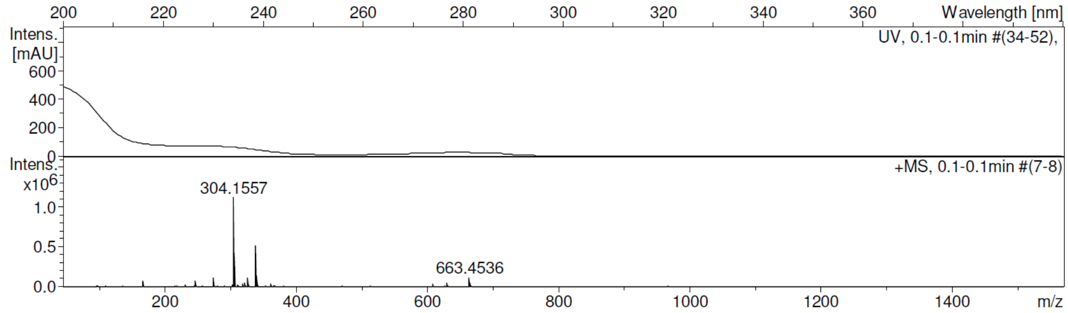

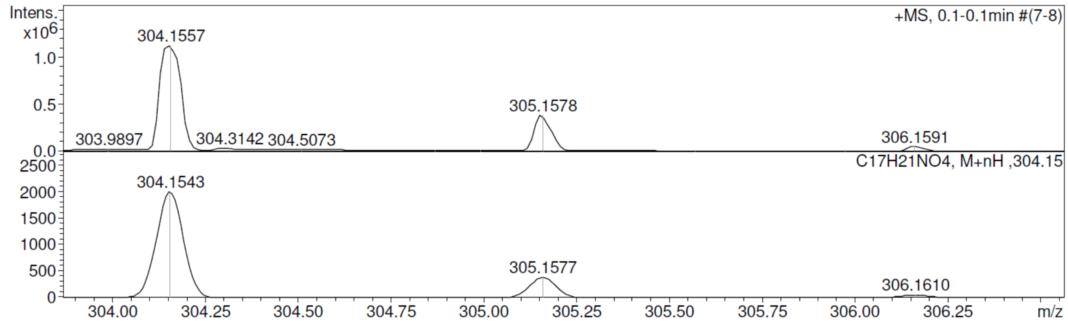

Bruker Compass DataAnalysis 4.0 printed: 9/1/2021 3:03:11 PM Page 1 of 1

HRMS (ESI, m/z): of compound **1c**

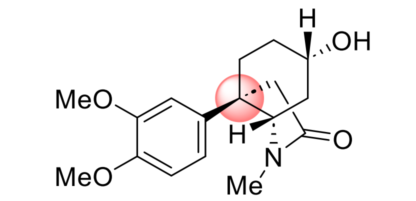

(–)-2-oxo-epimesembranol (**1d**)

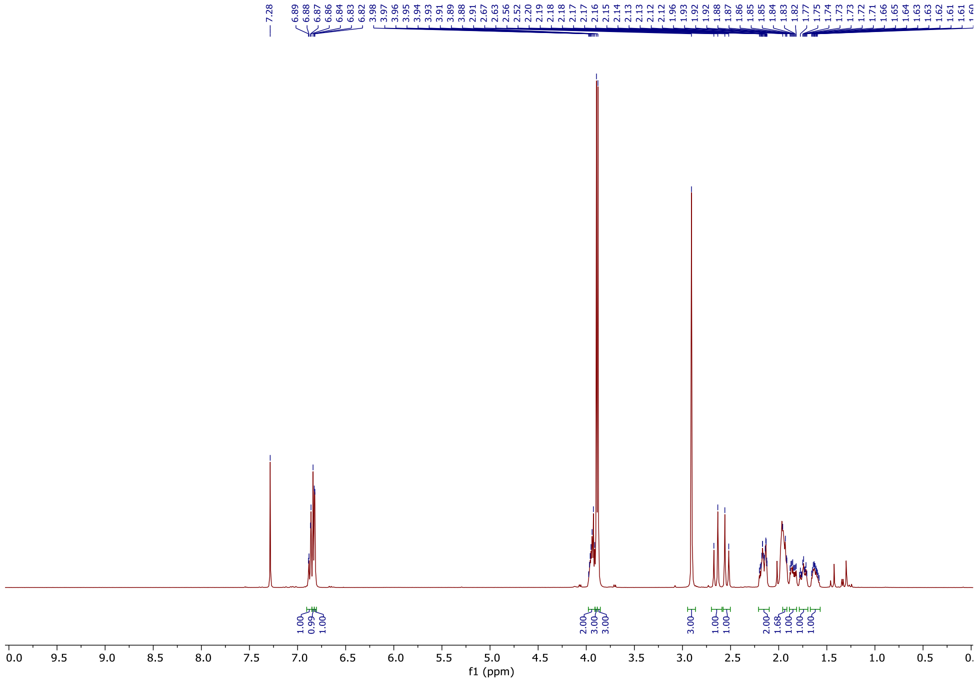

^{1}H NMR (400 MHz, $CDCl_3$) of compound (–)-**1d**

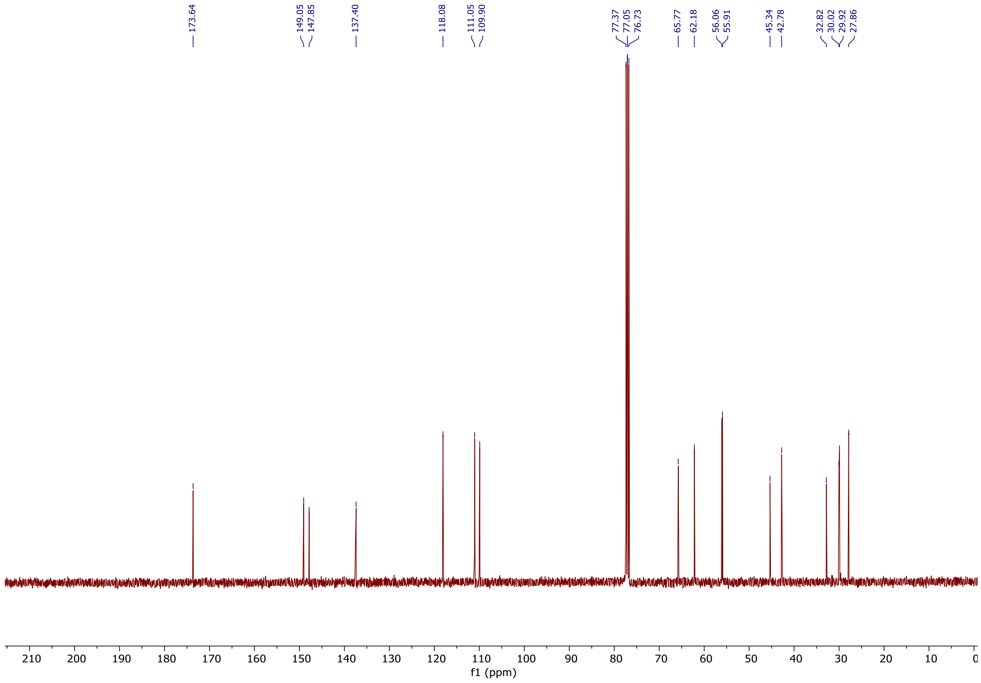

^{13}C{^{1}H} NMR (100 MHz, $CDCl_3$) of compound (–)-**1d**

Display Report

Analysis Info

		Acquisition Date	9/1/2021 3:05:25 PM
Analysis Name	D:\Data\new user data 2021\Sept-2021\01Sept\Dr.A.Bisai-AB-SM-02142_1-B,4_01_8788.d		
Method	hrlcms-20 sept--union-small molecules 01-sept-2021.m	Operator	RUCHI
Sample Name	Dr.A.Bisai-AB-SM-02142	Instrument	micrOTOF-Q II 10330
Comment			

Acquisition Parameter

Source Type	ESI	Ion Polarity	Positive	Set Nebulizer	0.4 Bar
Focus	Not active	Set Capillary	4600 V	Set Dry Heater	180 °C
Scan Begin	50 m/z	Set End Plate Offset	-500 V	Set Dry Gas	4.0 l/min
Scan End	3000 m/z	Set Collision Cell RF	100.0 Vpp	Set Divert Valve	Waste

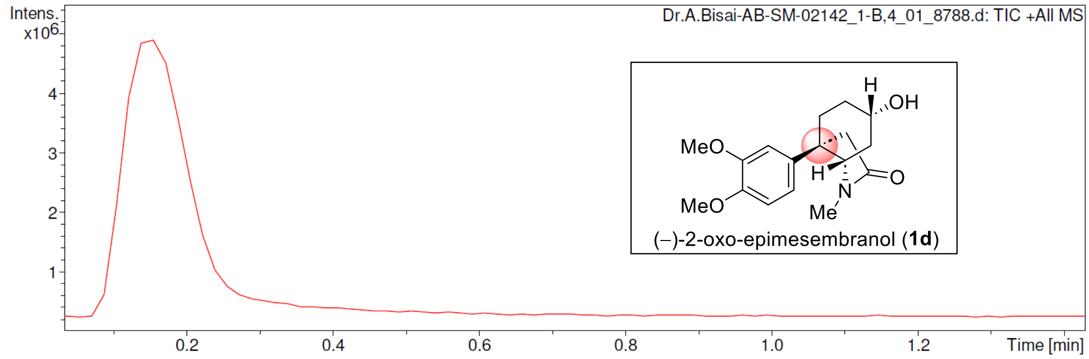

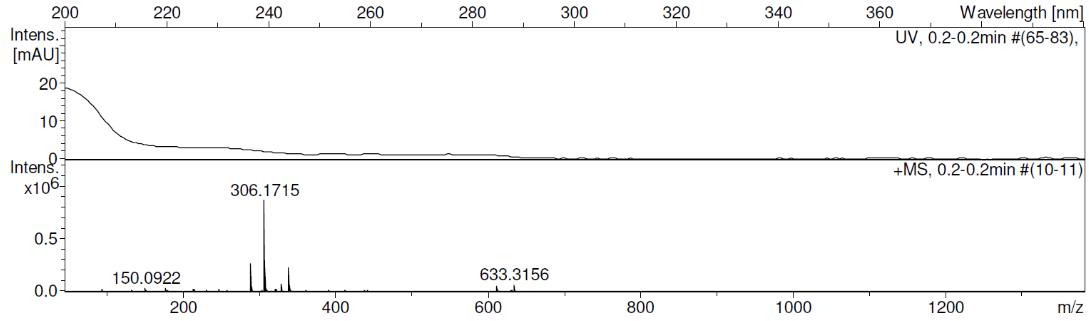

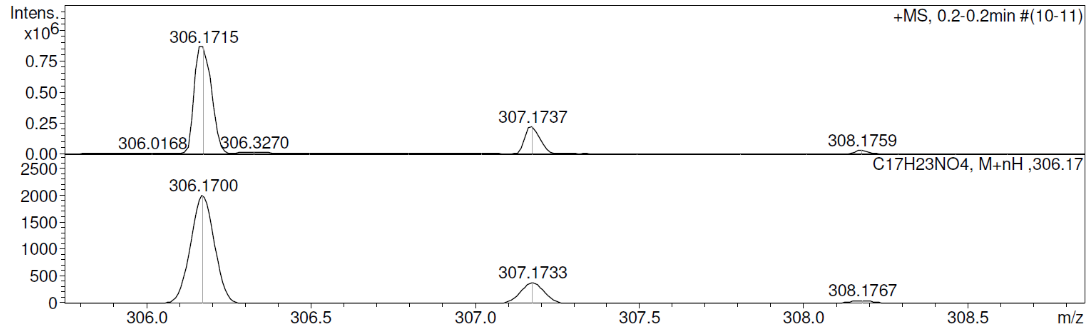

Bruker Compass DataAnalysis 4.0	printed: 9/1/2021 3:30:20 PM	Page 1 of 1

HRMS (ESI, m/z): of compound **1d**

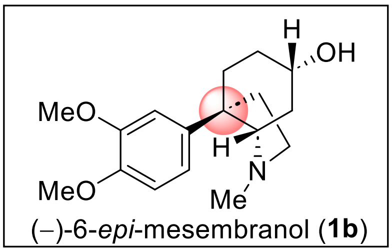

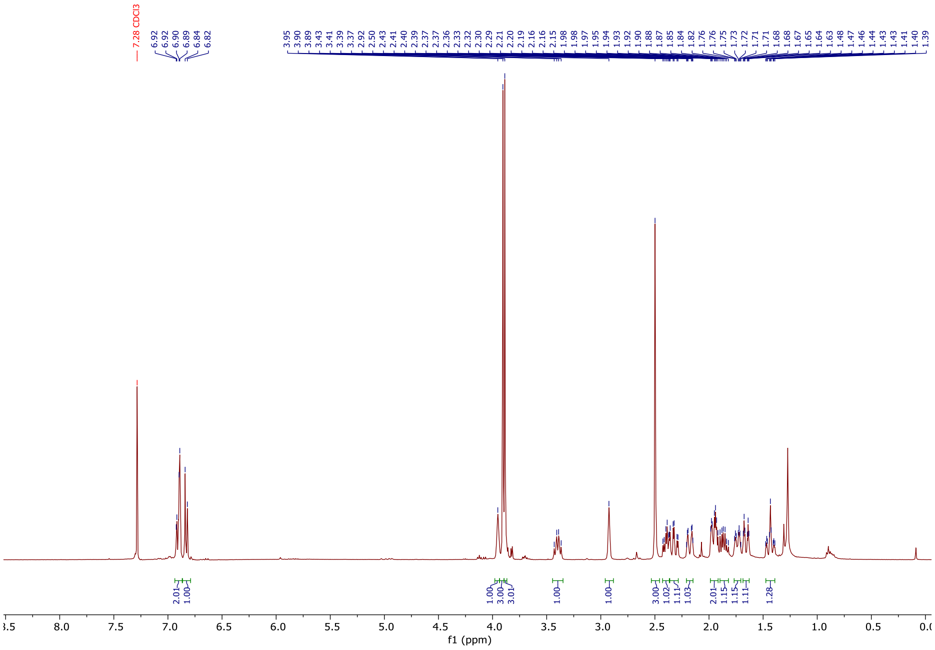

^{1}H NMR (400 MHz, $CDCl_3$) of compound (–)-**1b**

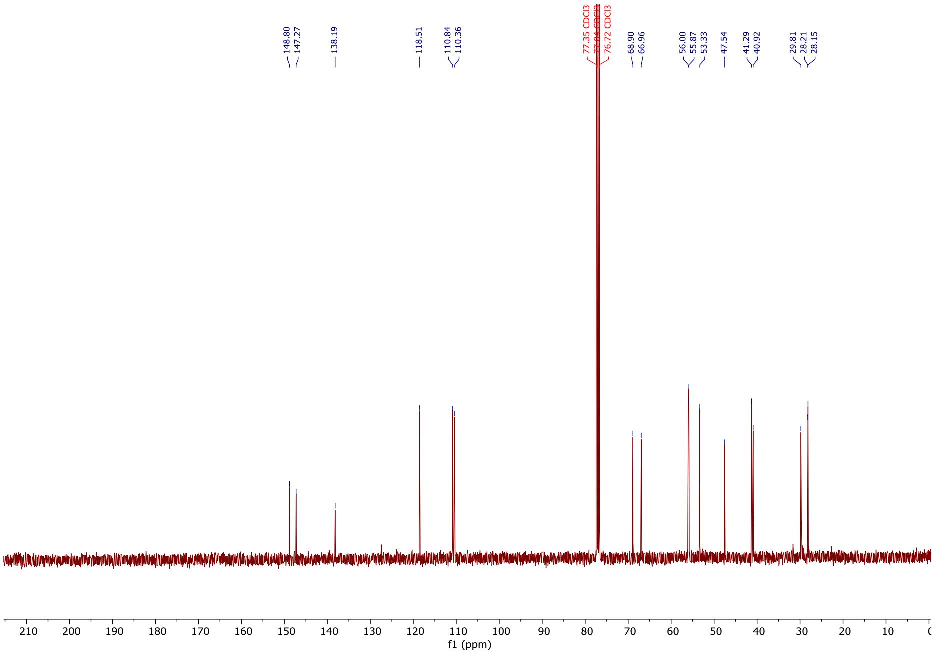

^{13}C{^{1}H} NMR (100 MHz, $CDCl_3$) of compound (–)-**1b**

Display Report

Analysis Info

		Acquisition Date	9/13/2021 11:58:21 AM
Analysis Name	D:\Data\new user data 2021\Sept-2021\13-Sept\Dr.A.Bisai-AB-SM-02144_1-A,4_01_9170.d		
Method	hrlcms-20 sept--union-esi-tune low 01-sept-2021.m	Operator	RUCHI
Sample Name	Dr.A.Bisai-AB-SM-02144	Instrument	micrOTOF-Q II 10330
Comment			

Acquisition Parameter

Source Type	ESI	Ion Polarity	Positive	Set Nebulizer	0.4 Bar
Focus	Not active	Set Capillary	4600 V	Set Dry Heater	180 °C
Scan Begin	50 m/z	Set End Plate Offset	-500 V	Set Dry Gas	4.0 l/min
Scan End	3000 m/z	Set Collision Cell RF	100.0 Vpp	Set Divert Valve	Waste

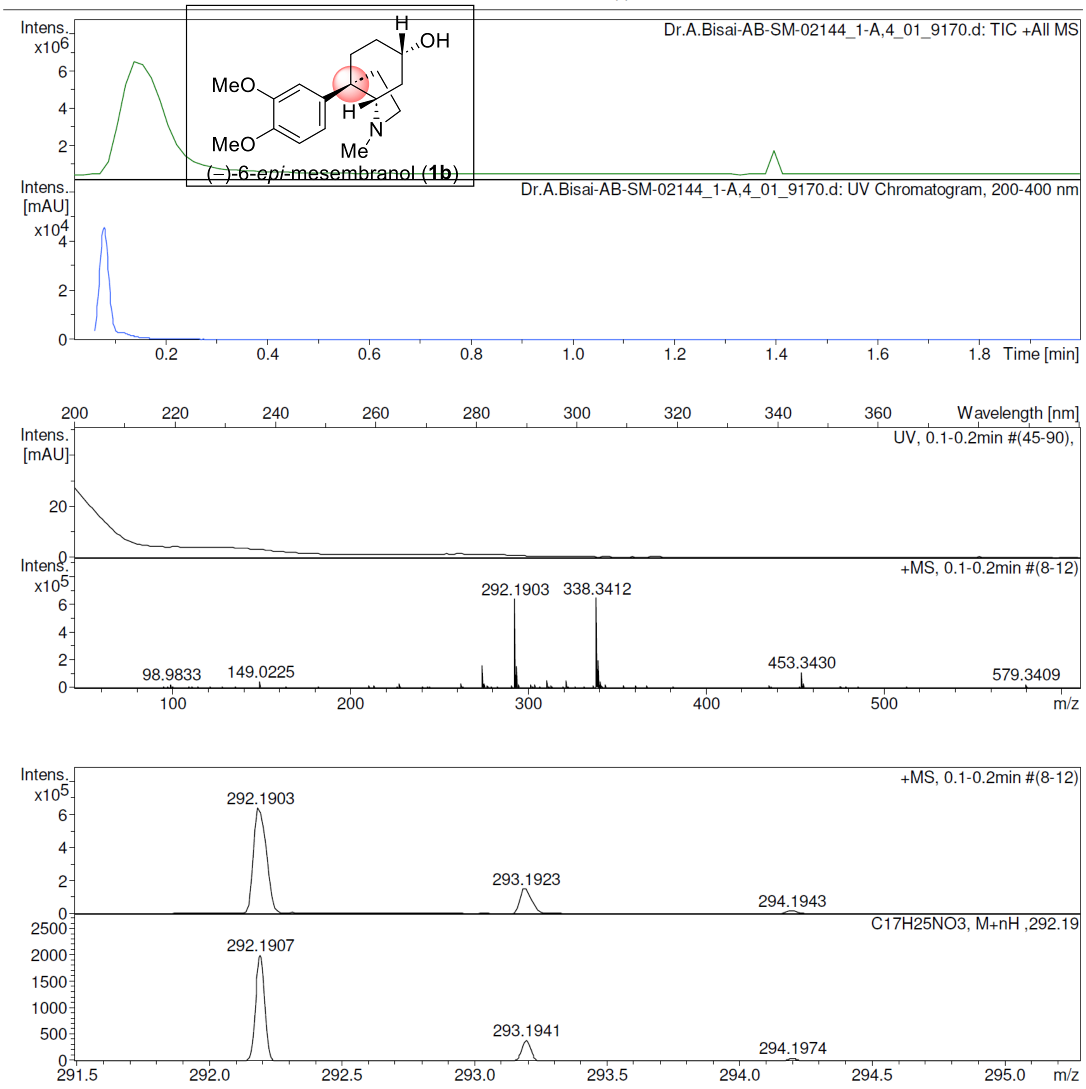

Bruker Compass DataAnalysis 4.0 printed: 9/13/2021 2:10:59 PM Page 1 of 1

HRMS (ESI, m/z): of compound **1b**

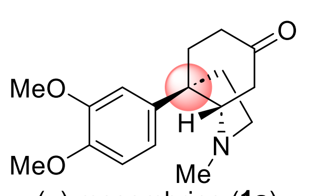

(–)-mesembrine (**1a**)

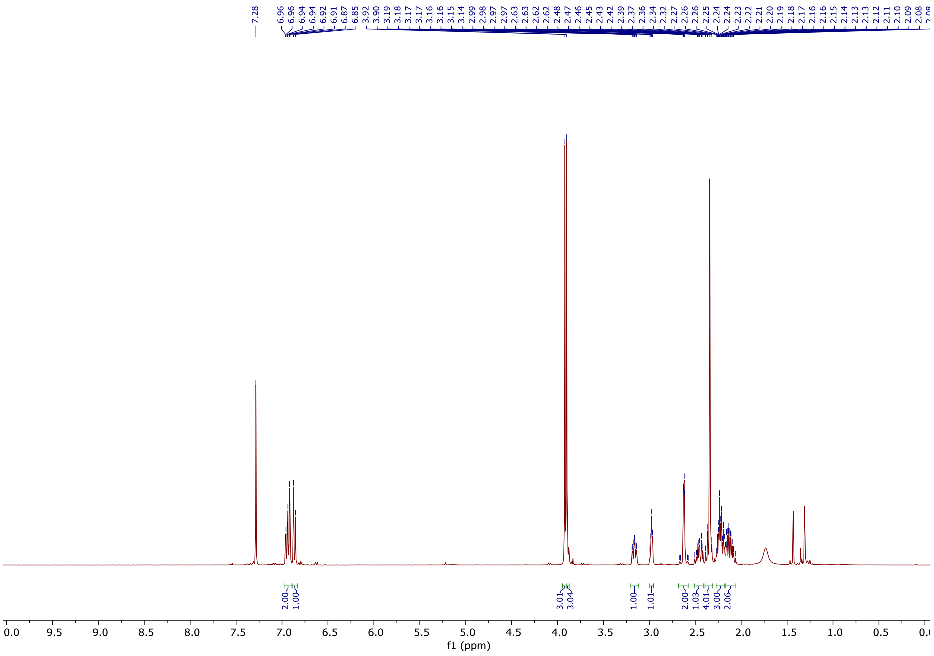

^{1}H NMR (400 MHz, $CDCl_3$) of compound (–)-**1a**

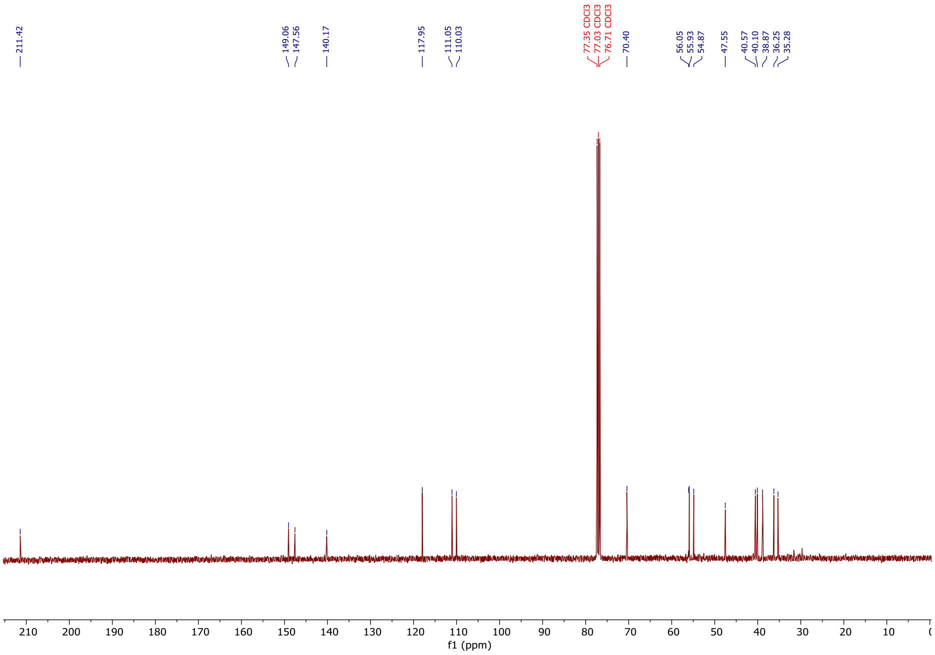

^{13}C{^{1}H} NMR (100 MHz, $CDCl_3$) of compound (–)-**1a**

Display Report

Analysis Info

Analysis Name	D:\Data\new user data 2021\Sept-2021\13-Sept\Dr.A.Bisai-AB-SM-02155_1-A,5_01_9171.d	Acquisition Date	9/13/2021 12:01:34 PM
Method	hrlcms-20 sept--union-esi-tune low 01-sept-2021.m	Operator	RUCHI
Sample Name	Dr.A.Bisai-AB-SM-02155	Instrument	micrOTOF-Q II 10330
Comment			

Acquisition Parameter

Source Type	ESI	Ion Polarity	Positive	Set Nebulizer	0.4 Bar
Focus	Not active	Set Capillary	4600 V	Set Dry Heater	180 °C
Scan Begin	50 m/z	Set End Plate Offset	-500 V	Set Dry Gas	4.0 l/min
Scan End	3000 m/z	Set Collision Cell RF	100.0 Vpp	Set Divert Valve	Waste

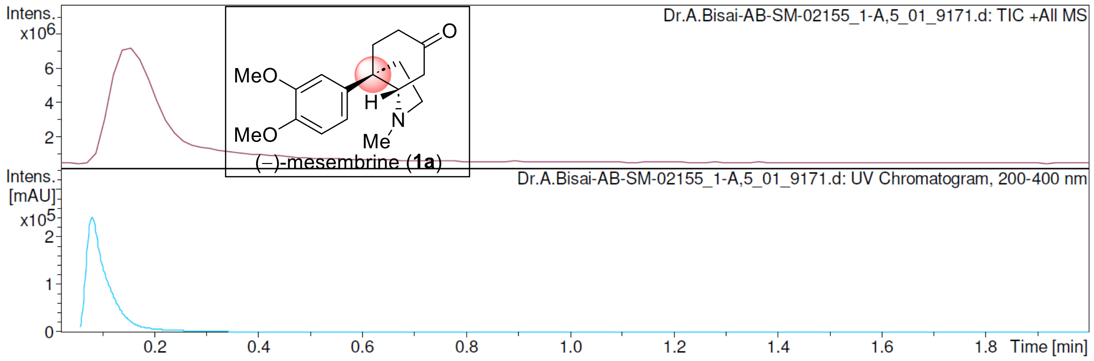

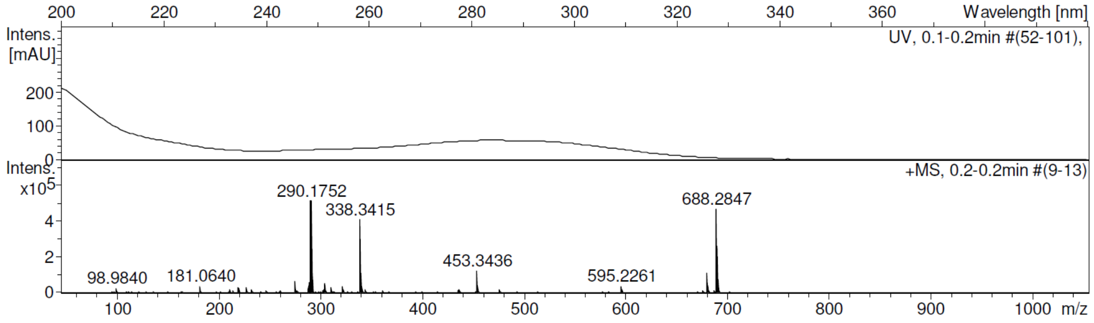

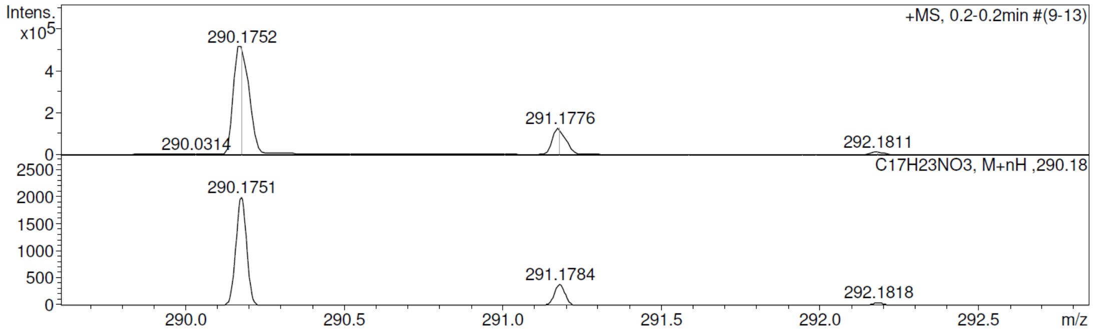

Bruker Compass DataAnalysis 4.0 printed: 9/13/2021 2:08:49 PM Page 1 of 1

HRMS (ESI, m/z): of compound **1a**

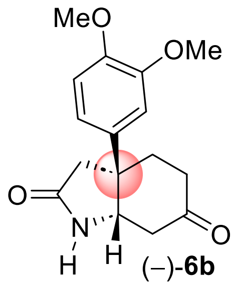

(–)-**6b**

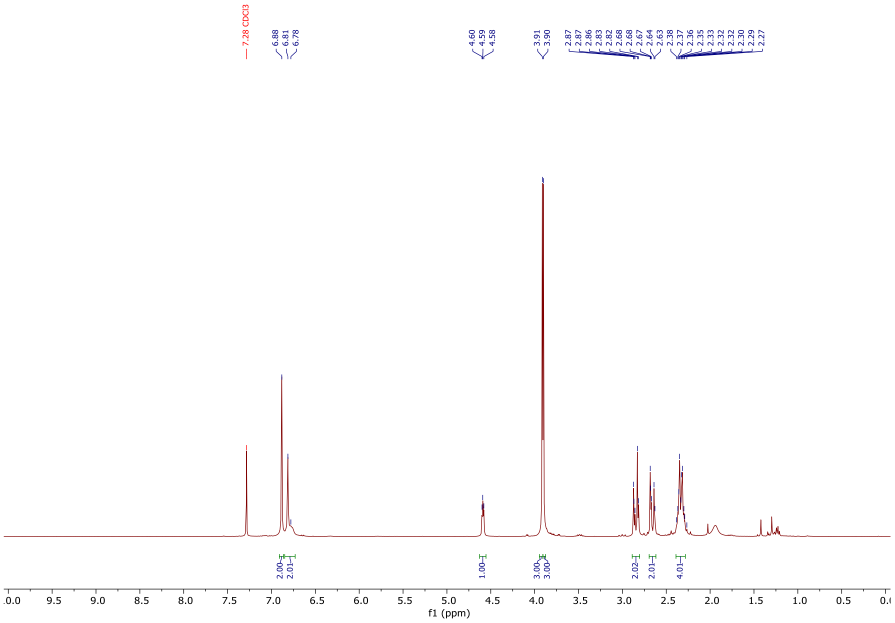

^{1}H NMR (400 MHz, $CDCl_3$) of compound (–)-**6b**

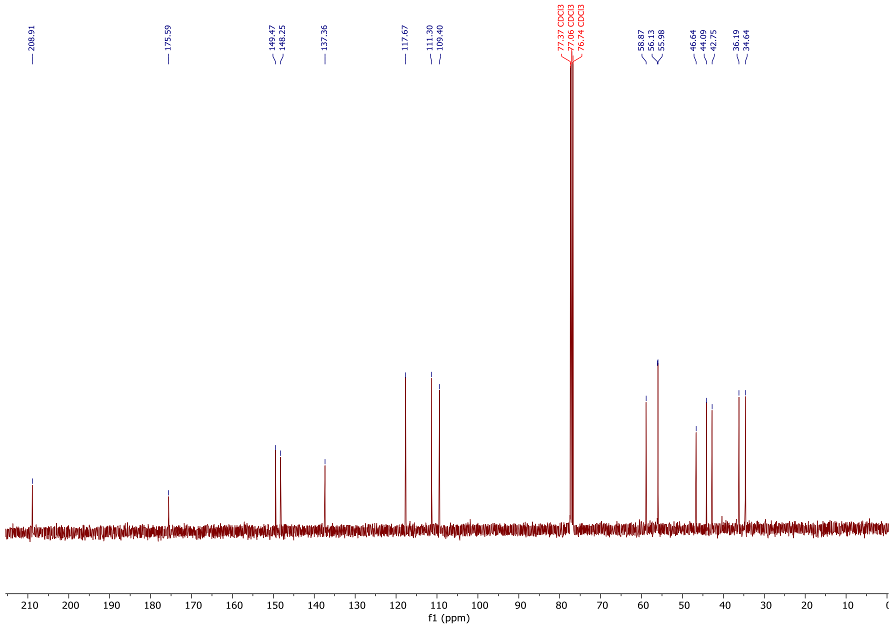

^{13}C{^{1}H} NMR (100 MHz, $CDCl_3$) of compound (–)-**6b**

Display Report

Analysis Info

Analysis Name	D:\Data\new user data 2021\Sept-2021\13-Sept\Dr.A.Bisai-AB-SM-02119_1-A,2_01_9168.d	Acquisition Date	9/13/2021 11:51:53 AM
Method	hrlcms-20 sept--union-esi-tune low 01-sept-2021.m	Operator	RUCHI
Sample Name	Dr.A.Bisai-AB-SM-02119	Instrument	micrOTOF-Q II 10330
Comment			

Acquisition Parameter

Source Type	ESI	Ion Polarity	Positive	Set Nebulizer	0.4 Bar
Focus	Not active	Set Capillary	4600 V	Set Dry Heater	180 °C
Scan Begin	50 m/z	Set End Plate Offset	-500 V	Set Dry Gas	4.0 l/min
Scan End	3000 m/z	Set Collision Cell RF	100.0 Vpp	Set Divert Valve	Waste

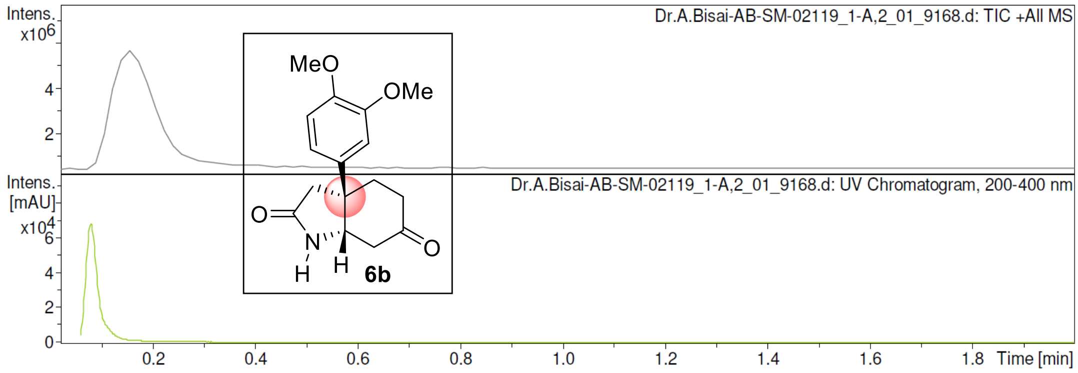

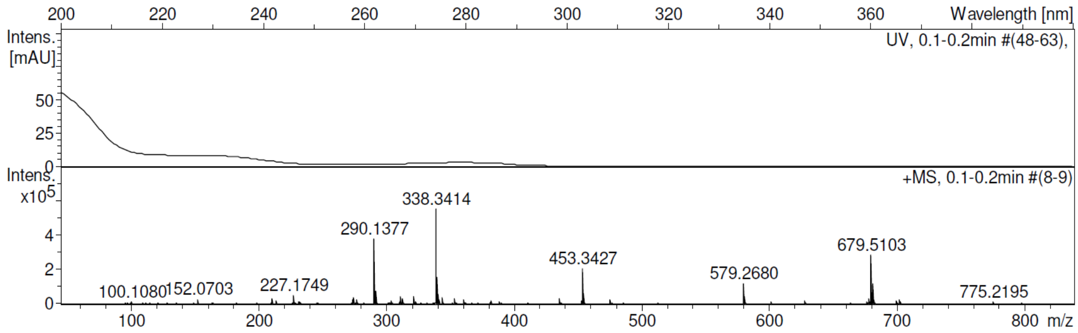

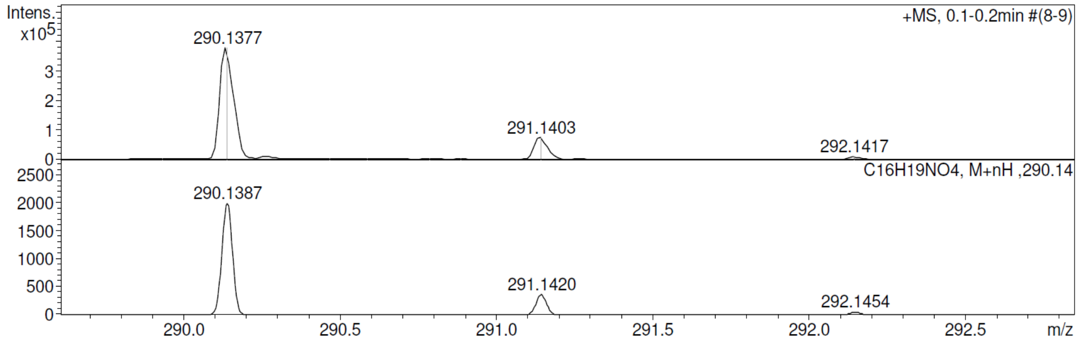

Bruker Compass DataAnalysis 4.0 printed: 9/13/2021 2:09:24 PM Page 1 of 1

HRMS (ESI, m/z): of compound **6b**

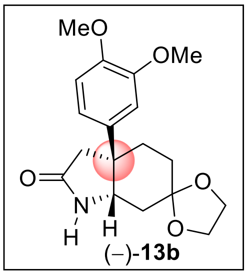

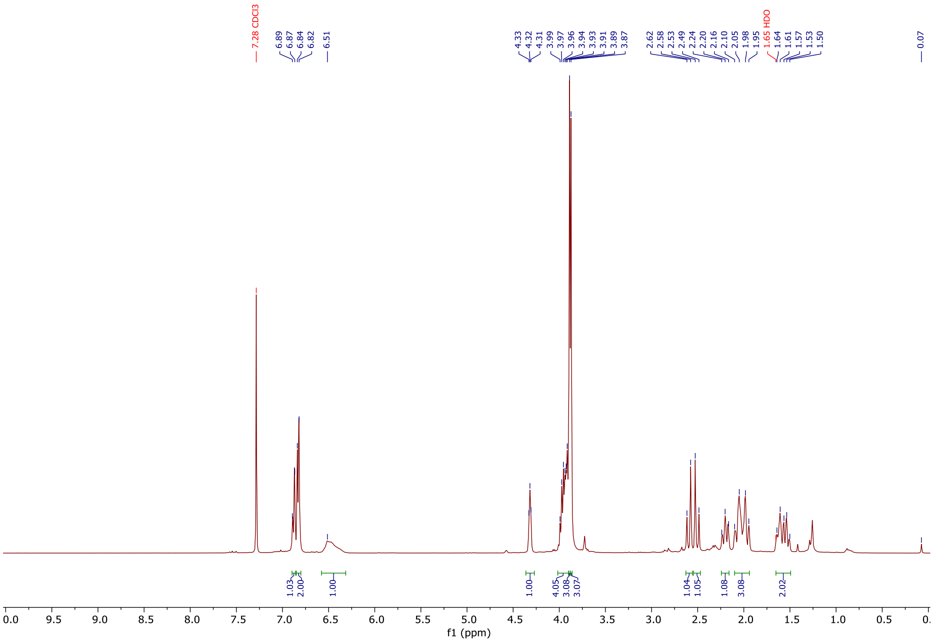

^{1}H NMR (400 MHz, CDCl$_3$) of compound (–)-**13b**

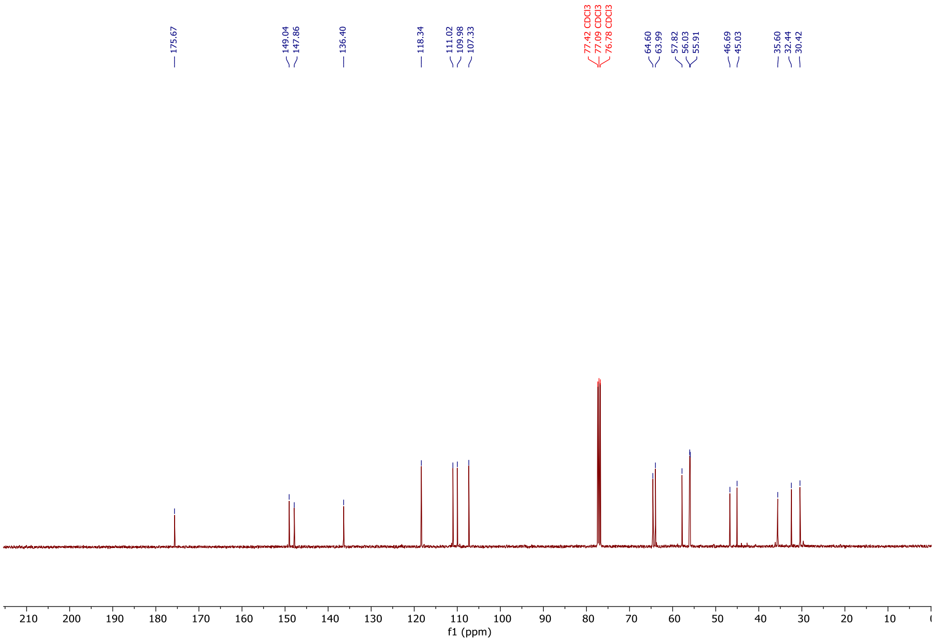

^{13}C{^{1}H} NMR (100 MHz, CDCl$_3$) of compound (–)-**13b**

Display Report

Analysis Info

Analysis Name	D:\Data\new user data 2021\Nov-2021\23-nov\Prof.A.Bisai-AB-SM02124_1-A,7_01_10245.d	Acquisition Date	11/23/2021 2:53:37 PM
Method	hrlcms-20 sept.m	Operator	RUCHI
Sample Name	Prof.A.Bisai-AB-SM02124	Instrument	micrOTOF-Q II 10330
Comment			

Acquisition Parameter

Source Type	ESI	Ion Polarity	Positive	Set Nebulizer	1.2 Bar
Focus	Active	Set Capillary	4500 V	Set Dry Heater	200 °C
Scan Begin	50 m/z	Set End Plate Offset	-500 V	Set Dry Gas	7.0 l/min
Scan End	3000 m/z	Set Collision Cell RF	130.0 Vpp	Set Divert Valve	Waste

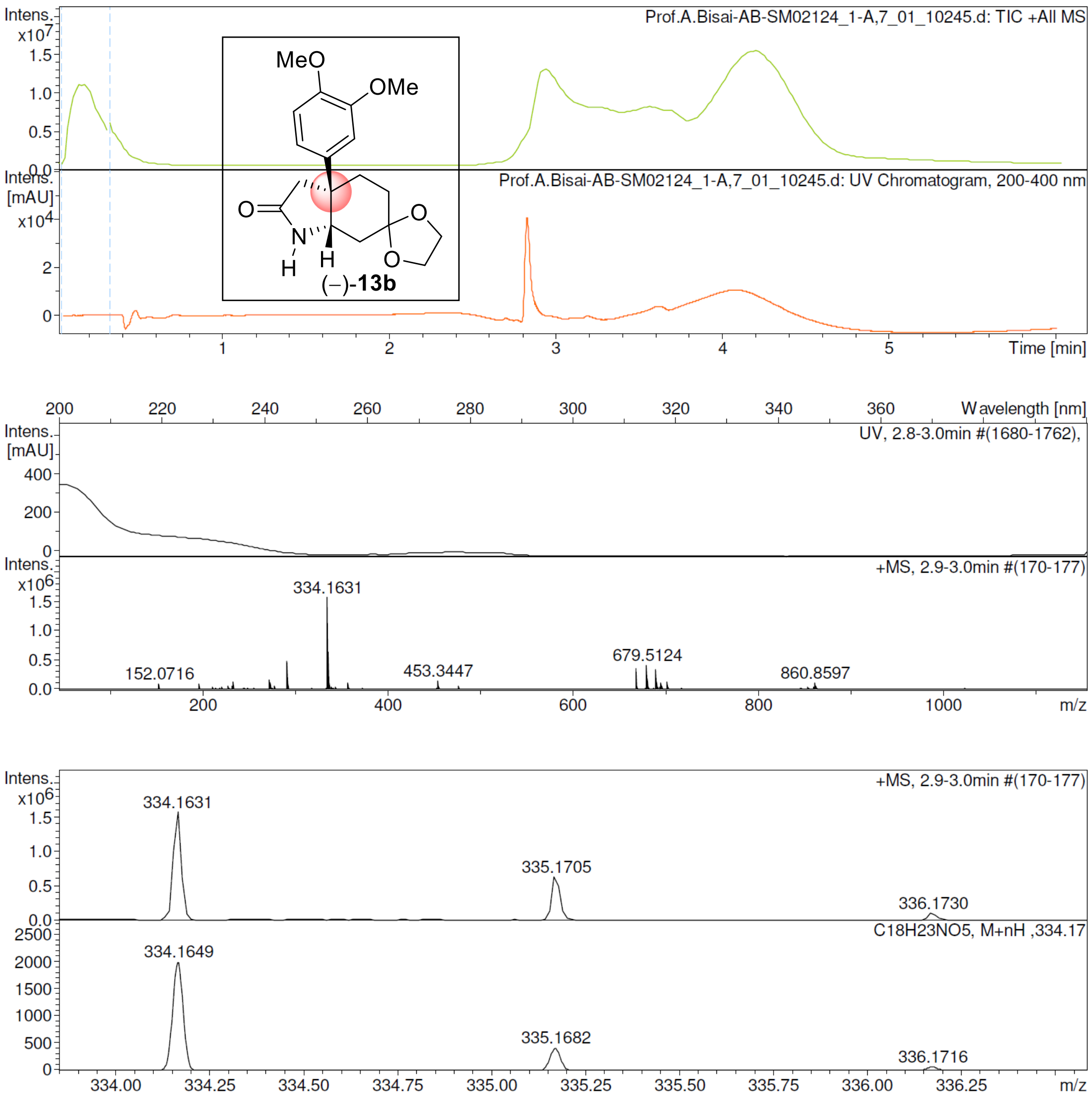

Bruker Compass DataAnalysis 4.0 printed: 11/23/2021 3:01:42 PM Page 1 of 1

HRMS (ESI, m/z): of compound **13b**

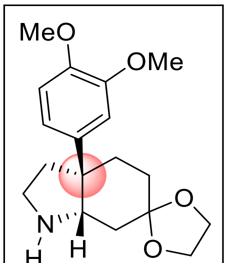

(−)-**14b**

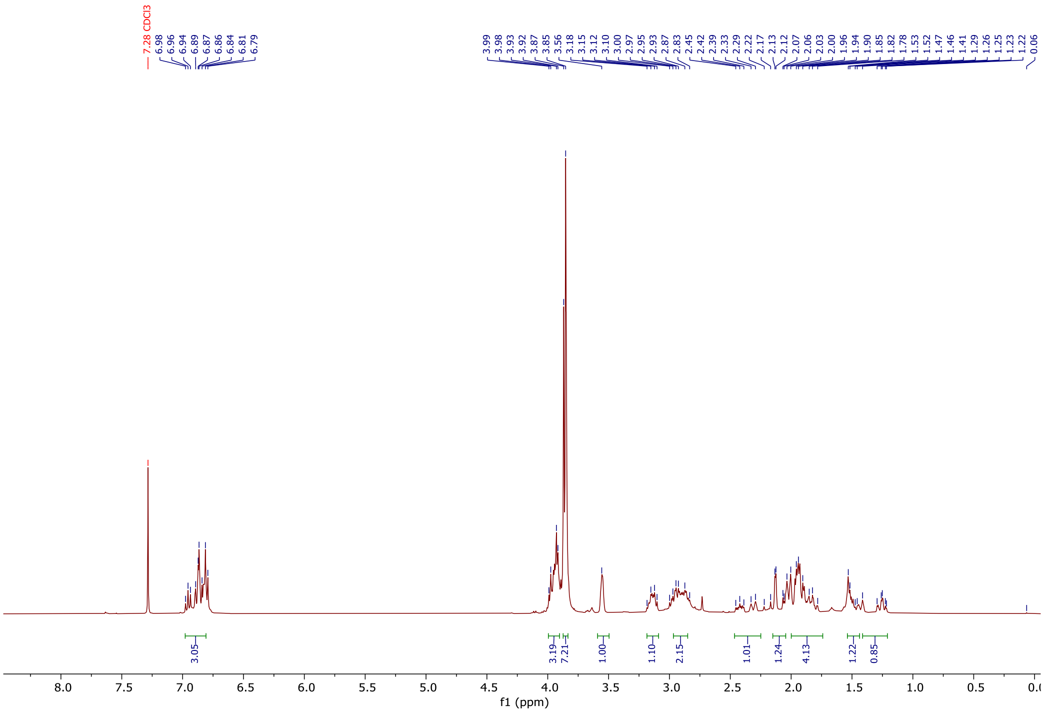

^{1}H NMR (400 MHz, $CDCl_3$) of compound (−)-**14b**

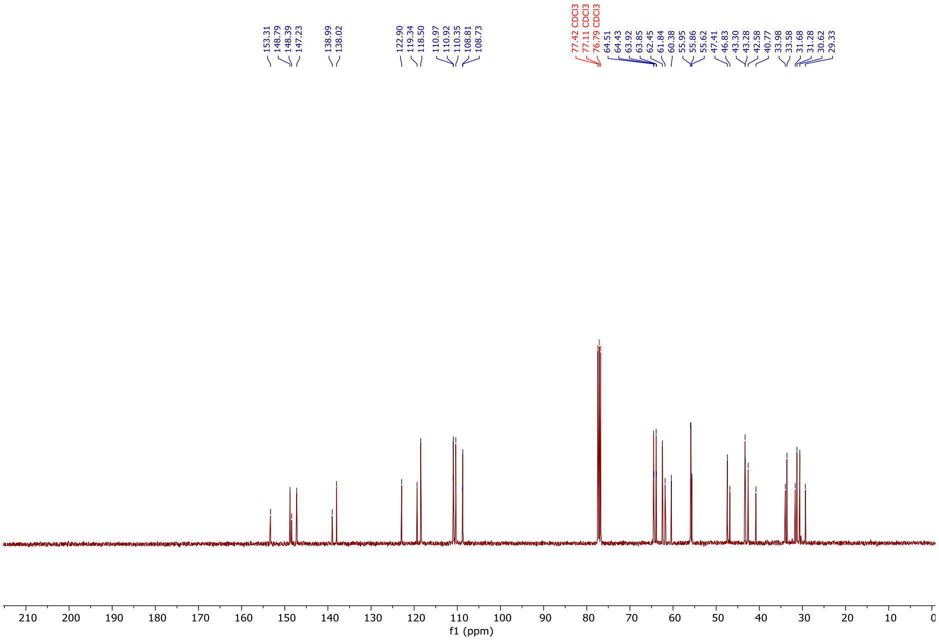

^{13}C{^{1}H} NMR (100 MHz, $CDCl_3$) of compound (−)-**14b**

Display Report

Analysis Info

Analysis Name	D:\Data\new user data 2021\Nov-2021\23-nov\Prof.A.Bisai-AB-SM02160_1-A,8_01_10246.d	Acquisition Date	11/23/2021 3:00:50 PM
Method	hrlcms-20 sept.m	Operator	RUCHI
Sample Name	Prof.A.Bisai-AB-SM02160	Instrument	micrOTOF-Q II 10330
Comment			

Acquisition Parameter

Source Type	ESI	Ion Polarity	Positive	Set Nebulizer	1.2 Bar
Focus	Active	Set Capillary	4500 V	Set Dry Heater	200 °C
Scan Begin	50 m/z	Set End Plate Offset	-500 V	Set Dry Gas	7.0 l/min
Scan End	3000 m/z	Set Collision Cell RF	130.0 Vpp	Set Divert Valve	Waste

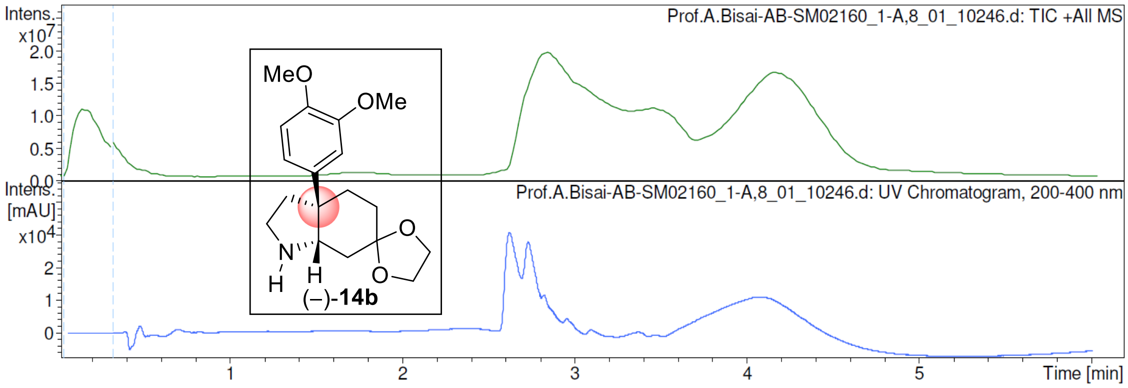

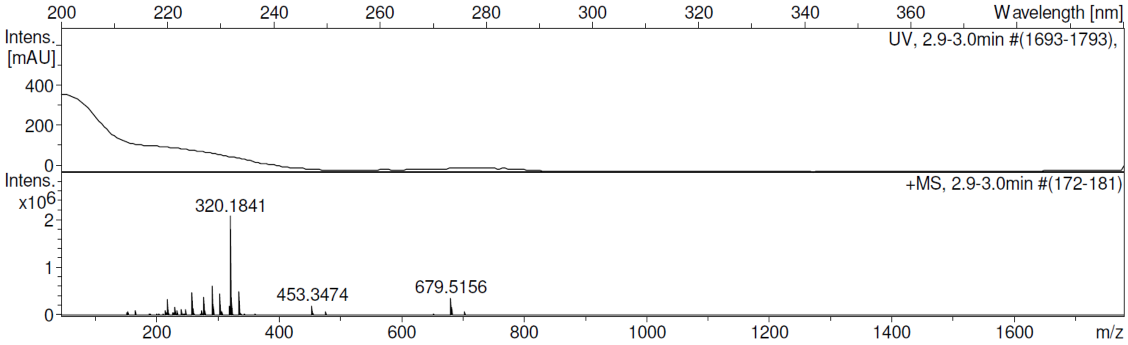

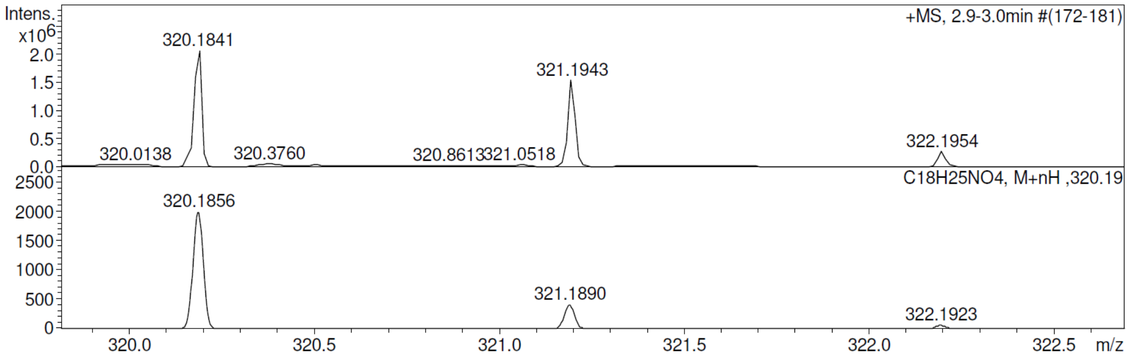

Bruker Compass DataAnalysis 4.0 printed: 11/23/2021 3:10:50 PM Page 1 of 1

HRMS (ESI, m/z): of compound **14b**

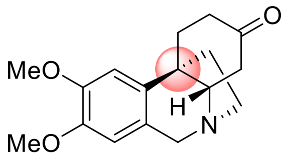

[(+)-**15b**]

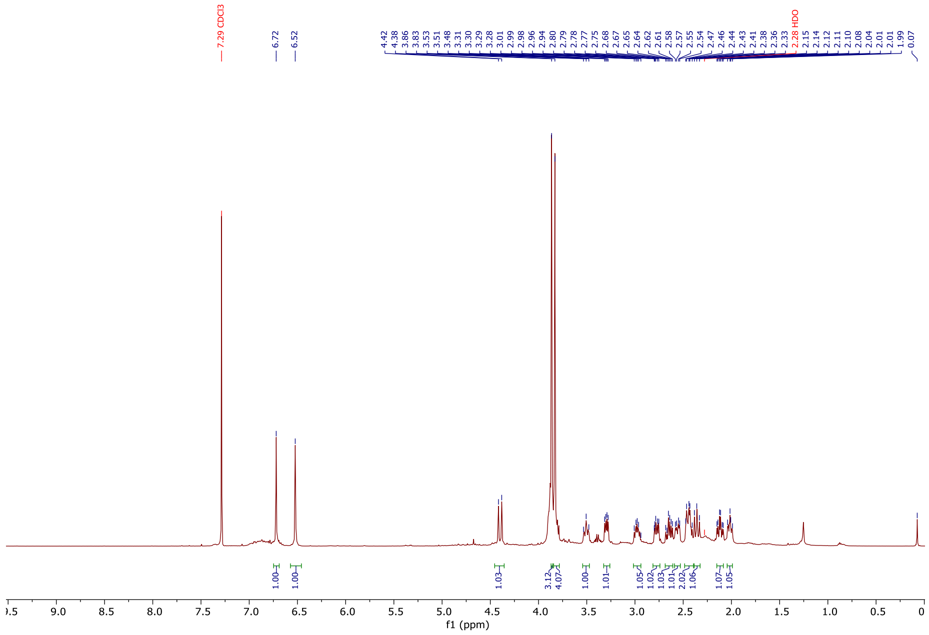

^{1}H NMR (500 MHz, $CDCl_3$) of compound (+)-**15b**

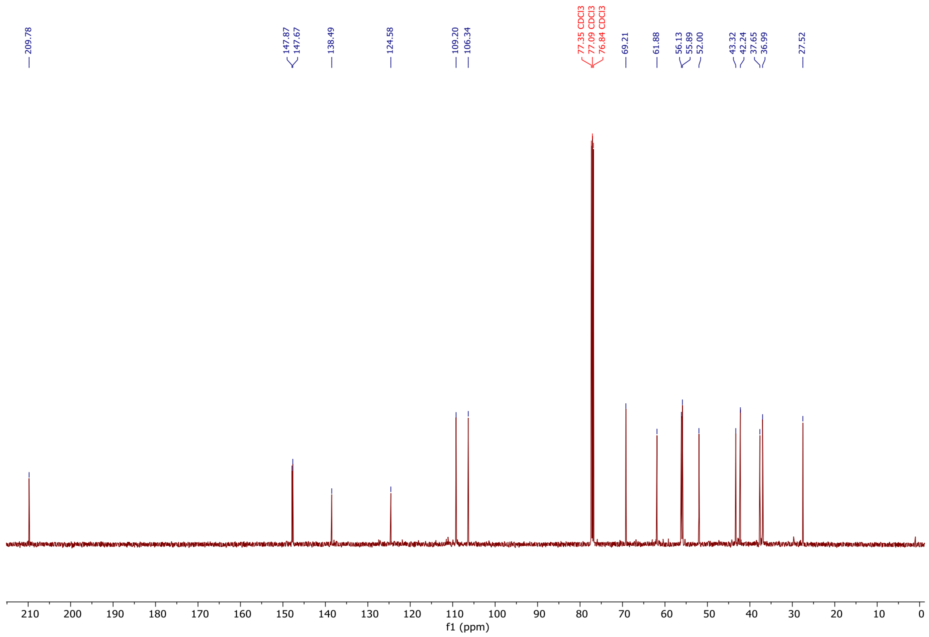

^{13}C{^{1}H} NMR (125 MHz, $CDCl_3$) of compound (+)-**15b**

Display Report

Analysis Info

		Acquisition Date	2/7/2022 1:21:52 PM
Analysis Name	D:\Data\NEW USER DATA 2022\fe-2022\07-feb-2022\Dr.A.Bisai-ABSM02190_1-A,7_01_11018.d		
Method	hrlcms-20 sept.m	Operator	RUCHI
Sample Name	Dr.A.Bisai-ABSM02190	Instrument	micrOTOF-Q II 10330
Comment			

Acquisition Parameter

Source Type	ESI	Ion Polarity	Positive	Set Nebulizer	1.2 Bar
Focus	Active	Set Capillary	4500 V	Set Dry Heater	200 °C
Scan Begin	50 m/z	Set End Plate Offset	-500 V	Set Dry Gas	6.0 l/min
Scan End	3000 m/z	Set Collision Cell RF	130.0 Vpp	Set Divert Valve	Waste

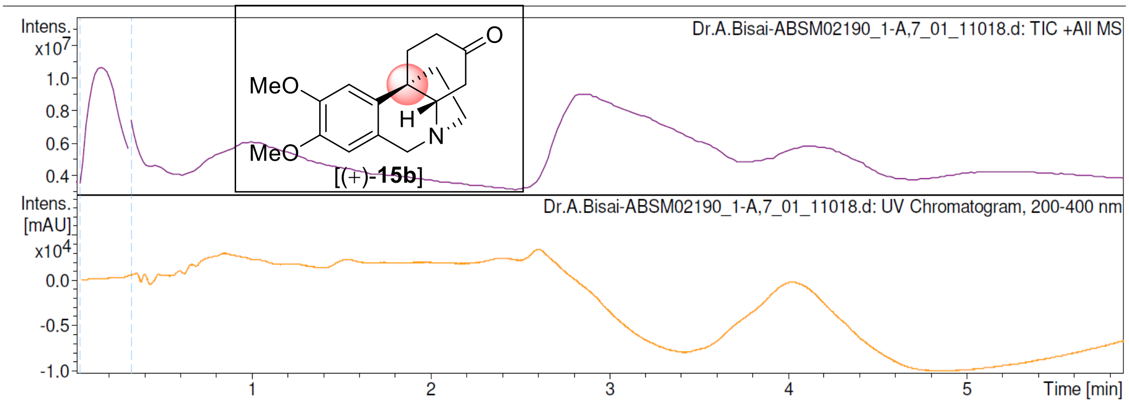

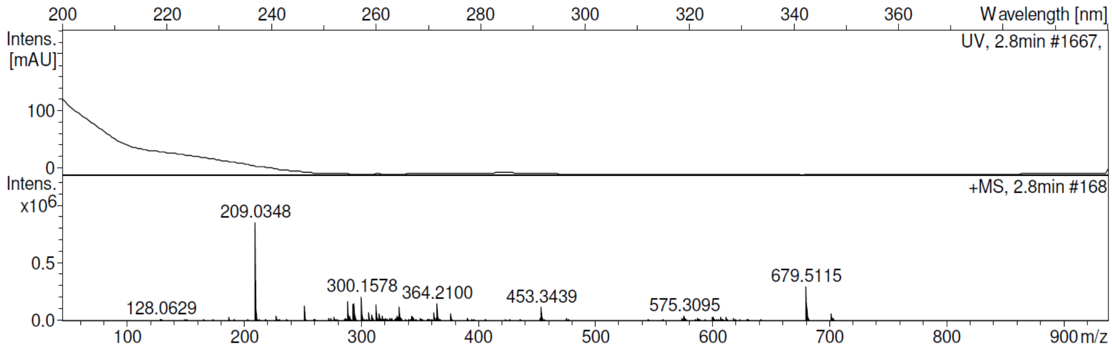

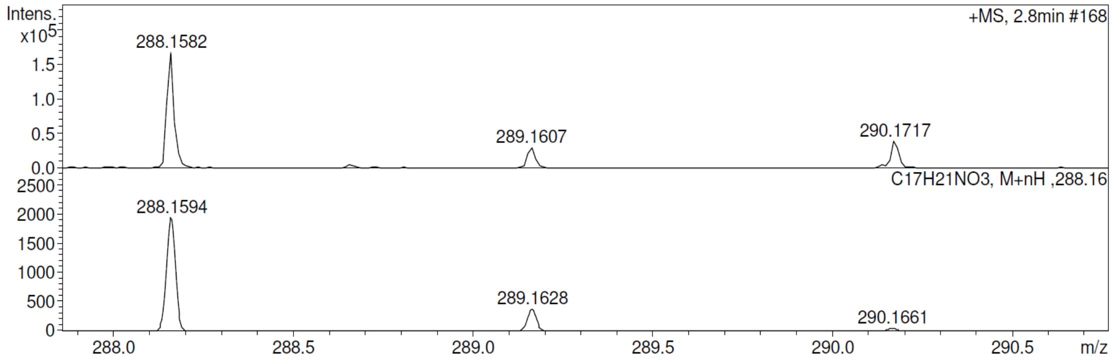

Bruker Compass DataAnalysis 4.0 printed: 2/7/2022 2:12:12 PM Page 1 of 1

HRMS (ESI, m/z): of compound **15b**

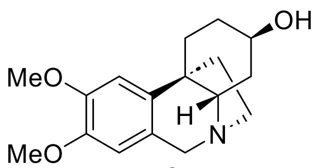

[(+)-**2c**]

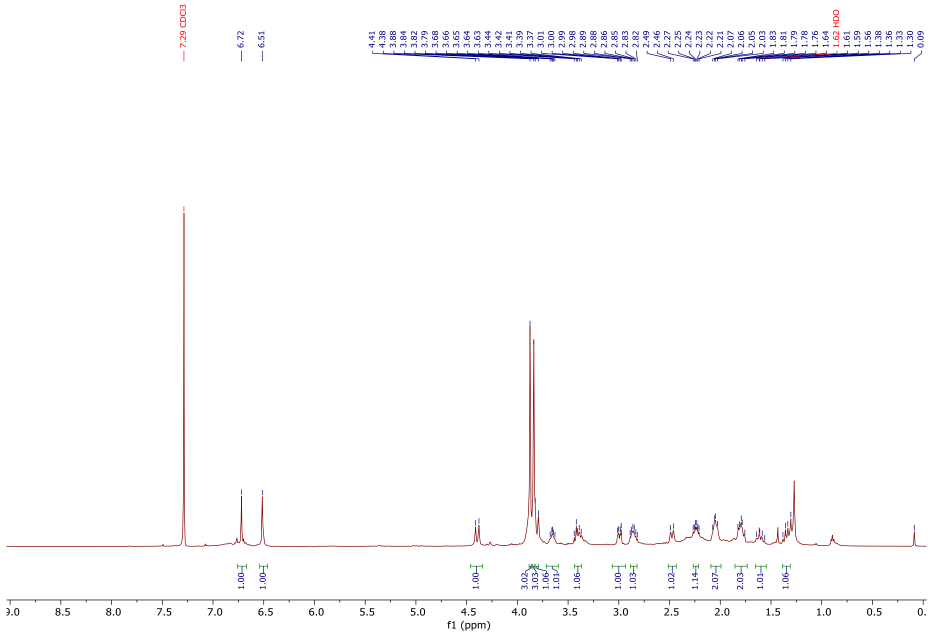

^{1}H NMR (500 MHz, $CDCl_3$) of compound **(+)-2c**

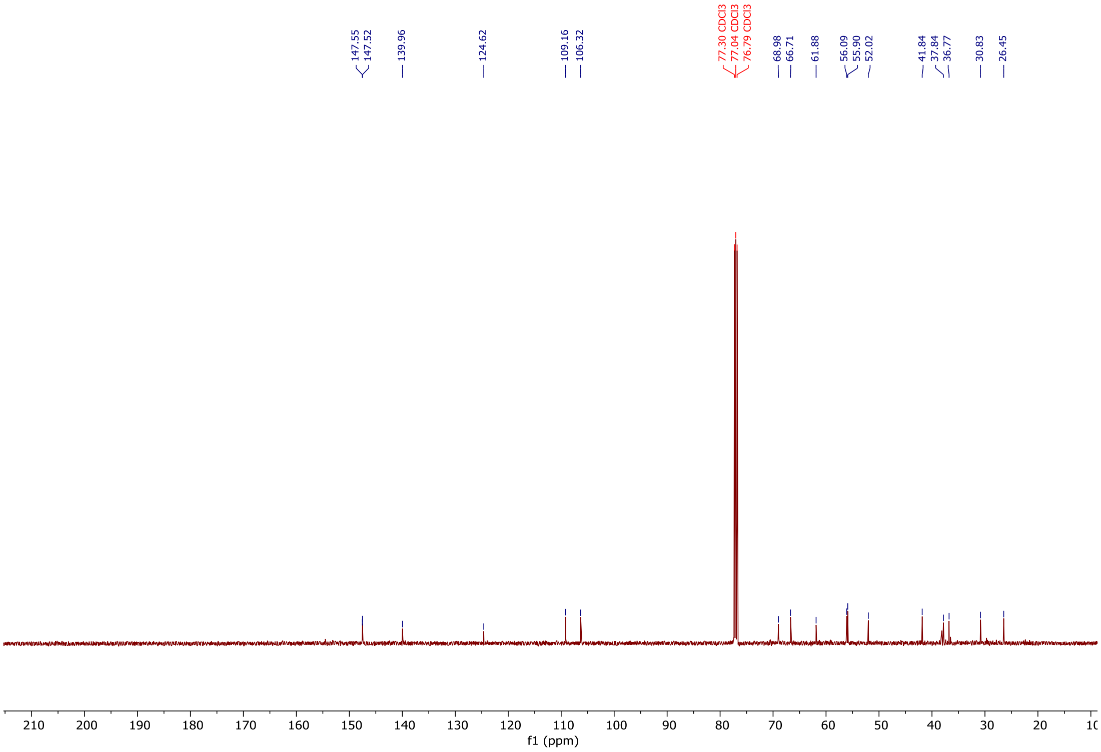

^{13}C{^{1}H} NMR (125 MHz, $CDCl_3$) of compound **(+)-2c**

Display Report

Analysis Info

Analysis Name	D:\Data\NEW USER DATA 2022\fe-2022\18-feb-2022\Dr.A.Bisai-AB-SM-02192_1-A,7_01_11180.d	Acquisition Date	2/18/2022 3:08:04 PM
Method	hrlcms-20 sept.m	Operator	RUCHI
Sample Name	Dr.A.Bisai-AB-SM-02192	Instrument	micrOTOF-Q II 10330
Comment			

Acquisition Parameter

Source Type	ESI	Ion Polarity	Positive	Set Nebulizer	1.2 Bar
Focus	Active	Set Capillary	4500 V	Set Dry Heater	200 °C
Scan Begin	50 m/z	Set End Plate Offset	-500 V	Set Dry Gas	6.0 l/min
Scan End	3000 m/z	Set Collision Cell RF	130.0 Vpp	Set Divert Valve	Waste

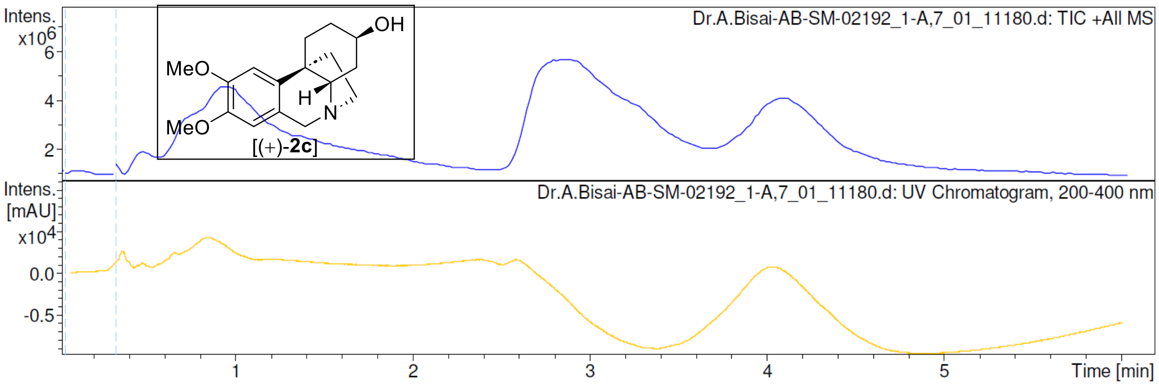

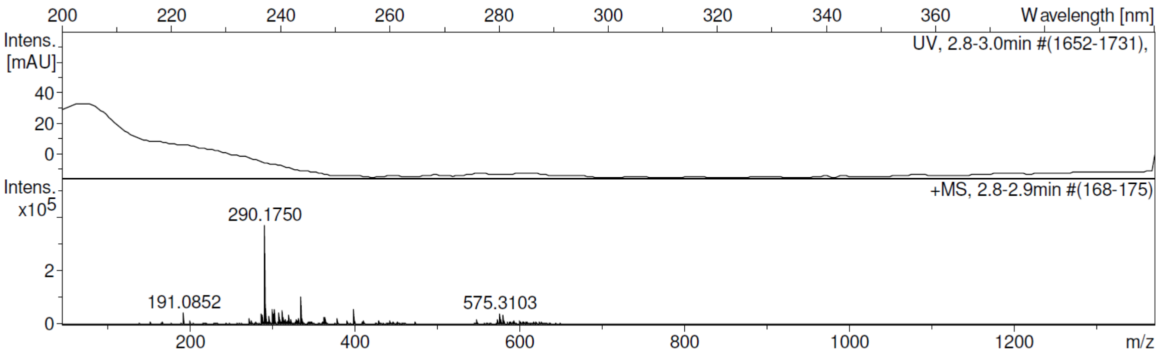

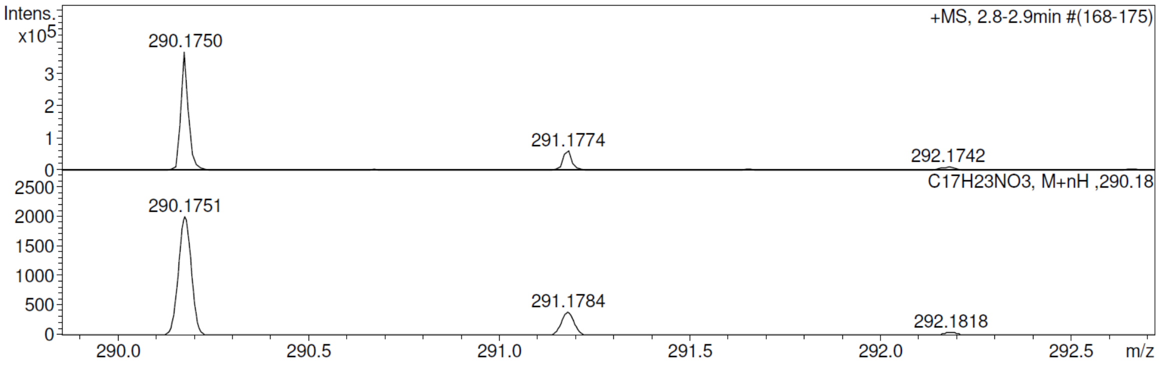

Bruker Compass DataAnalysis 4.0 printed: 2/18/2022 3:35:33 PM Page 1 of 1

HRMS (ESI, m/z): of compound **(+)-2c**
